# The role of activation of two different sGC binding sites by NO‐dependent and NO‐independent mechanisms in the regulation of *SACs* in rat ventricular cardiomyocytes

**DOI:** 10.14814/phy2.15246

**Published:** 2022-04-05

**Authors:** Andre G. Kamkin, Olga V. Kamkina, Andrey L. Shim, Andrey Bilichenko, Vadim M. Mitrokhin, Viktor E. Kazansky, Tatiana S. Filatova, Denis V. Abramochkin, Mitko I. Mladenov

**Affiliations:** ^1^ Department of Physiology Pirogov Russian National Research Medical University Moscow Russia; ^2^ Department of Human and Animal Physiology Lomonosov Moscow State University Moscow Russia; ^3^ Faculty of Natural Sciences and Mathematics Institute of Biology, “Ss. Cyril and Methodius” University Skopje Macedonia

**Keywords:** 8Br‐cGMP, ascorbic acid, BAY41‐2272, KT5823, L‐Arginine, nitric oxide, nitric oxide synthase, ODQ, patch‐clamp, SNAP, soluble guanylyl cyclase, stretch‐activated channels, ventricular cardiomyocytes

## Abstract

The mechanoelectrical feedback (MEF) mechanism in the heart that plays a significant role in the occurrence of arrhythmias, involves cation flux through cation nonselective stretch‐activated channels (*SACs*). It is well known that nitric oxide (NO) can act as a regulator of MEF. Here we addressed the possibility of *SAC’s* regulation along NO‐dependent and NO‐independent pathways, as well as the possibility of S‐nitrosylation of *SACs*. In freshly isolated rat ventricular cardiomyocytes, using the patch‐clamp method in whole‐cell configuration, inward nonselective stretch‐activated cation current *I_SAC_* was recorded through *SACs*, which occurs during dosed cell stretching. NO donor SNAP, α1‐subunit of sGC activator BAY41‐2272, sGC blocker ODQ, PKG blocker KT5823, PKG activator 8Br‐cGMP, and S‐nitrosylation blocker ascorbic acid, were employed. We concluded that the physiological concentration of NO in the cell is a necessary condition for the functioning of *SACs*. An increase in NO due to SNAP in an unstretched cell causes the appearance of a Gd^3+^‐sensitive nonselective cation current, an analog of *I_SAC_*, while in a stretched cell it eliminates *I_SAC_*. The NO‐independent pathway of sGC activation of *α* subunit, triggered by BAY41‐2272, is also important for the regulation of *SACs*. Since S‐nitrosylation inhibitor completely abolishes *I_SAC_*, this mechanism occurs. The application of BAY41‐2272 cannot induce *I_SAC_* in a nonstretched cell; however, the addition of SNAP on its background activates *SACs*, rather due to S‐nitrosylation.

ODQ eliminates *I_SAC_*, but SNAP added on the background of stretch increases *I_SAC_* in addition to ODQ. This may be a result of the lack of NO as a result of inhibition of NOS by metabolically modified ODQ. KT5823 reduces PKG activity and reduces *SACs* phosphorylation, leading to an increase in *I_SAC_*. 8Br‐cGMP reduces *I_SAC_* by activating PKG and its phosphorylation. These results demonstrate a significant contribution of S‐nitrosylation to the regulation of *SACs*.

## Graphical abstract

The endogenous production of NO by cardiac myocytes during the stretch directly modulates the SAC. We propose that the resultant production of NO exerts its action, independently of cGMP, most probably through S‐nitrosylation.

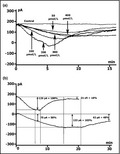

## 1 INTRODUCTION

It is well known that the electrophysiological properties of cardiomyocytes are sensitive to mechanical stress. This phenomenon commonly referred to as mechanoelectrical feedback (Lab, 1996), is believed to play a very important role in the pathophysiology of cardiac arrhythmias (Nazir & Lab, 1996; Ravens, 2003). In healthy hearts, the mechanism of mechanoelectrical feedback may involve transmembrane cation fluxes through stretch‐activated channels (*SACs*) (Craelius et al., 1988), which can cause modulation of the membrane potential of cardiac myocytes (Kamkin et al., 2000, 2003). It was shown previously that local stretch of single ventricular myocytes causes transmembrane current inflow enhancement (Kamkin et al., 2000, 2003). Stretch sensitivity is particularly high in hypertrophied ventricular cardiomyocytes from spontaneously hypertensive rats and in ventricular cardiomyocytes from patients with end‐stage heart failure (Kamkin et al., 2000). Similar to ventricular cardiac myocytes, mechanical stimulation of diseased atrial tissue can also cause rhythm disturbances, including muscle fibrillation. Thus, cardiomyocytes *SACs* play a very important role not only in the work of the heart but, above all, in pathological conditions. However, electrophysiological mechanisms that underlie the sensitivity of atrial myocytes to physical stretch are still unknown. One such mechanism may be the regulation of *SACs* by nitric oxide (NO).

Nitric oxide (NO)‐sensitive soluble‐guanylyl cyclase (sGC), catalyzes the formation of intracellular messenger cyclic guanosine monophosphate (cGMP) and is considered the main receptor for intracellular NO, produced by NO synthase (NOS) in cells (Boycott et al., 2020; Seddon et al., 2007). Primary activation of sGC starts with NO binding to the sixth hemo iron coordination site in heme nitric oxide/oxygen binding domain (HNOX) in β subunit, and subsequent breaking of the bond to His105. However, this is not the whole mechanism, as binding of a single molecule of NO leads to only a modest activation, which is enhanced several times by binding additional NO molecules to sites with lower affinity (Cary et al., 2005; Fernhoff et al., 2009; Martin et al., 2012). The location of these additional sites is not clear and may be found at hemo iron or a protein cysteine residue (Gileadi, 2014). At the same time, using photoaffinity labeling, cysteine 238 and cysteine 243 regions were determined in α1‐subunit of sGC and were determined as target places for a new different type of sGC stimulator. This type of sGC stimulator, BAY 41‐2272, works through a NO‐independent mechanism and produces intracellular messenger cGMP (Stasch et al., 2001).

It has been shown that NO causes activation of NO‐dependent mechanism of sGC stimulation, activate *Na*_V_ channels of ventricular and pacemaker myocytes, and cause different effects: activation or inhibition or both in *L*‐*type* Ca^2+^ channels, inhibition of *K*_V_ 4.1, *K*_V_ 4.4, *hK*_V_ 5.1 and *K*_V_ 11.1 channels, activation of *K*ir 2.1 channels, and modulation of work of *K*2P channels (Makarenko et al., 2012).

At the same time, it is widely known that isolated ventricular myocytes of mice, rats, and guinea pigs respond to local deformation (“stretch”) with activation of a nonselective cation conductance *G*_ns_ via mechanically gated channels, stretch‐activated channels (*SACs*), and via deactivation of inwardly rectifying potassium conductance *G*_K1_ (Dyachenko et al., 2009; Kamkin et al., 2000, 2003; Zeng et al., 2000).

The work of *SACs* was shown to be determined by the presence of NO in the cell, since in a stretched cell NO scavenger 2‐(4‐carboxyphenyl)‐4,4,5,5‐tetramethylimidazoline‐1‐1‐oxy‐3‐oxide (PTIO), completely blocked *I*_SAC_ induced by cell stretching, while its preliminary introduction did not cause any reaction to stretch. Furthermore, the use of NO synthase inhibitors, for example, NG‐Nitro‐L‐arginine methyl ester hydrochloride (L‐NAME), resulted in the absence of cardiomyocyte's reaction even at a stretch of 10 µm (Dyachenko, Husse, et al., 2009; Kazanski et al., 2010a; Makarenko et al., 2012).

The ventricular cardiomyocytes of wild‐type (WT) mice, NOS1^−/−^ and NOS2^−/−^ knockout mice, were shown to respond similarly to discrete cell stretching with a discrete increase in *I*_SAC_, while cells from NOS3^−/−^ knockout mice did not respond even to a stretch of 10 µm (Kazanski et al., 2010b; Makarenko et al., 2012). As previously shown by Western blot and RT‐PCR analysis, expression of NOS3 in mice's ventricular cardiomyocytes takes about 20% of the total amount of NOS3 in the heart.

Finally, it was shown that NO donors such as S‐Nitroso‐N‐acetyl‐D, L‐penicillamine (SNAP), and Diethylammonium (Z)‐1‐(N, N‐diethylamino)diazen‐1‐ium‐1,2‐diolate (DEA‐NO) cause activation of the nonselective current (*I*_L,ns_), determined by *SACs*, even without stretching. And on the background of a stretched cell, the use of exogenous NO causes *I_SAC_* inhibition (Kazanski et al., 2010b). Based on fact that cell stretching possibly activates NOS, it has been suggested that *SACs’* function is determined by intracellular NO concentration [NO]_in_ (Makarenko et al., 2012; Kamkin et al., 2010; Kazanski et al., 2010a).

Based on data presented and published earlier, we concluded that the physiological concentration of NO in the cell is a necessary condition for the operation of *SACs*. An increase in NO concentration due to exogenous addition of donors, on one hand, causes the appearance of a Gd^3+^‐sensitive nonselective cation current *I*_L,ns_, an analog of *I_SAC_* in an unstretched cell. On other hand, it eliminates stretch‐activated current, *I_SAC_*, in a stretched cage. NO‐dependent pathway of sGC activation through β subunit triggered by SNAP is important for regulation of *SACs*, but also and NO‐independent pathway of activation through *α* subunit triggered by BAY41‐2272. However, S‐nitrosylation of these channels is the most important component of the regulation of *SACs*, since inhibitor of S‐nitrosylation eliminates *I*_SAC_ induced by cell stretch. Application of BAY41‐2272 cannot induce *I*_SAC_ in a nonstretched cell; however, the addition of SNAP on its background activates *SACs*, rather due to S‐nitrosylation. ODQ eliminates cell stretch‐induced *I*_SAC_. However, SNAP added on the background of stretch in addition to ODQ increases *I*_SAC_, which can only be altered by a metabolic transformation of ODQ under NO deficient conditions as a result of NOS inhibition. PKG inhibitor KT5823 reduces PKG activity and reduces phosphorylation of *SACs*, leading to a transient increase in *I*_SAC_, while the introduction of SNAP reduces *I*_L,ns_ to an even greater extent, since the cell was initially stretched. 8Br‐cGMP reduces *I*_SAC_, as it should, by activating PKG and therefore inducing phosphorylation. Similarly, KT‐5823, by inhibiting PKG, increases *I*_SAC_. Thus, KT‐5823 and 8Br‐cGMP have a characteristic effect on cell stretch‐induced *I*_SAC_. Finally, the results of our study demonstrated a significant contribution of S‐nitrosylation to the regulation of *SACs*.

## 2 MATERIALS AND METHODS

### 2.1 Animals

All experiments conformed to the Guide for the care and use of laboratory animals published by the US National Institutes of Health (8th edition, 2011). The experimental protocol was approved by the ethics committee of the Russian National Medical Academy. Male outbred white rats weighing 220–270 g (*n* = 124) were held in animal house for 4 weeks under a 12:12 h light: The dark period in standard T4 cages before the experiment and fed *ad libitum*.

### 2.2 Solutions

Ca^2+^‐free physiological salt solution (Ca^2+^‐free PSS) containing in (mmol/L): 118 NaCl, 4 KCl, 1 MgCl_2_, 1.6 NaH_2_PO_4_, 24 NaHCO_3_, 5 Sodium pyruvate, 20 taurine, and 10 glucose, adjusted to pH 7.4 with NaOH (bubbled with carbogen 95% O_2_ + 5% CO_2_) (Gödecke et al., 2001; Kamkin et al., 2003). Enzyme medium containing: Ca^2+^‐free PSS supplemented with 10 µmol/l CaCl_2_, 0.2 mg/ml collagenase (Type II, Worthington, 225 units/mg), 1 mg/ml bovine serum albumin (Sigma) (Gödecke et al., 2001; Kamkin et al., 2003). Before the actual experiments, the cells were stored for at least 2 hours in modified Kraftbrühe (KB) ‐ medium, containing in (mmol/L): 50 L‐glutamic acid, 30 KCl, 3 MgSO_4_ × 7H_2_O, 20 taurine, 10 glucose, 30 KH_2_PO_4_, 0.5 EGTA, 20 HEPES, adjusted to pH 7.3 with KOH (Gödecke et al., 2001; Kamkin et al., 2003). Isolated cells were stored in KB‐solution for up to 8 h. Ventricular cardiomyocytes were perfused with (37°C) solution containing (mmol/L): 150 NaCl, 5.4 KCl, 1.8 CaCl_2_, 1.2 MgCl_2_, 20 glucose, and 5 HEPES, at pH of 7.4 adjusted with NaOH (K_out_ solution). In some experiments e.c. 5.4 mmol/L KCl was replaced by 5.4 mmol/L CsCl (Cs_out_ solution). The Cs^+^‐based solution was used to confirm that recorded stretch‐modulated and SNAP‐modulated currents are not carried out by K^+^ ions. Internal pipette solution contains in (mmol/l): 140 KCl, 5 Na_2_ATP, 5 MgCl_2_, 0.01 EGTA, 10 Hepes/KOH and, pH 7.3. In some experiments, 140 mmol/L KCl was replaced by 140 mmol/L CsCl ([Cs]_in_ solution).

### 2.3 Compounds

SNAP at concentrations of 50–400 µmol/L was used as a NO donor. SNAP causes sGC activation and the formation of cGMP via NO‐dependent pathway, and as shown below, the optimal concentration for such activation is equal to 200 µmol/L. It is known that 100 µmol/L SNAP releases 1.4 µmol/L NO per minute at 37°C, and this value is linear over a wide range of concentrations (Feelisch, 1991). Also, when determining the concentration of NO by using heliotropic NO traps, it was shown that 16 µmol/L NO is released from 5 mmol/L SNAP within 60 min (Ioannidis et al., 1996), while by using a spectrophotometric method for determination, it was shown that 31 µmol/L NO is released from 5 mmol/L SNAP within 15–20 min (Ioannidis et al., 1996). Concentrations we used are permissible and are used by other authors in works on isolated cardiomyocytes (e.g. Tastan et al., 2007; Yoshida et al., 2020; Zhang et al., 2007). For stimulation of sGC and formation of cGMP via NO‐independent pathway, the compound, BAY 41‐2272 was employed at a concentration of 10 µmol/L. In part of the experiments, 5 µmol/L GdCl_3_ was added to the salt solution to block stretch‐activated channels and *I*_ns_, respectively. However, the definition of *I*_ns_ by its block by Gd^3+^ in $K_{\text{out}}^{+}$/$K_{\text{in}}^{+}$ solutions is questionable because Gd^3+^ interferes with Ca^2+^ ‐ and K^+^‐currents (Belus & White, 2002; Hongo et al., 1997). However, in ${\text{Cs}}_{\text{out}}^{+}$/${\text{Cs}}_{\text{in}}^{+}$ solutions such a definition of *I*_ns_ is possible (Shim et al., 2019).

### 2.4 Isolated cardiomyocyte preparation

We used the previously described cell isolation procedure (Kamkin et al., 2000, 2003) with slight modifications. Rats were anesthetized with an intraperitoneal injection of 80 mg/kg ketamine and 10 mg/kg xylazine. Heparin (1000 U/kg) was added to the anesthetics solution to prevent blood coagulation in coronary vessels of the excised heart. The chest was opened and the heart was rapidly excised and attached to a Langendorff apparatus (constant flow of 1 ml/min, 37°C) for flushing coronary vessels in Ca^2+^‐free PSS bubbled with carbogen for 5 min. After an initial perfusion period with Ca^2+^‐free PSS, hearts were perfused in a retrograde manner for 18–20 min with the same PSS, supplemented with Worthington type II collagenase (0.5 mg/ml), 1 mg/ml bovine serum albumin (Sigma), and 10 µmol/L CaCl_2_. The perfusate was continuously bubbled with carbogen (95% O_2_–5% CO_2_) and the temperature was equilibrated at 37°C. Then enzymes were washed out with a modified KB medium (Dyachenko, Husse, et al., 2009; Gödecke et al., 2001) (5 min), and the heart was disconnected from the perfusion system. Finally, ventricles were cut off, chopped, and gently triturated to release cells into the KB medium. The resulting cell suspension was filtered and stored in KB medium before use (22°C, 2 h).

### 2.5 Mechanical stretch of ventricular myocytes

The present type of mechanical stimulation has been described in detail before (Dyachenko, Husse, et al., 2009; Dyachenko et al., 2009; Kamkin et al., 2000, 2003), but here we described only those peculiarities that are important for the present context. After whole‐cell access of patch pipette (P), a fire‐polished glass stylus (S) was attached to the membrane (Dyachenko, Husse, et al., 2009; Dyachenko, Rueckschloss, et al., 2009; Kamkin et al., 2000, 2003). When the stylus was freshly polished and the surface membrane was clean, attachment succeeded in approximately 70% of attempts. The stylus was then lifted 2 µm to prevent “scratching” of the lower cell surface on the coverslip during the stretch. A motorized micromanipulator (MP 285, Sutter, Novato, Calif, USA, accuracy 0.2 µm) increased S‐P distance stepwise by up to 12 µm, with P being fixed point (Dyachenko, Husse, et al., 2009; Kamkin et al., 2000). Stretch and release of stretch could be repeated on average 3–4 times with the same cell. We have shown that our method stretches the cell surface locally, whereby the membrane in the line between P and S was stretched as expected (approx. 80% of the whole membrane surface remains unaffected) (Dyachenko, Husse, et al., 2009; Kamkin et al., 2000). The effect of mechanical stretching on the sarcomere pattern was imaged by a slow‐scan CCD camera (Princeton Instruments, Trenton, NJ, USA) and evaluated by MetaMorph software (Universal Imaging, West Chester, PA, USA). S and P were positioned 40 µm apart, before attaching them to the cell. The cell stretching by 4 µm (increasing the S.‐P distance) increased the local stretch about 6%, by 6 µm about 10%, by 8 µm about 14%, and by 10 µm about 18%. These values were less than expected but close to those previously obtained in isolated mouse cardiomyocytes. Presumably, the extent of local stretch decays from the cell surface to the interior of the cell where the optical focus was set (Dyachenko, Husse, et al., 2009; Kamkin et al., 2003).

### 2.6 Whole‐cell patch‐clamp

The whole‐cell patch‐clamp recording of *K* ^*+*^ and *Ca* ^*2+*^ currents was performed by using Axopatch 200B Amplifier and pClamp 10 software (Molecular Devices, San Jose, CA, USA). Data were filtered at 2 kHz, sampled at 5 kHz, and evaluated using the software. The myocytes were superfused in a small recording chamber (RC‐26; Warner Instrument Corp, Brunswick, CT, USA; volume 150 µl) mounted on an inverted microscope with an external $K_{\text{out}}^{+}$ solution or ${\text{Cs}}_{\text{out}}^{+}$ solution.

The borosilicate glass patch‐clamp electrodes had tip resistances between 1.5 and 2.5 MΩ, when filled. After seal formation, cell access was obtained by rupture of the patch. Pulses (140 ms) were applied at 1 Hz, and they started from a holding potential of −45 mV that caused inactivation of the tetrodotoxin (TTX) ‐sensitive *Na* ^+^ currents. The currents in response to trains of short (5 mV) pulses, applied at −45 mV, were taken for evaluation of the membrane capacitance and access resistance, whereby compensation for the capacitive and leak currents was not applied. Since the amplitude of the currents depends on the cell's length and diameter (the cardiomyocyte's diameter from control rats was about 25 ± 6 µm), cells of similar geometry always were selected; on average these cells had a membrane capacitance of 150 ± 16 pF (*n* = 16). In 16 representative cells, input resistance was about 58 ± 5 MΩ. The effect of a different size of the stretched membrane was minimized by adjusting glass tools to the same 40 µm S‐P distance, before the application of stretch. Since mechanical stretching of the cell was restricted to a small unknown area between S and P, we did not divide the stretch‐induced currents by the membrane capacitance. Measurements usually lasted for approximately 30 min, during which time, access resistance and capacitive current remained stable. Current/voltage relations (*I*/*V* curves) were obtained by application of a series of 20 pulses with 140 ms duration each, starting from a holding potential of −45 mV. Membrane currents at the end of pulse (“late currents”) were plotted as functions of respective clamp step potential. Seal resistance remained constant, that is, it was 1.5 ± 0.3 GΩ before and 1.4 ± 0.4 GΩ during the stretch. Also, access resistance and membrane capacitance remained unaffected. Hence, the stretch‐induced inward current should be attributed to activation of an ionic current and not to leakage around the seal. The intercept of the resulting *I*/*V* curve with voltage axis defined zero current potential (*E*_0_) that corresponded to the resting membrane potential of a nonclamped cell (between −70 and −80 mV). Also, online records of net membrane current were carried out at the level of a holding potential of −45 mV (time‐course) (Boycott et al., 2013; Dyachenko, Husse, et al., 2009; Kamkin et al., 2003).

The values of the differential current calculated as the difference between the control current values and current values obtained on the background of cell stretch or their exposure at −45 or −80 mV (^C−S^ *ΔI*) are denoted as *I*_SAC(−45)_ and *I*_SAC(−80)_ (Kamkin et al., 2000, 2003).

### 2.7 Statistics

Values are given as means ± SD. Significant differences were detected by *Analysis of Variance (ANOVA)* with the Bonferroni test as a post hoc test. *Two*‐*way ANOVA* was also employed in cases, where more than one factor was evaluated. Significance was assumed at *p* < 0.05.

## 3 RESULTS

### 3.1 Local stretch induces net inward currents: (time course and voltage dependence)

**FIGURE 1:**
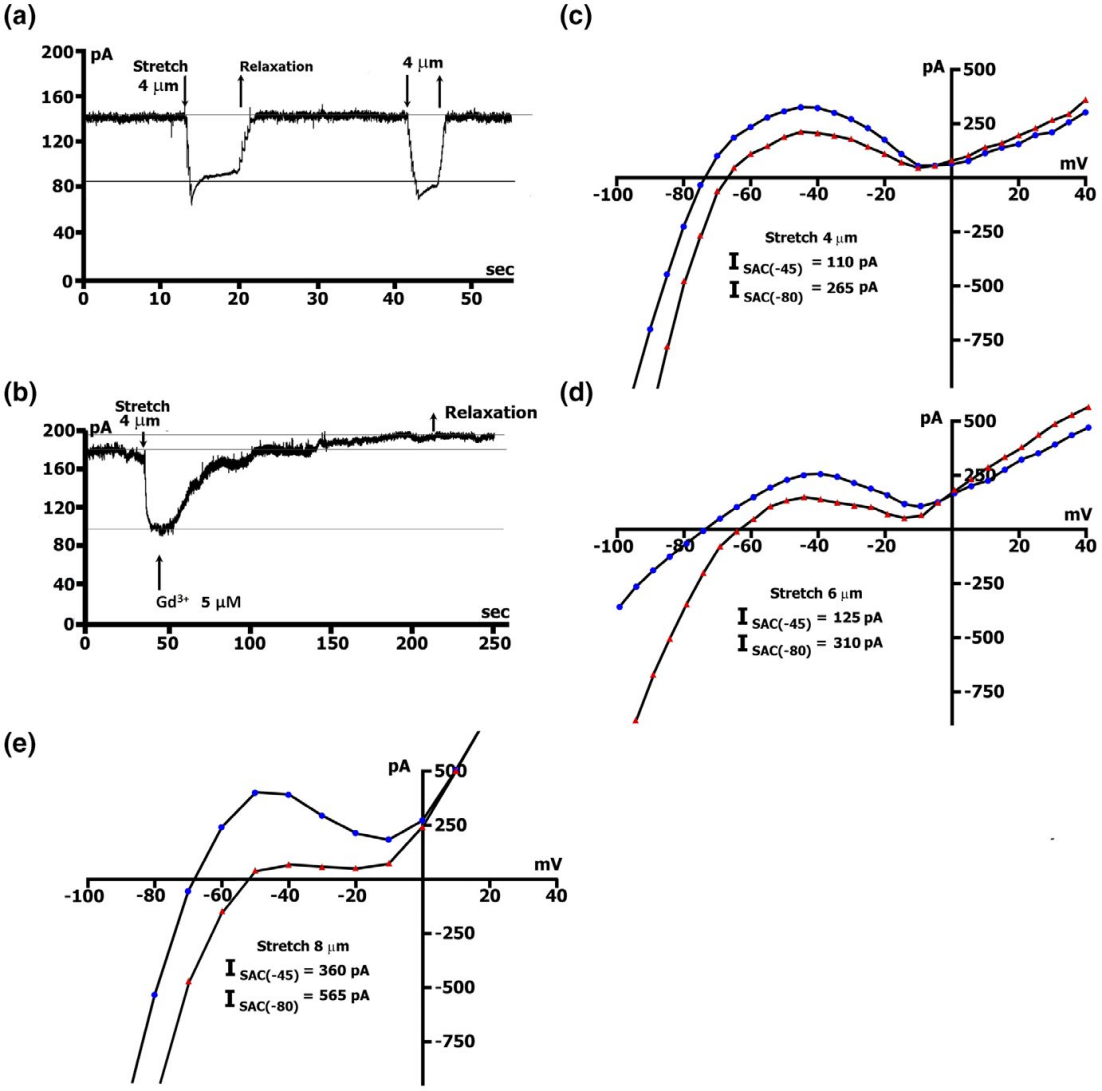
Induction of net inward currents by a local stretch. (a) Online records (time course) of membrane current, K^+^ currents not suppressed. *V*_m_ clamped to a holding potential of −45 mV. The amplitude of stretch (4 µm) and amount of stretch‐induced inward current at −45 mV indicated. (b) Gadolinium completely blocks the stretch‐induced inward current. (c, d, e) Graduation by the extent of stretch. The amplitude of negative current (at −80 mV), reduction of the positive hump of the *I*/*V* curve (at −55 to −60 mV), and value of depolarization (change of the zero current potential *E*_0_, i.e., intercept of *I*/*V* curve with current axis) increase with the value of stretch; *I*/*V*‐curves before (circles) and during the stretch (triangles) of 4‐µm (c), 6‐µm (d) and 8‐µm (e)

Figure 1 shows membrane currents in $K_{\text{out}}^{+}$/$K_{\text{in}}^{+}$ solutions using online records (time course). Unlike in mouse cardiomyocytes (Kamkin et al., 2003), 4 µm stretch in the rat heart cells changed the holding current at −45 mV (holding potential) by 0.054 nA (0.071 ± 0.003 nA) to more negative values. The stretch‐induced current change was completed during the period of mechanical movement (usually 200 min); the activation time course was not observed. A stretch of 2 µm did not change the currents (not illustrated). Online records during stretches of 4, 6, 8, 10, and 12 µm showed an increase in the negative current as a consequence of the extent of stretch (Table 1 ‐ row A). During the continuous stretch, inward current remained constant; inactivation with time was not observed. The effect of stretch on the current was reversible; current returned to value before stretching when stretch was relaxed by returning stylus (S) to its position before stretching. The experiments were ended by adding 5 µmol/l Gd^3+^ to the superfusing solution on the background of continuous application of stretch (Figure 1b). In 1.5–2 mins from its application, Gd^3+^ always caused a shift of the stretch‐induced inward current in a positive direction compared to the control current before stretch.

**TABLE 1:** The amplitude of currents through stretch‐activated nonselective cation channels *I*_SAC_ (*ΔI*_ns_, in nA) at −45 and −80 mV, dependence on the extent of local stretch (in µm) and the ionic composition. Mean ± SD, *n* = number of experiments, m = number of animals. Rows A‐D: A ‐ Net membrane current during stretch by means of online records (time‐course). Holding potential (*V*_h_) −45 mV. K^+^ currents unblocked $K_{\text{in}}^{+}$/$K_{\text{out}}^{+}$ solutions. B ‐ Difference net current *I*_SAC_ described from *I*/*V*‐curves (*I*_L_) before and during the stretch. $K_{\text{in}}^{+}$/$K_{\text{out}}^{+}$ solutions. C ‐ *I*_SAC_ during the stretch (time course). *V*_h_ = −45 mV, ${\text{Cs}}_{\text{in}}^{+}$/${\text{Cs}}_{\text{out}}^{+}$ solutions (K^+^ currents blocked by Cs^+^, electrode solution and bathing solution containing CsCl instead of KCl). D − *I*_SAC_ described from *I*_L_ before and during the stretch. In all cases, *p* < 0.01

Stretch, (μm)		4					6					8				
Solutions		*m*	*n*	*I*_SAC(−45)_, (nA)	*n*	*I*_SAC(−80)_, (nA)	*m*	*n*	*I*_SAC(−45)_, (nA)	*n*	*I*_SAC(−80)_, (nA)	*m*	*n*	*I*_SAC(−45)_, (nA)	*n*	*I*_SAC(−80)_, (nA)
A	$K_{\text{in}}^{+}/K_{\text{out}}^{+}$	9	39	−0.071 ± 0.003	–	–	10	36	−0.195 ± 0.009	–	–	8	21	−0.441 ± 0.017	–	–
B	$K_{\text{in}}^{+}/K_{\text{out}}^{+}$	4	12	−0.082 ± 0.007	12	−0.26 ± 0.013	4	8	−0.176 ± 0.019	8	−0.29 ± 0.08	4	6	−0.398 ± 0.012	6	−0.58 ± 0.03
C	${\text{Cs}}_{\text{in}}^{+}$/${\text{Cs}}_{\text{out}}^{+}$	4	5	−0.031 ± 0.007	–	–	3	5	−0.082 ± 0.011	–	–	3	6	−0.155 ± 0.024	–	–
D	${\text{Cs}}_{\text{in}}^{+}$/${\text{Cs}}_{\text{out}}^{+}$	3	6	−0.033 ± 0.004	6	−0.05 ± 0.010	4	6	−0.078 ± 0.012	6	−0.14 ± 0.01	3	5	−0.156 ± 0.023	5	−0.23 ± 0.03

Stretch, (µm)		10					12				
Solutions		*m*	*n*	*I*_SAC(−45)_, (nA)	*n*	*I*_SAC(−80)_, (nA)	*m*	*n*	*I*_SAC(−45)_, (nA)	*n*	*I*_SAC(−80)_, (nA)
A	$K_{\text{in}}^{+}$/$K_{\text{out}}^{+}$	9	23	−0.895 ± 0.037	–	–	4	9	−1.548 ± 0.160	–	–
B	$K_{\text{in}}^{+}$/$K_{\text{out}}^{+}$	3	5	−0.842 ± 0.077	5	−1.45 ± 0.22	3	6	−1.673 ± 0.284	6	−2.225 ± 0.18
C	${\text{Cs}}_{\text{in}}^{+}$/${\text{Cs}}_{\text{out}}^{+}$	5	10	−0.329 ± 0.022	–	–	4	7	−0.978 ± 0.126	–	–
D	${\text{Cs}}_{\text{in}}^{+}$/${\text{Cs}}_{\text{out}}^{+}$	4	5	−0.334 ± 0.026	5	−0.47 ± 0.045	3	4	−0.926 ± 0.245	4	−1.354 ± 0.21

The current‐voltage (*I*/*V*) relation of recordings was measured at the end of the pulse (late current *I*_L_). The intercept of the *I*/*V*‐curve with the zero‐current axis is zero‐current potential *V_0_*, which is equivalent to diastolic membrane potential under the current clamp. To analyze underlying mechanisms, we separated the *I*/*V* curves of net and differential currents into different components by curve fitting. The net current was described by the superimposition of the *I*_L,ns_ (current through stretch‐activated nonselective cation channels), *I*_K1_ (inwardly rectifying potassium current), and *I*_oth_ (presumably sum of several outwardly rectifying currents, for instance, K^+^ currents through two‐pore‐domain potassium (*TREK*), channels (Li et al., 2006; Patel et al., 2005) or outwardly rectifying canonical transient receptor potential‐6 (*TRPC6*), channels (Hofmann et al., 1999; Spassova et al., 2006).

Without stretch, the *I*/*V* ‐ curves intersected voltage axis at *V_0_* = −74.3 ± 0.4 mV (*n* = 127), a value corresponding to the diastolic potential. At *V_0_*, potassium current *I*_K_ was zero. Hence, the stretch‐induced negative current *I*(*E*_K_) should be attributed to the stretch activation of *G*_ns_. Between −100 and −74.3 ± 0.4 mV, stretch reduces the slope of the *I*/*V* curve, which was attributed to the stretch‐induced deactivation of *G*_K1_ (Dyachenko, Rueckschloss, et al., 2009).

During stretches of 4, 6, 8, 10, or 12 µm, the amount of negative *I*_L,ns_ increases to the extent of stretch (Table 1 ‐ row B). The differential current values are calculated as the difference between control current values and current values on the background of cell stretch or other action (results labeled with a *Δ*) at −45 and −80 mV (*^C^* ^/^ *^S^ΔI*_(−45)_ and *^C^* ^/^ *^S^ΔI*_(−80)_), designated as *I*_SAC(−45)_ and *I*_SAC(−80)_. The voltage dependence of *I*_L_ and its modulation by stretch is shown as an example on the *I*/*V* curves in Figure 1c, d, and e. Before stretch (circles), the *I*/*V* curve was *N*‐shaped and crossed voltage axis (zero current potential *V_0_*) at −75 mV (−74.3 ± 0.4 mV, *n* = 127; equivalent to resting potential of the non‐clamped cell). The modest 4 µm stretch (Figure 1c, triangles, Table 1 ‐ row B) shifted the net currents to more negative values, and *V_0_* changed to −67 mV (Table 2 ‐ row A). The minus sign (−) for *I*_SAC(−45)_ emphasizes that cell stretch leads to more negative values of the initial net current at the level of holding potential *V*_h_ = −45 mV, while for *I*_SAC(−80)_ it indicates an increase in the net current in response to stretch. Close to −5 mV, the *I*/*V* curves recorded before and during the stretch crossed each other, and at positive potentials, the late current increased by the stretch. Stretch by 6 µm shifted the *I*/*V* curve to more negative currents than stretch by 4 µm (see Table 1 ‐ row B; triangles in comparison to circles in Figure 1d) and depolarized *V*_0_ to −61 mV (Table 2 ‐ row A). The 8 µm stretch further depolarized *V*_0_ to −50 mV (Figure 1e; Table 2 ‐ row A), and increased *I*_SAC(−45)_ and *I*_SAC(−80)_ to more negative currents than 6 µm stretch (Table 1 ‐ row B). A further increase in the extent of stretch leads to even greater changes in the *I*_SAC(−45)_, *I*_SAC(−80)_ (see Table 1 ‐ row B), and depolarization of *V*_0_ (see Table 2 ‐ row A).

**TABLE 2:** Zero‐current potential (*V*_0_) ‐ The intercept of the *I*/*V* curves with the zero‐current axis before and during a stretch in different ionic composition. Mean ± SD, *n* = number of experiments, *m* = number of animals. Rows A‐B: A ‐ K^+^ currents unblocked, $K_{\text{in}}^{+}$/$K_{\text{out}}^{+}$ solutions. B ‐ ${\text{Cs}}_{\text{in}}^{+}$/${\text{Cs}}_{\text{out}}^{+}$ solutions. A *p* > 0.05 was considered to indicate a statistically nonsignificant difference (*p* = NS). All other instances with *p* < 0.01 are not indicated. ^#^ *p* = NS versus stretch 6 µm, *p = NS versus stretch 4 µm

Stretch, µm		Control			4			6			8			10			12		
(ionic composition)		*m*	*n*	*V*_0_, (mV)	*m*	*n*	*V*_0_, (mV)	*m*	*n*	*V*_0_, (mV)	*m*	*n*	*V*_0_, (mV)	*m*	*n*	*V*_0_, (mV)	*m*	*n*	*V*_0_, (mV)
A	$K_{\text{in}}^{+}$/$K_{\text{out}}^{+}$	31	127	−74.3 ± 0.4	4	12	−65.3 ± 2.3^#^	3	8	−61.5 ± 2.8	4	6	−52.7 ± 3.2	4	5	−43.1 ± 2.5	5	6	−32.0 ± 2.1
B	${\text{Cs}}_{\text{in}}^{+}$/${\text{Cs}}_{\text{out}}^{+}$	4	11	−39.6 ± 1.4*	3	6	−37.6 ± 1.6^#^	3	6	−35.1 ± 1.3	4	5	−29.3 ± 1.7	4	5	−22.8 ± 2.2	4	4	−15.4 ± 2.4

### 3.2 Local stretch activates current through nonselective cation channels (*I*_L,ns_: time course and voltage dependence)

**FIGURE 2:**
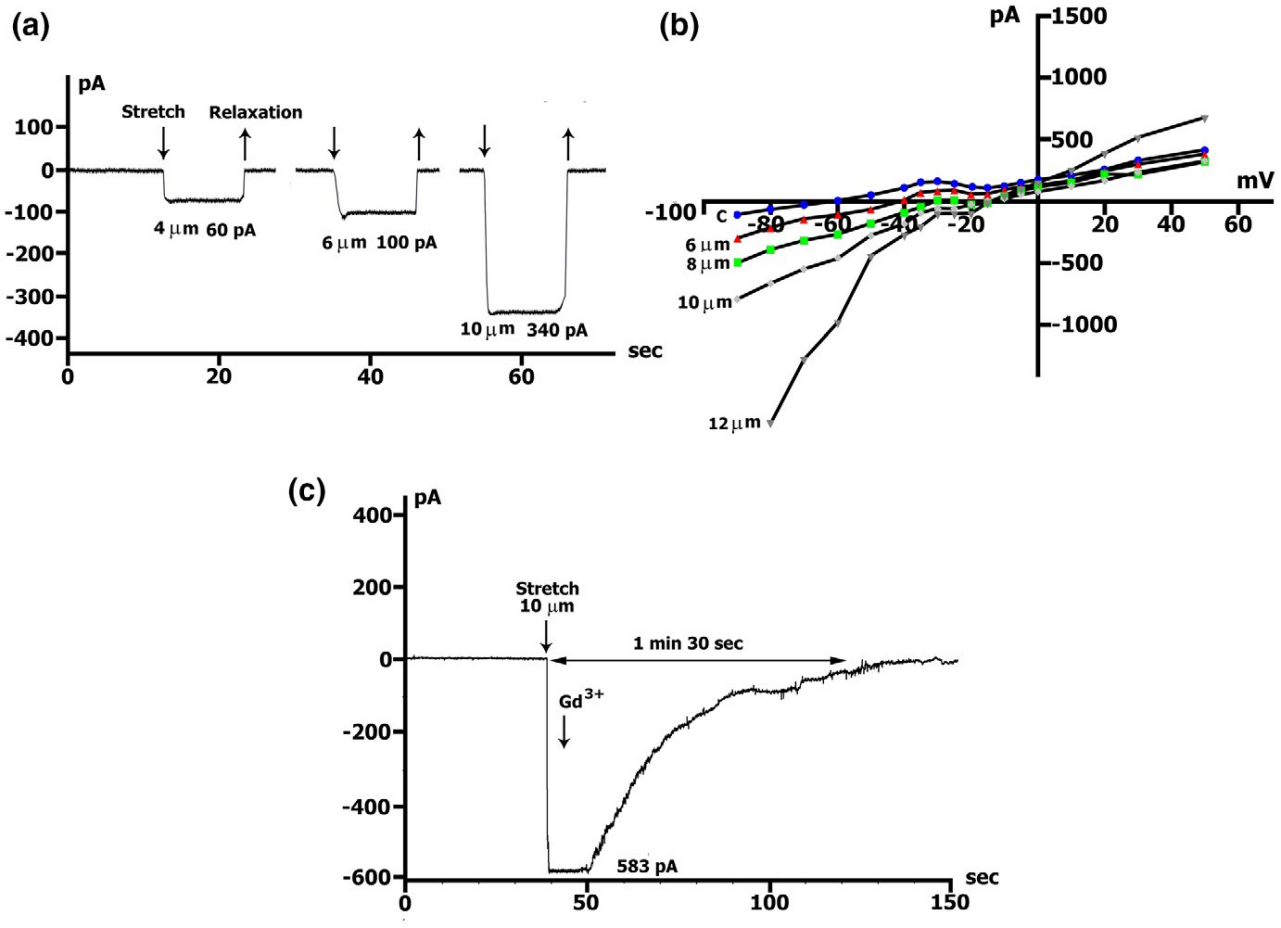
Local stretch activates *I*_SAC_, (the current through nonselective cation channels). (a) Online records of *I*_SAC_, K^+^ currents suppressed. *V*_m_ clamped to a holding potential of −45 mV. Graduation by the extent of stretch. As an example, the values of stretching 4, 6, and 10 µm are shown. (b) Graduation by the extent of stretch. The amplitude of *I*_SAC_ (at −45 mV and −80 mV), and value of depolarization (change of the zero current potential *V*_0_) increase with the value of stretch; *I*/*V*‐curves before (circles) and during the stretch of 6‐µm (triangles), 8‐µm (squares), 10‐µm (rhombuses), 12‐µm (inverse triangles). (c) Online records of *I*_SAC_, gadolinium completely blocks *I*_SAC_. Stretch by 10 µm as an example

Figure 2a shows membrane currents recorded in ${\text{Cs}}_{\text{out}}^{+}$/${\text{Cs}}_{\text{in}}^{+}$ solutions (time course). Unlike in mouse cardiomyocytes (Kamkin et al., 2003), in rat cardiomyocytes, only 4 µm stretch shifted the holding current at −45 mV to negative values (Figure 2a; Table 1 ‐ row C). A Stretch of 2 µm did not change the currents (not illustrated). During stretches of 4, 6, 8, 10, or 12 µm the amount of negative current increased in consequence to the extent of stretch (Figure 2a and b; Table 1 ‐ row C). During the continuous stretch, inward current remained constant and the effect of stretch on the current was reversible. Experiments were ended by adding 5 µmol/L Gd^3+^ to the superfusing solution during continuous stretch application (Figure 2c). Gd^3+^ returned stretch‐induced inward current to resting value in 1.5–2 min from its application. With suppressed K^+^ currents before stretch, the *I*/*V* relation of the late currents was flat and had *V*_0_ of about −40 mV (Table 2 ‐ row B, Figure 2b, circles). The discrete cell stretch also gradually shifted *V*_0_ toward depolarization up to −15 mV when the cell was stretched by 12 µm (Table 2 ‐ row B). The modest 4 µm stretch (not shown) caused *I*_L,ns_ shift to negative values, and further discrete stretch of the cell also gradually shifted *I*_L,ns_ to negative values (Figure 2b), so that *I*_SAC(−45)_ and *I*_SAC(−80)_ increased proportionally to the extent of stretch (Table 1 ‐ row D). The stretch‐activated differential current *I*_SAC_ had an almost linear voltage dependence and reversed at −5 mV. At positive clamp steps, currents were shifted in the outward direction. The mean values of *I*_SAC_ for clamp potentials of −45 and −80 mV are listed in Table 1 ‐ row D, consequently for stretches of 4, 6, 8, 10, and 12 µm. The more negative clamp potential increased the amplitude of *I*_SAC_, as can be expected if the larger driving force (difference between membrane potential *V*_m_ and reversal potential *E*_rev_) is multiplied by a voltage‐independent conductance *G*_SAC_.

### 3.3 Participation of NO in modulation of the membrane currents *I*_L,ns_, and *I*_K1_

**FIGURE 3:**
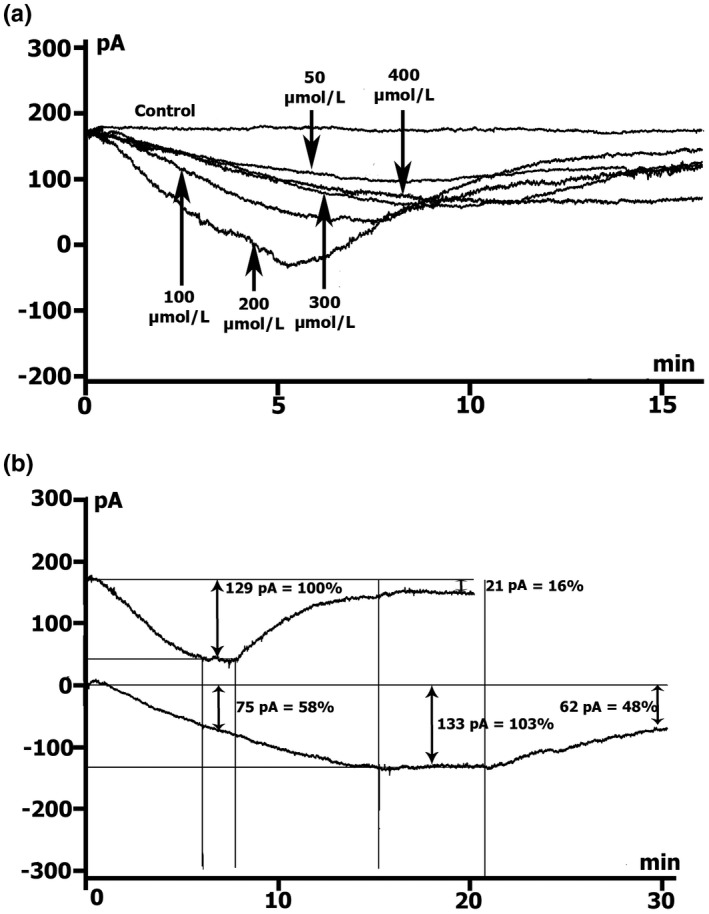
SNAP shifted net currents to more negative values. Online records (time course) of membrane current, *V*_m_ clamped to a holding potential of −45 mV. (a) Different concentrations of SNAP (50, 100, 200, 300, 400 µmol/L) cause the appearance of the maximum peak current (*ΔI*_max_), the highest at 200 µmol/L, with its subsequent decrease. K^+^ currents not suppressed. $K_{\text{in}}^{+}$/$K_{\text{out}}^{+}$ environment. (b) In ${\text{Cs}}_{\text{in}}^{+}$/${\text{Cs}}_{\text{out}}^{+}$ medium (K^+^ currents suppressed) SNAP at a concentration of 200 µmol/L cause the appearance of *ΔI*_max_, but the effect develops about twice as long as in $K_{\text{in}}^{+}$/$K_{\text{out}}^{+}$ medium at of the same NO donor concentration

At a holding potential of 45 mV, membrane currents recorded in the $K_{\text{in}}^{+}$/$K_{\text{out}}^{+}$ medium were +0.198 ± 0.006 nA, *n* = 125 (see also Figure 3a). A similar value was obtained at the level of −45 mV, equal to +0.196 ± 0.007 nA (*p* > 0.05) based on the analysis of the *I*/*V* relation of *I*_L_, whereby the maximal value of the stretch‐deactivated inwardly rectifying potassium current (*ΔI*_K1_) (Dyachenko, Husse, et al., 2009), was equal to +0.212 ± 0.006 nA, *n* = 127 (*p* > 0.05) at *V*_0_ = −74.3 ± 0.4 mV (*n* = 127).

When registering the time course in ${\text{Cs}}_{\text{in}}^{+}$/${\text{Cs}}_{\text{out}}^{+}$ medium (Figure 3b) in the control experiments, *I*_L,ns_ at *V*_h_ = −45 mV was −0.003 ± 0.001 nA, *n* = 35, and based on the *I*/*V* relationship for *I*_L_ we had −0.004 ± 0.002 nA, *n* = 11 (*p* > 0.05), and *V*_0_ = −39.6 ± 1.44 mV.

The changes in the time course of the net membrane currents at *V*_h_ = −45 mV under the action of different concentrations of SNAP, recorded in $K_{\text{in}}^{+}$/$K_{\text{out}}^{+}$ and the currents recorded in ${\text{Cs}}_{\text{in}}^{+}$/${\text{Cs}}_{\text{out}}^{+}$ medium are shown in Table 3 and Figure 3. It was shown that in $K_{\text{in}}^{+}$/$K_{\text{out}}^{+}$ medium, the time of maximal development of the SNAP effect (*t*_max_) at concentrations of 50, 100, and 200 µmol/L is similar. When the concentration of SNAP increased, the maximal peak (*ΔI*_max_) of the net currents shifted to more negative values. With a further increase in the concentration of SNAP to 300 and 400 µmol/l, *t*_max_ decreased, and *ΔI*_max_ also decreased (Table 3; Figure 3a). In all cases, after reaching *ΔI*_max_, the SNAP‐induced net currents began to decrease and reached steady‐state level after 11–15 min (*t*_s‐s_). The most pronounced changes in *ΔI*_max_ occurred when we used SNAP at a concentration of 200 µmol/L. In this case, net currents not only shifted to more negative values but also obtained negative characteristics (Table 3 and Figure 3a). Therefore, it seems that registered net currents are inwardly rectifying potassium current deactivated by SNAP (*ΔI*_K1_) and inward current through stretch‐activated nonselective cation channels (*I*_L,ns_).

**TABLE 3:** Effect of different concentrations of SNAP in $K_{\text{in}}^{+}/K_{\text{out}}^{+}$ and ${\text{Cs}}_{\text{in}}^{+}$/${\text{Cs}}_{\text{out}}^{+}$ environments on net membrane current and SNAP‐induced differential current (*ΔI*_max_) by means of online records (time‐course) at *V*_h_ = −45 mV. Mean ± SD, *n* = number of experiments, m = number of animals. For all values of *ΔI*_max_ and *ΔI*_s‐s_, *p* < 0.01 and not shown

$K_{\text{in}}^{+}/K_{\text{out}}^{+}$ (ionic composition)						${\text{Cs}}_{\text{in}}^{+}/{\text{Cs}}_{\text{out}}^{+}$ (ionic composition)	
Parameters	SNAP, (µmol/L)					Parameters	SNAP (µmol/L)
	50	100	200	300	400		200
*n*	6	10	26	11	6	*n*	15
*m*	3	4	6	3	3	*m*	5
*t*_max_, (min)	7.6 ± 0.7	7.0 ± 0.7	7.8 ± 0.4	6.4 ± 0.4	5.2 ± 0.1	*t*_max_, (min)	12.5 ± 1.0
*ΔI*_max_, (nA)	0.071 ± 0.01	0.124 ± 0.02	0.156 ± 0.01	0.090 ± 0.01	0.054 ± 0.01	*ΔI*_max_, (nA)	0.108 ± 0.01
*t*_s‐s_, (min)	15.9 ± 0.6	15.2 ± 1.6	13.6 ± 1.1	13.0 ± 0.9	10.6 ± 0.6	*t*_s‐s_, (min)	25.1 ± 1.8
*ΔI*_s‐s_, (nA)	0.051 ± 0.003	0.046 ± 0.01	0.085 ± 0.01	0.074 ± 0.006	0.078 ± 0.01	*ΔI*_s‐s_, (nA)	0.055 ± 0.008

To separate the effects on *I*_L,ns_, we induced suppression of the inwardly rectifying K^+^ currents by substituting K^+^ for extracellular Cs^+^, while the outwardly rectifying K^+^ currents were reduced by replacing K^+^ with Cs^+^ in the intracellular solution.

In a ${\text{Cs}}_{\text{in}}^{+}$/${\text{Cs}}_{\text{out}}^{+}$ medium at a SNAP concentration of 200 µmol/L, during the time course recording at the level of −45 mV, a net SNAP‐induced inward current appears and maximal value of this inward current compared to the initial values *ΔI_max_* appears after 12.5 ± 1.0 min; the effect develops approximately twice as long as in $K_{\text{in}}^{+}$/$K_{\text{out}}^{+}$ medium at the same concentration of NO donor. During this period, the SNAP‐induced inward current *ΔI*_max_ becomes equal to 0.108 ± 0.01 nA (Table 3 and Figure 3b). Recall that at the same concentration of SNAP in $K_{\text{in}}^{+}$/$K_{\text{out}}^{+}$ medium, *ΔI*_max_ was 0.156 ± 0.01 nA, and *t*_max_ was 7.8 ± 0.4 min (see Figure 3b and Table 3).

### 3.4 NO‐induced changes in the voltage dependence of *I*_L_ recorded in $K_{\text{in}}^{+}$/$K_{\text{out}}^{+}$ medium

**FIGURE 4:**
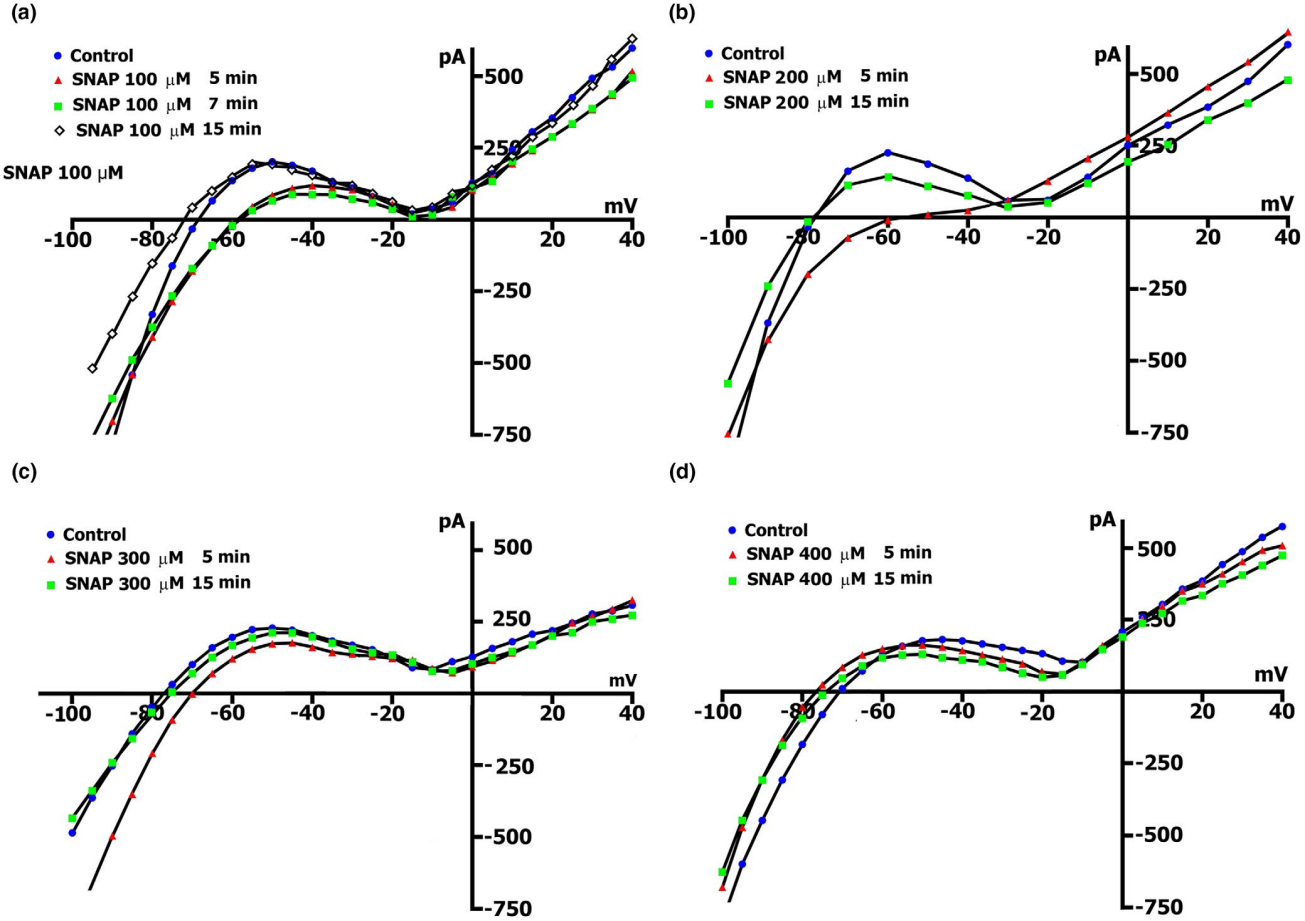
SNAP changes the voltage dependence of *I*_L_ in a $K_{\text{in}}^{+}$/$K_{\text{out}}^{+}$ environment. (a, b, c) At a concentration of 100, 200, 300 µmol/L in the first 5 min, a reduction of the positive hump of the *I*/*V* curve (at −55 to −60 mV) is noted, an increase in the amplitude of negative current (at −80 mV) and value of depolarization (change of the zero current potential *V*_0_, ie intercept of *I*/*V* curve with current axis). After 15 min, the positive hump of the *I*/*V* curve approaches the initial value, the membrane hyperpolarizes, and the emerging negative current (at −80 mV) was inhibited. (d) At a concentration of 400 µmol/L in the first 5 min, the negative current (at −80 mV) was inhibited and a shift of *E*_0_ to the negative region was observed. Legend: (a) control: circles, perfusion of SNAP 5 min: triangles, 10 min: squares, 15 min: rhombus. (b), (c), (d) control: circles, perfusion of SNAP 5 min: triangles, 15 min: squares

The voltage dependence of *I*_L_ and its modulation by different concentrations of SNAP is shown on the *I*/*V* curves in Figure 4 and Table 4. Before SNAP (circles), the *I*/*V* curve was *N*‐shaped and crossed the voltage axis (zero current potential *V*_0_) at −75 to −80 mV (equivalent to resting potential of nonclamped cell, *V*_0_ = −74.3 ± 0.4 mV, *n* = 127). The modest concentration of SNAP of 100 or 200 µmol/L in the first 5 min shifted the net currents to more negative values (Figure 4a and b, triangles compared to circles, Table 4), and *V*_0_ changed toward depolarization. The differential current at −45 mV (^5/C^ *ΔI*_SNAP (−45)_) at the mentioned concentrations was (−) 0.084 ± 0.01 and (−) 0.107 ± 0.01 nA, respectively, (the differential current *ΔI*_SNAP_ that occurs when the *I*_L_ values are shifted to a more negative direction relative to the reference values, is indicated by a minus "−", and the differential current *ΔI*_SNAP_ that occurs when the values of *I*_L_ are shifted to a more positive direction is denoted by a plus "+"). The late currents *I*_L_ increased at a negative potential of −80 mV, and at a concentration of SNAP of 100 and 200 µmol/L, ^5/C^ *ΔI*_SNAP_ and they were (−) 0.072 ± 0.01 and (−) 0.122 ± 0.03 nA, respectively. The SNAP‐induced changes at these concentrations in the late currents (*ΔI*_SNAP_) followed an outwardly rectifying voltage‐dependence with a reversal potential (*E*_rev_) of −30 mV (Figure 4a,b). The changes suggest that SNAP modulates not a single but several ionic current components (see below). After 7 min, the ^7/C^ *ΔI*_SNAP_ values at both −45 and −80 mV levels were slightly changed (Table 4). Also, the values of *V_0_* remain at a similar level (Table 5). However, after 10 min at a concentration of SNAP of 100 µmol/L and 200 µmol/L, *^10^* ^/^ *^C^ΔI_SNAP_* values at −45 mV decrease. More importantly, the late currents *I*_L_ were reduced at negative potentials of −80 mV, and at concentrations of SNAP of 100 and 200 µmol/L, ^10/C^ *ΔI*_SNAP_ were (+) 0.053 ± 0.01 and (+) 0.083 ± 0.02 nA (Table 4; Figure 4b). The (+) sign demonstrates that after 10 min, SNAP induces inhibition even on the background *I*_L,ns_. The *V*_0_ values begin to shift toward hyperpolarization, to the original values (Table 5). After 15 min, *V*_0_ registrations not only slightly differed from the initial ones, but also exceeded them (Table 5), and ^15/C^ *ΔI*_SNAP_ at a level of −45 mV, was close to the original values (Figure 4a,b and Table 4). The same happened at the level of −80 mV, where inhibition of the background *I*_L,ns_ was observed.

**TABLE 4:** The amplitude of SNAP‐induced differential current *ΔI*_SNAP_ described from *I*/*V*‐curves (*I*_L_) at −45 and −80 mV at different concentrations of SNAP after 5, 7, 10, 15 min perfusion. $K_{\text{in}}^{+}$/$K_{\text{out}}^{+}$ solutions. Mean ± SD, *n *= number of experiments, m = number of animals. *I* (nA) ‐ measured value of current. The differential current *ΔI*_SNAP_ that occurs when the values of *I*_L_ are shifted to a more negative direction relative to the reference values is indicated by a minus (−), and the differential current when the values of *I*_L_ are shifted to a more positive direction is denoted by a plus (+). In all cases, *p* < 0.01

SNAP, (µmol/L)	*V*, (mV)	*n*	*m*	Control	5 min perfusion		7 min perfusion		10 min perfusion		15 min perfusion	
				*I*_L_ (nA)	^5^ *I*_L,SNAP_ (nA)	^5/C^ *ΔI*_SNAP_ (nA)	^7^ *I*_L,SNAP_ (nA)	^7/C^ *ΔI*_SNAP_ (nA)	^10^ *I*_L,SNAP_ (nA)	^10/C^ *ΔI*_SNAP_ (nA)	^15^ *I*_L,SNAP_ (nA)	^15/C^ *ΔI*_SNAP_ (nA)
100	−45	11	3	+0.180 ± 0.01	+0.106 ± 0.02	(−)0.084 ± 0.01	+0.104 ± 0.02	(−)0.079 ± 0.02	+0.102 ± 0.01	(−)0.053 ± 0.01	+0.100 ± 0.01	(−)0.050 ± 0.01
	−80	19	5	−0.316 ± 0.01	−0.408 ± 0.02	(−)0.072 ± 0.01	−0.346 ± 0.03	(−)0.064 ± 0.01	−0.316 ± 0.03	(+)0.053 ± 0.01	−0.310 ± 0.03	(+)0.052 ± 0.01
200	−45	26	6	+0.179 ± 0.01	+0.065 ± 0.01	(−)0.107 ± 0.01	+0.038 ± 0.01	(−)0.126 ± 0.01	+0.104 ± 0.02	(−)0.064 ± 0.01	+0.101 ± 0.02	(−)0.062 ± 0.01
	−80	19	4	−0.211 ± 0.01	−0.344 ± 0.03	(−)0.122 ± 0.03	−0.325 ± 0.04	(−)0.139 ± 0.03	−0.199 ± 0.02	(+)0.083 ± 0.02	−0.190 ± 0.02	(+)0.080 ± 0.02
300	−45	10	3	+0.126 ± 0.01	+0.083 ± 0.02	(−)0.052 ± 0.01	+0.091 ± 0.03	(−)0.061 ± 0.01	+0.094 ± 0.02	(−)0.024 ± 0.01	+0.102 ± 0.02	(−)0.020 ± 0.01
	−80	7	3	−0.231 ± 0.07	−0.431 ± 0.02	(−)0.257 ± 0.04	−0.328 ± 0.03	(−)0.162 ± 0.02	−0.210 ± 0.04	(+)0.075 ± 0.03	−0.215 ± 0.04	(+)0.071 ± 0.03
400	−45	6	3	+0.134 ± 0.03	+0.115 ± 0.02	(−)0.019 ± 0.006	+0.063 ± 0.01	(−)0.053 ± 0.02	+0.056 ± 0.01	(−)0.082 ± 0.03	+0.050 ± 0.01	(−)0.072 ± 0.03
	−80	6	3	−0.188 ± 0.04	−0.138 ± 0.01	(+)0.072 ± 0.04	−0.120 ± 0.03	(+)0.062 ± 0.02	−0.115 ± 0.02	(+)0.053 ± 0.01	−0.111 ± 0.02	(+)0.049 ± 0.01

**TABLE 5:** Zero‐current potential (*E*_0_) ‐ The intercept of the *I*/*V* curves with the zero‐current axis before and during perfusion of SNAP in different concentrations within 15 min. K^+^ currents unblocked $K_{\text{in}}^{+}/K_{\text{out}}^{+}$ solutions. Mean ± SD, *n* = number of experiments, m = number of animals. A *p* > 0.05 was considered to indicate a statistically nonsignificant difference (*P* = NS). All other instances with *p* < 0.01 are not indicated. ^#^ *p* = NS versus 7 min perfusion, *p = NS versus stretch 15 min perfusion

SNAP (µmol/L)	*n*	*m*	Control	5 min perfusion	7 min perfusion	10 min perfusion	15 min perfusion
			*V*_0_, (mV)	*V*_0_, (mV)	*V*_0_, (mV)	*V*_0_, (mV)	*V*_0_, (mV)
100	11	4	−71.4 ± 0.8	−59.4 ± 4.0^#^	−62.3 ± 2.0	−68.9 ± 1.5	−77.5 ± 2.3
200	46	10	−72.5 ± 0.9	−55.3 ± 3.4^#^	−57.6 ± 3.3	−62.6 ± 3.3	−75.0 ± 3.3
300	10	4	−78.6 ± 0.8	−63.7 ± 4.5^#^	−65.6 ± 2.8	−72.6 ± 2.5	−80.6 ± 3.9
400	6	3	−67.6 ± 1.9	−78.8 ± 2.7^#^	−78.3 ± 4.7	−75.3 ± 3.7*	−75.4 ± 1.3

Thus, we observed a biphasic effect—reduction with a subsequent return to close to original values of the positive hump of *I*/*V* curve (at −55 to −60 mV) and value of depolarization (change of the zero current potential *V*_0_,) followed by hyperpolarization. Therefore, it seems that in the absence of stretch, NO donor SNAP in concentrations of 100 or 200 µmol/L, first causes deactivation of the stretch‐deactivated inwardly rectifying potassium current (*ΔI*_K1_), followed by its elimination. In this term, it is even more important that released NO causes *I*_L,ns_ activation followed by membrane depolarization and then *I*_L,ns_ inhibition followed by membrane hyperpolarization.

A further increase in the SNAP concentration to 300 µmol/L after 5 min produced similar changes (especially in the *I*_L,ns_ portion) that returned completely to baseline values after 15 min from the beginning of the application (Figure 4c and Table 4). *ΔI*_SNAP_ was not changed in the presence of 400 µmol/L SNAP, at the level of −45 mV, which is associated with a reduction in the positive hump of the *I*/*V* curve. At the same time, at the level of −80 mV, inhibition of *I*_L,ns_ was observed already after the 5th min, which remain without additional changes throughout the whole period of registration (Figure 4d and Table 4). Unlike other SNAP concentrations, at 400 µmol/L *V*_0_ does not shift toward depolarization, but toward hyperpolarization and remains at this level throughout the whole recording period (Table 5). In general, a high concentration of NO causes inhibition of the inward current through stretch‐activated nonselective cation channels (*I*_L,ns_), which leads to membrane hyperpolarization.

**FIGURE 5:**
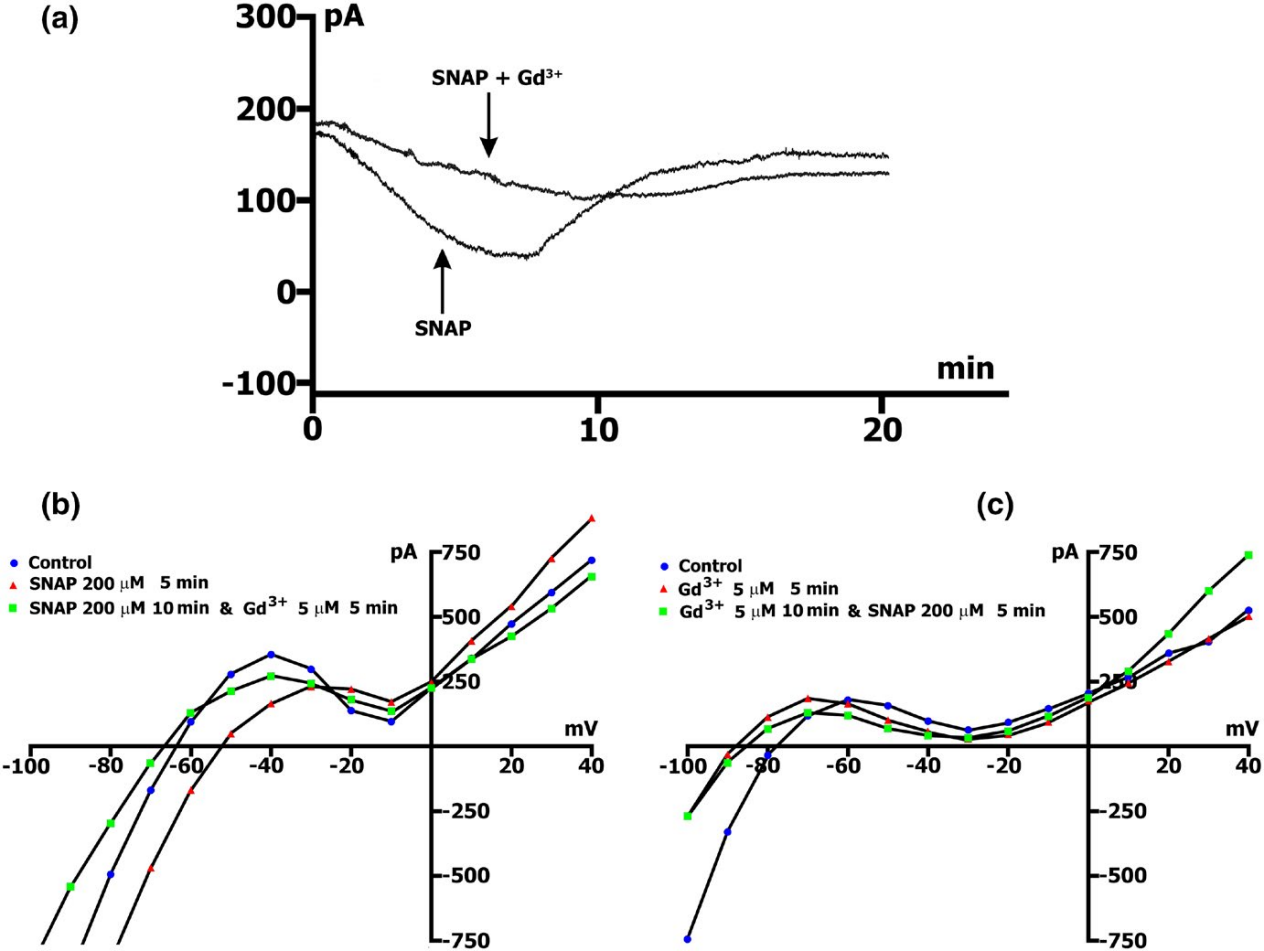
Gadolinium reduces SNAP‐induced net currents. SNAP at the concentration of 200 µmol/L. K^+^ currents were not suppressed in $K_{\text{in}}^{+}$/$K_{\text{out}}^{+}$ environment. (a) Online records (time course) of membrane current, *V*_m_ clamped to a holding potential of −45 mV. Gd^3+^ (5 µmol/L) in the presence of 200 µmol/L SNAP reduces SNAP‐induced net currents and maximum peak current (*ΔI*_max_). (b) Gd^3+^ blocks *I*_SNAP_. Legend ‐ control: circles, perfusion of SNAP 5 min: triangles, perfusion of SNAP 10 min, and Gd^3+^ 5 min: squares. (c) *I*_SNAP_ does not develop on the background of the preliminary administration of Gd^3+^. Legend ‐ control: Circles, perfusion of Gd^3+^ 5 min: triangles, perfusion of Gd^3+^ 10 min and SNAP 5 min: squares

When registering the time course in $K_{\text{in}}^{+}$/$K_{\text{out}}^{+}$ medium with simultaneous perfusion with SNAP at a concentration of 200 µmol/L and Gd^3+^ at a concentration of 5 µmol/L (Figure 5a and Table 6), the maximal peak deviation of the total current from the control values was *ΔI*_max_= 0.067 ± 0.009 nA, and appears 10.5 ± 0.7 min after injection of the compounds (recall that SNAP at the same concentration without Gd^3+^ causes *ΔI*_max_= 0.156 ± 0.01 nA in 7.8 ± 0.4 min).

**TABLE 6:** Effect of the application of 5 µmol/L Gd^3+^ and 200 µmol/L SNAP in $K_{\text{in}}^{+}$/$K_{\text{out}}^{+}$ and ${\text{Cs}}_{\text{in}}^{+}$/${\text{Cs}}_{\text{out}}^{+}$ environments on net membrane current and SNAP‐induced differential current (*ΔI*_max_) by means of online records (time‐course) at *V*_h_ = −45 mV. Mean ± SD, *n* = number of experiments, *m* = number of animals

Parameters	$K_{\text{in}}^{+}/K_{\text{out}}^{+}$ & Gd^3+^	${\text{Cs}}_{\text{in}}^{+}$/${\text{Cs}}_{\text{out}}^{+}$ & Gd^3+^
*n*	11	6
*m*	4	3
*t*_max_, (min)	10.5 ± 0.7	15.0 ± 2.3
*ΔI*_max_, (nA)	0.067 ± 0.009	0.040 ± 0.01
*t*_s‐s_, (min)	16.8 ± 0.5	27.2 ± 1.0
*ΔI*_s‐s_, (nA)	0.053 ± 0.01	0.043 ± 0.01

The voltage dependence of *I*_L,ns_, its modulation by 200 µmol/L SNAP and Gd^3+^ sensitivity are shown on the *I*/*V* curves in Figure 5b. SNAP, as shown above, depolarizes the cell by approximately 20 mV from −72.5 ± 0.9 to −55.3 ± 3.4 (*n* = 46), actually in the first 5 min it reduced the positive hump of the *I*/*V* curve, ^5/C^ *ΔI*_SNAP_ = (−) 0.107 ± 0.01 (*n* = 26) and markedly increased *I*_L,ns_ at −80 mV, ^5/C^ *ΔI*_SNAP_ = (−) 0.122 ± 0.03 (*n* = 19) (Figure 5b triangles compared to circles). Subsequent introduction of 5 µmol/L Gd^3+^ into the medium causes membrane hyperpolarization (*V*_0_ = −76.8 ± 0.1, *n* = 9), decreased *ΔI*_SNAP, Gd_ at −45 mV to (−) 0.050 ± 0.01, (*n* = 9) and inhibited *ΔI*_SNAP, Gd_ at −80 mV to (+) 0.196 ± 0.02, (*n* = 9) (Figure 5b, squares compared to circles). Based on all above, it seems that Gd^3+^ caused SNAP‐induced *I*_L,ns_ inhibition in the same manner as stretch‐induced *I*_L,ns_.

In cases when we start with 5 µmol/L Gd^3+^, after the 5th min from the beginning of the application, the positive hump of the *I*/*V* curve shifts to more negative potentials −73 ± 2 mV, (*n* = 6) (compare −55 to −60 mV in the control), but the amplitude of positive hump does not change (*p* > 0.05), and hyperpolarization was observed, at which *V*_0_ was equal to −92 ± 4.6 mV, *n* = 6 (compare to *V*_0_ in the control, equal to −74.3 ± 0.4 mV, *n* = 127). At the same time, there was a pronounced inhibition of *I*_L,ns_ at the level of −80 mV. In this case, ^5/C^ *ΔI*_Gd_ was (+) 0.176 ± 0.02 nA (*n* = 6). Subsequent injection of 200 µmol/l SNAP into PSS did not lead to significant changes in *I*_L_, (*p* > 0.05) (Figure 5c, squares compared to circles).

In our case, SNAP‐induced current *I*_L,ns_ was described as a product of driving force (*V* − *E_ns_*) with a voltage‐independent conductance *G*_ns_. According to literature, the reversal potential of *E*_ns_ =−10 mV, voltage‐independent conductance, and blockage of *G*_ns_ by 5 µmol/L Gd^3+^, all together suggested that the stretch‐activated and SNAP‐induced *I*_L,ns_ are the same nonselective cation current (Hu & Sachs, 1997; Kamkin et al., 2003). In addition, the definition of *I*_L,ns_ by its block with Gd^3+^ is questionable because Gd^3+^ interferes with the *Ca* ^*2+*^ ‐ and *K* ^*+*^ ‐ currents (Belus & White, 2002; Hongo et al., 1997).

### 3.5 NO‐induced changes in the voltage dependence of *I*_L_ recorded in ${\text{Cs}}_{\text{in}}^{+}$/${\text{Cs}}_{\text{out}}^{+}$ medium

**FIGURE 6:**
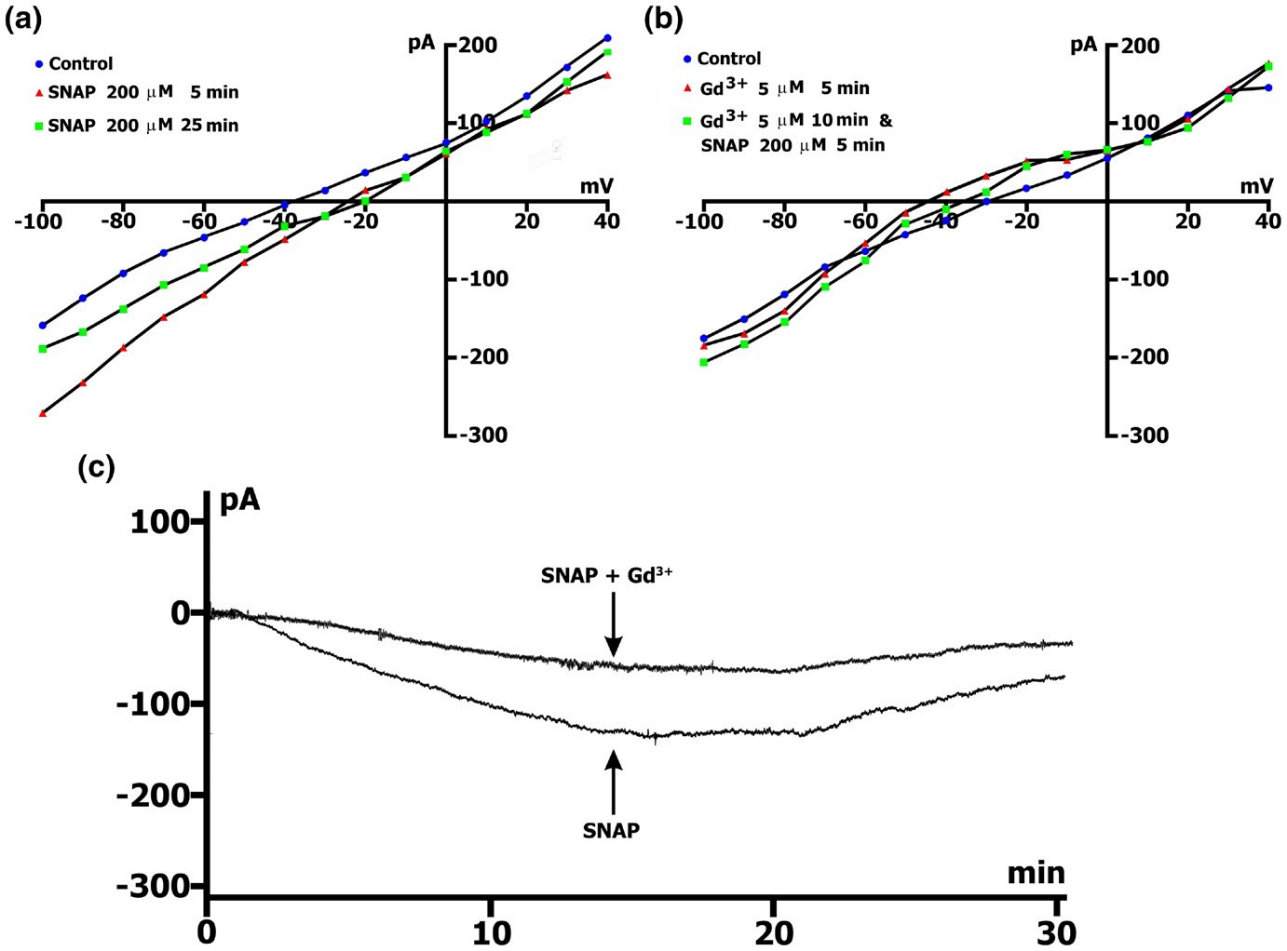
Gadolinium reduces SNAP‐induced current (*I*_ns_). SNAP at the concentration of 200 µmol/L. K^+^ currents suppressed. ${\text{Cs}}_{\text{in}}^{+}$/${\text{Cs}}_{\text{out}}^{+}$ environment. (a) SNAP changes voltage dependence *I*_L_. In the first 5 min, there was an increase in the value of negative current (*I*_ns_) at −45 and −80 mV (triangles vs circles in control) and value of depolarization (change of the zero current potential *V*_0_, ie intercept of *I*/*V* curve with current axis). After 25 min, the *I*_ns_ value decreases (squares vs circles in control). (b) *I*_SNAP_ does not develop on the background of preliminary administration of 5 µmol/L Gd^3+^. Legend ‐ control: circles, perfusion of Gd^3+^ 5 min: triangles, perfusion of Gd^3+^ 10 min and SNAP 5 min: squares. (c) Online records (time course) of membrane current, *V*_m_ clamped to a holding potential of −45 mV. Gd^3+^ (5 µmol/L) in the presence of 200 µmol/L SNAP reduces SNAP‐induced net currents and maximum peak current (*ΔI*_max_)

Table 7 demonstrates the *I*_L_ changes as a function of recording time of the *I*/*V* curve in the presence of 200 µmol/L SNAP in ${\text{Cs}}_{\text{in}}^{+}$/${\text{Cs}}_{\text{out}}^{+}$ solutions, while Figure 6. A shows an example of the control curve and its changes after 5 and 25 min of recording. It was shown that after 5 min of perfusion (triangles compared to circles), the inward current *I*_L,ns,SNAP,(−45)_ was increased, whereby the resulting differential current ^5/C^ *ΔI*_SNAP (−45)_ was equal to (−) 0.080 ± 0.01 nA. Furthermore, until the 10th min, *ΔI*_SNAP (−45)_ increased slightly and did not change until the 15th min, so that ^10/C^ *ΔI*_SNAP (−45)_ practically did not differ from ^15/C^ *ΔI*_SNAP(−45)_. Later, ^20/C^ *ΔI*_SNAP(−45)_ and ^25/C^ *ΔI*_SNAP(−45)_ decreased and were close to each other (Table 7). *I_SNAP(_*_−_ *_80)_* also increased significantly in the first 5 min, with ^5/C^ *ΔI*_SNAP (−80)_ becoming equal to (−) 0.156 ± 0.04 nA. Furthermore, after 10 min from the beginning of perfusion, *I*_SNAP(−80)_ decreased. In the 15th, 20th, and 25th min, *ΔI*_SNAP(−80)_ continued to decrease (Table 7). In general, we first observed activation followed by inhibition of the *I*_L,ns_, although not to the initial values. Typically, *V*_0_ shifts to depolarization in the first 5 min and remains stable throughout the whole recording period (Table 8). The SNAP‐induced *I*_L,ns_ first increases and then decreases, without reaching the initial values.

**TABLE 7:** The amplitude of SNAP‐induced differential current *ΔI*_SNAP_ described from *I*/*V*‐curves (*I*_L_) at −45 and −80 mV at 200 µmol/L of SNAP after 5, 10, 15, 20, 25 min of perfusion ${\text{Cs}}_{\text{in}}^{+}$/${\text{Cs}}_{\text{out}}^{+}$ solutions. Mean ± SD, *n *= number of experiments, *m* = number of animals. *I* (nA) − measured value of current. The differential current *ΔI*_SNAP_ (nA) that occurs when the values of *I*_L_ are shifted to a more negative direction relative to the reference values, is indicated by a minus (−). ^†^ *p *< 0.001 versus control, *p = NS versus ^5/C^ *ΔI*_SNAP_, **p = NS versus ^10/C^ *ΔI*_SNAP_, all other instances with *p* < 0.01 are not indicated

SNAP	*V*, (mV)	*n*	*m*	Control	5 min perfusion		10 min perfusion		15 min perfusion	
				*I*_L_ (nA)	^5^ *I*_L,SNAP_ (nA)	^5/C^ *ΔI*_SNAP_ (nA)	^10^ *I*_L,SNAP_ (nA)	^10/C^ *ΔI*_SNAP_ (nA)	^15^ *I*_L,SNAP_ (nA)	^15/C^ *ΔI*_SNAP_ (nA)
200 (µmol/L)	−45	21	7	−0.004 ± 0.002	−0.069 ± 0.01^†^	(−)0.080 ± 0.01	−0.077 ± 0.01	(−)0.087 ± 0.01*	−0.072 ± 0.01	(−)0.085 ± 0.02**
	−80	19	6	−0.06 ± 0.01	−0.203 ± 0.01^†^	(−)0.156 ± 0.04	−179 ± 0.02	(−)0.136 ± 0.01*	−0.155 ± 0.01	(−)0.132 ± 0.03**

SNAP	*V*, (mV)	*n*	*m*	20 min perfusion		25 min perfusion	
				^20^ *I*_L,SNAP_ (nA)	^20/C^ *ΔI*_SNAP_ (nA)	^25^ *I*_L,SNAP_ (nA)	^25/C^ *ΔI*_SNAP_ (nA)
200 (µmol/L)	−45	21	7	−0.060 ± 0.007	(−)0.055 ± 0.01	−0.054 ± 0.01	(−)0.054 ± 0.01
	−80	19	6	−0.159 ± 0.02	(−)0.108 ± 0.02	−0.143 ± 0.01	(−)0.084 ± 0.01

**TABLE 8:** Zero‐current potential (*E*_0_) ‐ The intercept of the *I*/*V* curves with the zero‐current axis before and during the perfusion of 200 µmol/L of SNAP within 25 min. K^+^ currents blocked, ${\text{Cs}}_{\text{in}}^{+}/{\text{Cs}}_{\text{out}}^{+}$ solutions. *V*_0_ ‐ Measured value of potential. Mean ± SD, *n* = number of experiments, *m* = number of animals

SNAP, (µmol/L)	*n*	*m*	Control	5 min perfusion	10 min perfusion	15 min perfusion	20 min perfusion	25 min perfusion
			*V*_0_, (mV)	*V*_0_, (mV)	*V*_0_, (mV)	*V*_0_, (mV)	*V*_0_, (mV)	*V*_0_, (mV)
200	14	5	−35.4 ± 2.2	−20.4 ± 1.3	−20.3 ± 3.9	−18.7 ± 3.9	−17.4 ± 3.1	−16.4 ± 2.8

Initially administered Gd^3+^ at a concentration of 5 µmol/l induced membrane hyperpolarization from −35.4 ± 2.2 to −47 ± 2.8 mV, but has little effect on the *I*_L_ and initial *I*_L,ns_ (*p* > 0.05). Subsequent administration of 200 µmol/L SNAP in the medium did not cause significant changes in *I*_L_ (Figure 6b).

These data correlate to the data obtained in the current recording experiments, while in conditions of time course recording, *V*_h_ was equal to −45 mV (Figure 6c). The introduction of 5 µmol/L Gd^3+^ into the ${\text{Cs}}_{\text{in}}^{+}$/${\text{Cs}}_{\text{out}}^{+}$ medium, simultaneously with 200 µmol/L SNAP (Table 6), caused a highly significant reduction (*p* < 0.005) in the SNAP‐induced current at the level of −45 mV, of approximately 2.7 times (Table 3 compared to Table 6). Thus, in a ${\text{Cs}}_{\text{in}}^{+}$/${\text{Cs}}_{\text{out}}^{+}$ environment, Gd^3+^ prevents the development of SNAP‐induced currents, which is related to the fact that SNAP‐induced *I*_L,ns_ is the well‐known stretch‐activated *I*_L,ns_.

### 3.6 NO abolishes stretch‐induced net inward currents recorded in $K_{\text{in}}^{+}$/$K_{\text{out}}^{+}$ medium; (time course and voltage dependence)

On the one hand, online recordings during a stretch in the presence of potassium showed the occurrence of a net inward current, which increases with an increasing stretch extent. During the continuous stretch, the inward current remained constant. Gd^3+^ abolishes this effect (see Section 3.1, Figure 1a,b). The stretch also shifted the *I*/*V* relation to more negative currents and depolarized *V*_0_. The obtained net currents (Figure 1c,d, and e) indicated that the stretch attenuates the hump of the outward current and causes slope reduction between −80 and −100 mV. On the other hand, for instance, at a concentration of SNAP of 200 µmol/L, we recorded net currents induced by SNAP at −45 mV, which first increased and then decreased. These net currents are SNAP‐deactivated, inwardly rectifying potassium current (*ΔI*_K1_) and inward current through stretch‐activated non‐selective cation channels (*I*_L,ns_), which are both blocked by Gd^3+^ (see Sections 3.3 and 3.4; Figures 3, 4 and 5).

**FIGURE 7:**
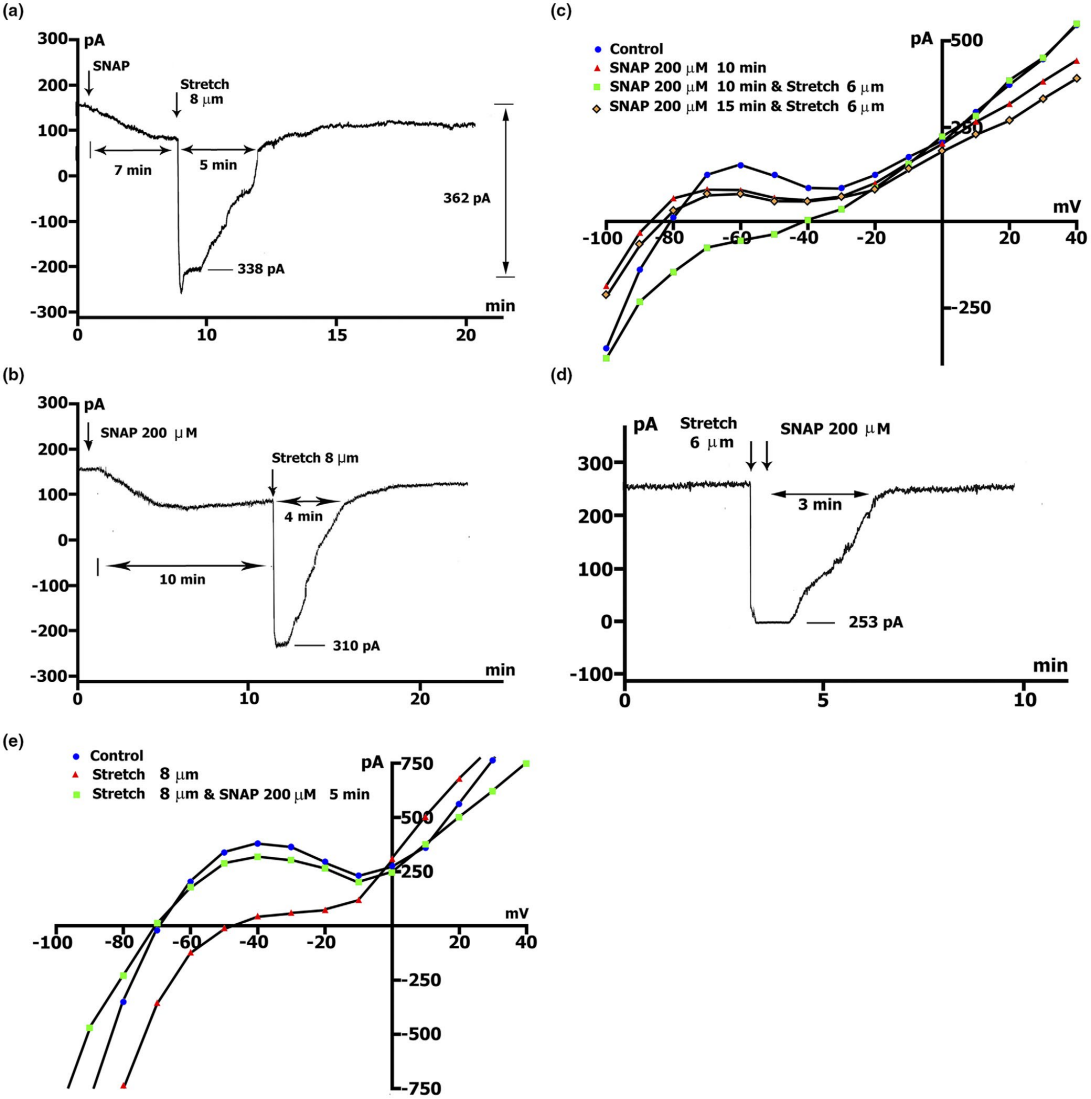
SNAP at the concentration of 200 µmol/L abolishes stretch‐induced net inwards currents in $K_{\text{in}}^{+}$/$K_{\text{out}}^{+}$ environment: Time‐course and voltage dependence. *V*_m_ clamped to a holding potential of −45 mV. (a) Online records (time course) of membrane current. Stretch was applied at the level of *ΔI*_max_ during perfusion with SNAP. Value of stretch (8 µm) and amount of stretch‐induced inward current at −45 mV, indicated. (b) The same as in A, but the stretch was applied at *ΔI*_s‐s_. (c) Voltage dependence of *I*_L_ in control (circles), after 10 min perfusion of SNAP (triangles), after 10 min perfusion of SNAP on the background of stretch by 6 µm (squares), after another 5 min of continued stretching (rhombus). (d) Time course of membrane current. SNAP was applied after stretching the cell by 6 µm. (e) Voltage dependence of *I*_L_ in control (circles), *I*_L_ after stretching by 8 µm (triangles), after 5 min of a continued stretch at the level of perfusion of SNAP (squares)

In this study, we demonstrated the elimination of the stretch‐induced net currents by initial injection of 200 µmol/L SNAP in a $K_{\text{in}}^{+}$/$K_{\text{out}}^{+}$ environment. Online recording (Figure 7a) demonstrates appearance of a stretch‐induced current of −0.338 nA (0.392 ± 0.03 nA, *n* = 7 vs. −0.441 ± 0.017 nA in control, *n* = 21) when cells were stretched by 8 µm on the background of the net currents induced by SNAP, (*ΔI*_max_ through *t_max_* equal to 7.8 ± 0.4 min). This current persists for no more than 2 min, and spontaneously disappears within 3 min, despite the still existing cell elongation. At the same time, the dynamics of development of the effect of SNAP, remain.

A similar effect was caused by applying an 8 µm stretch at the level of *t*_s‐s_ = 13.6 ± 1.1 min, on the background of *ΔI*_s‐s_. The 8 µm stretch, caused a stretch‐induced current of −0.310 nA (−0.398 ± 0.012 nA, *n* = 6) (see Table 1, row B), which lasts no more than 1.5 min, and spontaneously disappears in max 2.5 min, despite the presence of cell stretch. The dynamic of the development of the effect of SNAP was preserved (Figure 7b). Consequently, regardless of the extent of development of the SNAP‐induced net currents, the cell stretch by a certain amount causes stretch‐induced current at −45 mV, with a value close to the value of SNAP‐induced current (from time course: −0.441 ± 0.017 nA, *n* = 21 vs −0.398 ± 0.012 nA, *n* = 6; from the *I*/*V* curves: see Table 1). The stretch‐induced current in the presence of SNAP does not remain constant as in the absence of SNAP.

The voltage dependence of *I*_L_ and its modulation by SNAP and stretch is shown on the *I*/*V* curves in Figure 7c. After 10 min, SNAP at a concentration of 200 µmol/l, as previously reported, returns the *I*_L_ to values close to the original values, whereby ^10/C^ *ΔI*_SNAP_ at −45 and −80 mV was equal to (−) 0.054 nA, that is, (−) 0.064 ± 0.01 (*n* = 26, *p* > 0.05) and (+) 0.054 nA that is, (+) 0.083 ± 0.02 (*n* = 19, *p* > 0.05, triangles compared to circles in the control). In this case, *V*_0_ was slightly shifted to the negative values. Stretching, for example 6 µm, applied on the background of SNAP, causes appearance of the *I*_L,ns_ whose values at −45 and −80 mV were equal to (−) 0.153 nA (−0.176 ± 0.019 nA, *n* = 8) and (−) 0.180 nA (−0.290 ± 0.08 nA, *n* = 8), respectively, while *V*_0_, as expected, significantly shifts toward depolarization. However, after another 5 min, on the background of stretch, the voltage dependence of the *I*_L_ shifts to values close to the initial ones (rhombus vs. triangles, *p* > 0.05). In addition, *V*_0_ acquires the original value. Based on all the above, it seems that, on the background of SNAP, stretch leads to a characteristic cell response, which spontaneously disappears and is probably associated with an excess amount of NO.

If we apply initial stretch, for example, 6 µm (Figure 7d), the stretch‐induced net current at *V*_h_ = −45 mV, is approximately −0.253 nA (−0.195 ± 0.009 nA, *n* = 36). Subsequent injection of 200 µmol/L SNAP after only 3 min caused elimination of the stretch‐induced net current, despite the existence of continuous stretch.

The voltage dependence of *I*_L_ and its modulation by stretch and SNAP is shown on the *I*/*V* curves in Figure 7. E. Stretching, for example, 8 µm, causes a reduction in the positive hump of the *I*/*V* curve (at −55 to −60 mV) and shifts *V*_0_ toward depolarization (Figure 7e, triangles vs. circles in control). At the level of −45 and −80 mV, *ΔI* have the value of (−) 0.347 (−0.398 ± 0.012 nA, *n* = 6) and (−) 0.388 nA (−0.580 ± 0.03 nA, *n* = 6), while the subsequent introduction of 200 µmol/L SNAP returns the *I*_L_ curve to its original values in only 5 min (squares vs. circles in control).

Thus, the introduction of NO into the medium on the background of stretch, causes elimination of the *I*_SAC_, despite the ongoing elongation of the cell.

### 3.7 NO abolishes stretch‐induced net inward currents recorded in ${\text{Cs}}_{\text{in}}^{+}$/${\text{Cs}}_{\text{out}}^{+}$ medium

**FIGURE 8:**
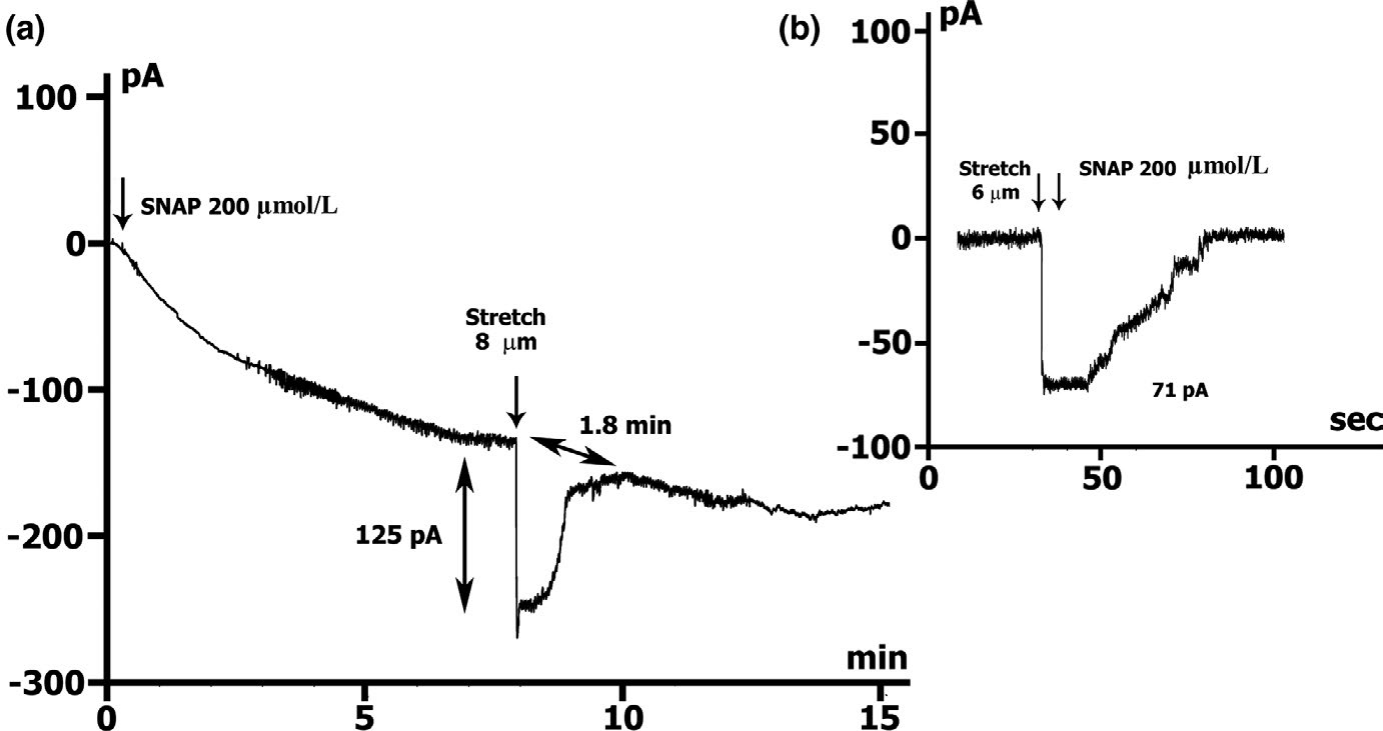
SNAP at the concentration of 200 µmol/L abolishes stretch induces *I*_SAC_ in ${\text{Cs}}_{\text{in}}^{+}$/${\text{Cs}}_{\text{out}}^{+}$ environment. K^+^ currents suppressed. (a) Online record of membrane current, *V*_m_ clamped to a holding potential of −45 mV. Stretch was applied at the level near *ΔI*_max_ during perfusion with SNAP. The value of stretch (8 µm) and the amount of stretch‐induced inward current at −45 mV are indicated. (b) Time course of membrane current. SNAP was applied after stretching the cell by 6 µm

*I*_SAC_ elimination was demonstrated with an initial injection of 200 µmol/L SNAP in a ${\text{Cs}}_{\text{in}}^{+}$/${\text{Cs}}_{\text{out}}^{+}$ medium. Figure 8. A shows the appearance of *I*_SAC(−45)_ with a value of −0.125 nA (−0.155 ± 0.024 nA in the control, *n* = 6), near *ΔI*_max_, when the cell is stretched by 8 µm on the background of SNAP‐induced current. This current persists for no more than 2 min and spontaneously disappears despite the ongoing elongation of the cell. At the same time, the dynamics of development of the effect of SNAP remain. Figure 8b shows *I*_SAC(−45)_ generated by a 6 µm stretch, which induced a current equal to −0.071 nA (−0.082 ± 0.011 nA in control, *n* = 5). The application of SNAP on the background of recorded current caused its abolition despite the ongoing elongation of the cell.

### 3.8 Participation of BAY 41‐2272 in modulation of the membrane currents *I*_L,ns,_ and *I*_K1_ recorded in $K_{\text{in}}^{+}$/$K_{\text{out}}^{+}$ medium

Since the NO donor SNAP, activates the β subunit of sGC and triggers NO‐dependent cGMP‐PKG pathway or induces S‐nitrosylation of the *SACs*, we decided to examine the involvement of the NO‐independent cGMP‐PKG pathway in the modulation of *I*_L,ns_ membrane currents. Therefore, we employed a soluble guanylate cyclase (sGC) stimulator, 3‐(4‐amino‐5‐cyclopropylpyrimidin‐2‐yl)‐1‐(2‐fluorobenzyl)‐1H‐pyrazolo [3,4‐b]pyridine, known as BAY 41‐2272, which acts on the (NO)‐independent regulatory binding site in the α1 subunit of the sGC (Becker et al., 2001; Stasch et al., 2001).

Although BAY 41‐2272 was used in experiments at a concentration of 10 µmol/L, the effect of the *EC*_50_ concentration (5 µmol/L) was additionally examined as a minimally effective concentration under our experimental conditions. In addition, significantly higher concentrations of 50 or 100 µmol/L, were considered to reveal a possible nonspecific effect of BAY41‐2272.

**FIGURE 9:**
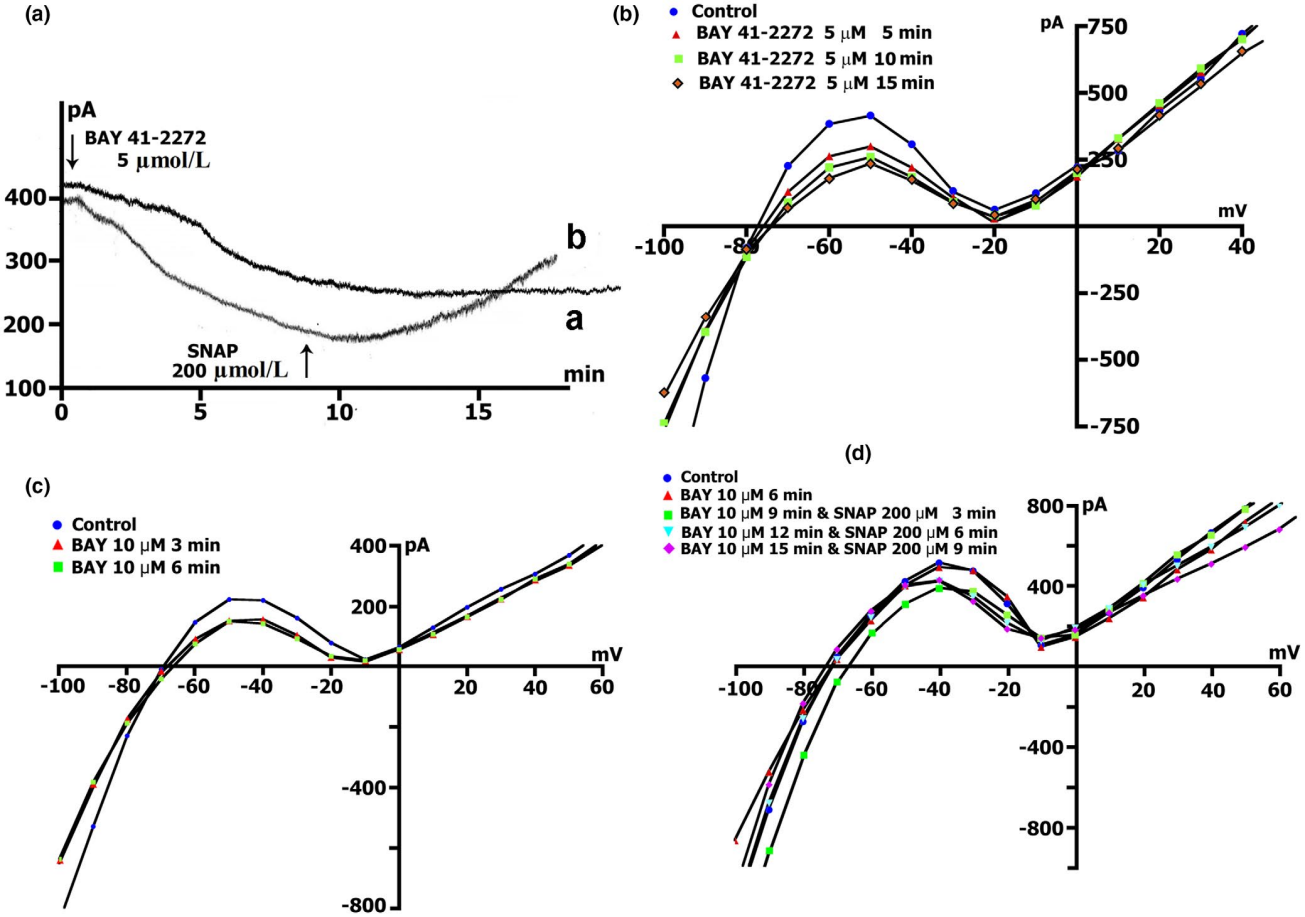
BAY41‐2272 changes the time course and voltage dependence of *I*_L_ in a $K_{\text{in}}^{+}$/$K_{\text{out}}^{+}$ environment. (a) Online records (time course) of membrane current, *V*_m_ clamped to a holding potential of −45 mV. BAY41‐2272 (5 µmol/L) shifted the net currents to more negative values (Curve ‐ a). The additional introduction of SNAP (200 µmol/l) does not lead to a further increase in the net current, but on the contrary, will decrease it with the dynamics characteristic of pure SNAP. (b) At a concentration BAY41‐2272 of 5 µmol/L in the first 5 min, a reduction of the positive hump of the *I*/*V* curve (at −55 to −60 mV) is noted, a decrease in the amplitude of negative current (at −90 mV) and an increase *V_0_* (change of the zero current potential *V*_0_, i.e., intercept of *I*/*V* curve with current axis). Legend ‐ control: circles, perfusion of BAY41‐2272 5 min: triangles, perfusion of BAY41‐2272 10 min: squares, perfusion of BAY41‐2272 15 min: rhombuses. (c) At a concentration BAY41‐2272 of 10 µmol/L in the first 3 min, a reduction of the positive hump of the *I*/*V* curve is noted, with a decrease in the amplitude of negative current (at −80 and −90 mV) and an increase *V_0_*. After 6 min, there are no fundamental changes compared to 3 min. Legend ‐ control: circles, perfusion of BAY41‐2272 3 min: triangles, perfusion of BAY41‐2272 6 min: squares. (d) Changes in *I*_L_ circles in control, 6 min after application of 10 µmol/L of BAY41‐2272 (triangles), after 3 (squares), 6 (inverted triangles), and 9 (rhombuses) min after additional application 200 µmol/L of SNAP

The change in the net membrane current time course at *V*_h_ =−45 mV, in the presence of different concentrations of BAY 41‐2272 in $K_{\text{in}}^{+}$/$K_{\text{out}}^{+}$ medium, did not reveal significant differences in the value of compound‐induced current. At all tested concentrations, BAY 41‐2272 shifted the current at −45 mV to more negative values. Figure 9a (curve ‐ a) shows changes in the net membrane current after cell perfusion with BAY 41‐2272 solution in a concentration of 5 µmol/L. Similar registrations were obtained by using BAY 41‐2272 at concentrations of 10, 50, and 100 µmol/L. Figure 9b shows *I*/*V*‐curves of the late current *I*_L_ obtained in the experiment shown in Figure 9a (curve ‐ a), in the control (circles) and 5 µmol/L BAY41‐2272‐induced currents through nonselective cation channel *I*_L,BAY_ after 5 min (triangles), 10 min (squares) and 15 min (rhombuses) perfusion. Since net membrane current at *V*_h_ = −45 mV in $K_{\text{in}}^{+}$/$K_{\text{out}}^{+}$ medium lies close to the positive hump of *I*/*V* curve (approximately at −50 mV) reflecting *I*_K1_ value, from the time course record, we can evaluate the dynamics of changes in this current over time. Therefore, according to data presented in Table 9, maximum peak current *ΔI*_max_, for example, at BAY 41‐2272 (10 µmol/L) was 0.178 ± 0.01 nA after 12.0 ± 0.8 min of perfusion, and these values did not show significant changes from *ΔI*_max_ at other compound concentrations, and in contrast to the two‐phase response to SNAP, did not undergo further changes. If on the background of cell perfusion with 5 µmol/L BAY 41‐2272 (when the net current approaches *ΔI*_max_), SNAP at a concentration of 200 µmol/L is introduced, it will not lead to further increase in the net current, but in contrary will decrease it, with the dynamic's characteristic for pure SNAP (Figure 9a, curve ‐ b). The same happened with the initial application of BAY 41‐2272 at the other concentrations.

**TABLE 9:** The effect of different concentrations of BAY41‐2272 in $K_{\text{in}}^{+}/K_{\text{out}}^{+}$ environments on the net membrane current and BAY41‐2272‐induced differential current (*ΔI*_max_) by means of online records (time‐course) at *V*_h_ = −45 mV. Mean ± SD, *n* = number of experiments, m = number of rates. In all cases, *p* > 0.05 and it was considered to indicate a statistically nonsignificant difference (*p* = NS)

$K_{\text{in}}^{+}$/$K_{\text{out}}^{+}$ (ionic composition)				
Parameters	BAY41‐2272, (µmol/L)			
	5	10	50	100
*n*	8	10	6	8
*m*	4	5	3	4
*t*_max_, (min)	12.2 ± 1.1	12.0 ± 0.8	11.8 ± 0.6	12.9 ± 0.6
*ΔI*_max_, (nA)	0.183 ± 0.01	0.178 ± 0.01	0.172 ± 0.008	0.170 ± 0.01

Figure 9c demonstrates measured *I*/*V* curves of the late *I*_L_ currents in control and currents through nonselective cation channels *I*_L,BAY_ after 3 (triangles) and 6 min (squares) BAY41‐2272 (10 µmol/L) perfusion. Table 10 shows values of *I*_L_, *I*_L,BAY_ currents, and BAY 41‐2272‐induced differential current (*ΔI*_BAY_), described from *I*/*V*‐ curves, (*I*_L_) at −45, −80, −90, and +40 mV after 3 and 6 min perfusion. Before the application of BAY 41‐2272 (Figure 9c circles), the *I*/*V* curve was *N*‐shaped and crossed the voltage axis at −70.3 ± 1 mV (*n* = 10). 10 µmol/L of BAY 41‐2272 during the first 3 min caused reduction of the positive hump of the *I*/*V* curve (approximately at −50 mV) (Figure 9c triangles compared to circles, also Table 10), and slightly shifted *V*_0_ toward the negative potential. After 6 min of perfusion, the positive hump value changed slightly (squares compared to triangles). At −80 mV, the value of ^3^ *I*_L,BAY_ was less than the control value but differed little from ^6^ *I*_L,BAY_. The differential currents ^3/C^ *ΔI*_BAY_ and ^6/C^Δ*I*_BAY_ also differed little. Since BAY 41‐2272 reduces the slope between ‐ 80 and −100 mV, for further analysis, we took the point of −90 mV. At −90 mV, the value of *^3^I*_L,BAY_ was significantly lower than the control values, but ^3/C^ *ΔI*_BAY_ and ^6/C^ *ΔI*_BAY_ differed little. BAY 41‐2272 had almost no effect on outward currents at +40 mV.

**TABLE 10:** The amplitude of BAY41‐2272‐induced current through nonselective cation channels *I*_L,BAY_, differential current *ΔI*_BAY_, *I*_L_, and differential current after additional SNAP application (*I*_L,BAY+SNAP_ and *ΔI*_BAY+SNAP_, respectively), described from *I*/*V* curves of the late current (*I*_L_) at −45, −80, −90 and +40 mV at 10 µmol/L of BAY41‐2272 after 3 and 6 min of perfusion and subsequent addition of SNAP against the background of continued BAY41‐2272 perfusion. Holding potential (*V*_h_) = −45 mV. *V*_0_ – the intercept of the resulting *I*/*V* curve with the voltage axis defined the zero current potential (*E*_0_) that corresponded to the resting membrane potential of a nonclamped cell (between −70 and −80 mV). $K_{\text{in}}^{+}/K_{\text{out}}^{+}$ solutions. Mean ± SD, *n* = number of experiments (cells), *m* = number of rats. *I*_L_ (nA) − measured value *I*/*V* curves of current. The differential current *ΔI*_BAY_ and *ΔI*_BAY+SNAP_ that occurs when the values of *I*_L_ are shifted in a more negative direction relative to the reference values is indicated by a minus (−), and the differential current when the values of *I*_L_ are shifted in a more positive direction is indicated by a plus (+). A *p* > 0.05 was considered to indicate a statistically nonsignificant difference (*p* = NS). All other instances with *p* < 0.01 are not indicated. ^#^ *p* = NS versus C10, *p = NS versus C13 and versus C19, ^†^ *p* = NS versus C11, ^††^ *p* = NS versus C11 and versus C17 and versus C20

Compounds	*V*, (mV)	*n*	*m*	Control		BAY41‐2272 10 μmol/L, 3 min perfusion			BAY41‐2272 10 μmol/L, 6 min perfusion		
				*V*_0_ (mV)	*I*_L_ (nA)	*V*_0_ (mV)	^3^ *I*_L,BAY_ (nA)	^3/C^ *ΔI*_BAY_ (nA)	*V*_0_ (mV)	^6^ *I*_L,BAY_ (nA)	^6/C^ *ΔI*_BAY_ (nA)
Columns	1	2	3	4	5	6	7	8	9	*10*	11
Clamp steps from *V*_h_ to	−45	10	7	−70 ± 1	+0.267 ± 0.04	−69 ± 1	+0.234 ± 0.04^#^	(−)0.019 ± 0.008^†^	−71 ± 1	+0.230 ± 0.05	(−)0.034 ± 0.01
	−80	10	7		−0.250 ± 0.02		−0.227 ± 0.03^#^	(+)0.042 ± 0.009^†^		−0.219 ± 0.02	(+)0.067 ± 0.01
	−90	10	7		−0.591 ± 0.05		−0.458 ± 0.06^#^	(+)0.121 ± 0.01^†^		−0.502 ± 0.06	(+)0.128 ± 0.02
	+40	10	7		+0.434 ± 0.04		+0.389 ± 0.04^#^	(+)0.062 ± 0.01^†^		+0.380 ± 0.04	(+)0.088 ± 0.02

Continuation of BAY41−2272 10 μmol/L; SNAP, 200 μmol/L ; 3 min perfusion			Continuation of BAY41−2272 10 μmol/L ; SNAP, 200 μmol/L ; 6 min perfusion			Continuation of BAY41−2272 10 μmol/L ; SNAP, 200 μmol/L ; 9 min perfusion		
*V*_0_ (mV)	^1^ *I*_L,BAY+SNAP_ (nA)	^1/C^ *ΔI*_BAY+SNAP_ (nA)	*V*_0_ (mV)	^3^ *I*_L,BAY+SNAP_ (nA)	^3/C^ *ΔI*_BAY+SNAP_ (nA)	*V*_0_ (mV)	^6^ *I*_L,BAY+SNAP_ (nA)	^6/C^ *ΔI*_BAY+SNAP_ (nA)
12	13	14	15	16	17	18	19	20
−65 ± 1	+0.212 ± 0.04	(−)0.035 ± 0.02^††^	−70 ± 1	+0.235 ± 0.05*	(−)0.051 ± 0.008	−72 ± 2	+0.224 ± 0.05	(−)0.049 ± 0.005
	−0.452 ± 0.05	(−)0.202 ± 0.002		−0.174 ± 0.03	(+)0.104 ± 0.03		−0.132 ± 0.04	(+)0.135 ± 0.004
	−0.870 ± 0.07	(−)0.279 ± 0.01		−0.439 ± 0.06	(+)0.195 ± 0.06		−0.392 ± 0.07	(+)0.261 ± 0.07
	+0.396 ± 0.05	(−)0.038 ± 0.006		+0.357 ± 0.05*	(−)0.094 ± 0.03		+0.355 ± 0.05	(−)0.127 ± 0.04

Decrease to 5 µmol/L or increase to 50 or 100 µmol/L concentration of BAY41‐2272 did not change the time course of late *I*_L_ currents on *I*/*V*‐curves. The values of BAY 41‐2272‐induced differential current *ΔI*_BAY_ described from *I*/*V*‐curves (*I*_L_) at −45, −80, and −90 mV, are presented in Table 11. In general, the use of 10 µmol/L BAY 41‐2272 reduces inward cation nonselective current *I*_L,ns_ already after 3 min and further action of the compound does not lead to significant effects.

**TABLE 11:** Value of BAY 41‐2272‐induced differential current *ΔI*_BAY_ described from *I*/*V*‐curves (*I*_L_) at −45, −80, and −90 mV at different concentrations of the compound after 5, 10, 15 min perfusion. $K_{\text{out}}^{+}$/$K_{\text{in}}^{+}$ solutions. Mean ± SD, *n* = number of experiments, m = number of rates. *I* (nA) − measured value of current. The differential current *ΔI*_BAY_ that occurs when the values of *I*_L_ are shifted to a more negative direction relative to the reference values is indicated by a minus (−), and the differential current when the values of *I*_L_ are shifted to a more positive direction is denoted by a plus (+). A *p* > 0.05 was considered to indicate a statistically nonsignificant difference (*p* = NS). All other instances with *p* < 0.01 are not indicated. ^#^ *p* = NS versus ^15/C^ *ΔI*_BAY_, *p = NS versus ^5/C^ *ΔI*_BAY_ and versus ^15/C^ *ΔI*_BAY_

BAY µmol/L	*V*, mV	*n*	*m*	Control	5 min perfusion	10 min perfusion	15 min perfusion
				*I* (nA)	^5/C^ *ΔI*_BAY_ (nA)	^10/C^ *ΔI*_BAY_ (nA)	^15/C^ *ΔI*_BAY_ (nA)
5	−45	11	5	+0.360 ± 0.01	(−)0.101 ± 0.02	(−)0.153 ± 0.03^#^	(−)0.171 ± 0.05
	−80	19	5	−0.120 ± 0.03	0.00 ± 0.001	0.00 ± 0.001*	0.00 ± 0.001
	−90	19	5	−0.588 ± 0.05	(+)0.176 ± 0.02	(+)0.196 ± 0.02	(+)0.279 ± 0.03
50	−45	10	4	+0.320 ± 0.02	(−)0.095 ± 0.01	(−)0.133 ± 0.03^#^	(−)0.155 ± 0.04
	−80	9	4	−0.112 ± 0.07	0.00 ± 0.001	0.01 ± 0.001*	0.02 ± 0.001
	−90	9	4	−0.565 ± 0.04	(+)0.220 ± 0.02	(+)0.235 ± 0.02*	0.235 ± 0.02

In the following experiments, with further cell perfusion with BAY41‐2272 (10 µmol/L), after 3 or 6 min, additional SNAP (200 µmol/L) was added to the perfusion solution (Figure 9d). Starting from the first min during the next 3 min, a significant increase in ^3^ *I*_L,BAY+SNAP_ was recorded both at −80 and −90 mV, and after 6 min a pronounced decrease in ^6^ *I*_L,BAY+SNAP_ was noticed to be lower than the initial current at these potentials (Table 10).

No further decrease in currents with time was noticed, and the values of ^9^ *I*_L,BAY+SNAP_ changed insignificantly compared to ^6^ *I*_L,BAY+SNAP_. The differential currents ^6/C^ *ΔI*_L,BAY+SNAP_ and ^9/C^ *ΔI*_L,BAY+SNAP_ also slightly changed both at −80 and −90 mV (Table 10).

Thus, BAY 41‐2272 (10 μμmol/l) in an intact unstretched cell caused current reduction at the levels of −45, −80, −90 mV and almost did not affect the outward current at +40 mV. A decrease in BAY 41‐2272 concentration to 5 µmol/L or an increase to 50 µmol/L in experiments on unstretched cells does not cause significant differences. SNAP introduced into solution 6 min after the beginning of perfusion with BAY41‐2272, caused *I*_K1_ reduction only in 3 min, followed by increased *I*_L,ns_. After 6 min of applying the cocktail, currents return to values slightly lower than the control ones.

### 3.9 BAY 41‐2272 abolishes *I*_SAC_ which remain unaffected at *EC_50_* concentration

**FIGURE 10:**
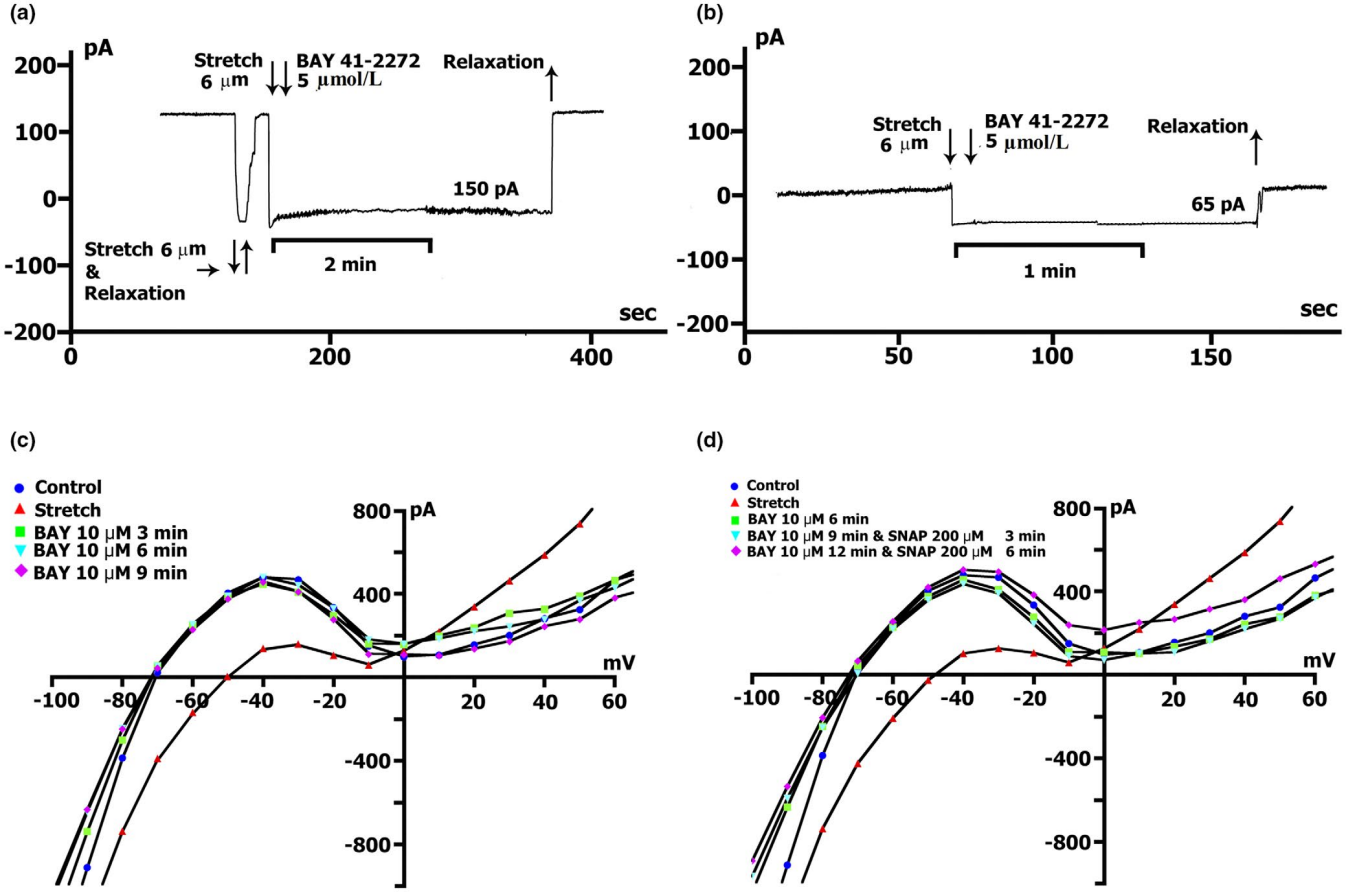
Changes in *I*_SAC_ in a stretched cell under the action of BAY41‐2272. (a) demonstrates the appearance of the *I*_SAC(−45)_ with a value of −0.150 nA (−0.195 ± 0.009 nA in control, *n* = 36) when the cell is stretched by 6 µm (3 min) in $K_{\text{in}}^{+}$/$K_{\text{out}}^{+}$ medium with 5 µmol/L of BAY41‐2272. (b) shows the *I*_SAC(−45)_ with a value of −0.065 nA (−0.082 ± 0.011 nA in control, *n* = 5) generated by a stretch of 6 µm (1.5 min) in ${\text{Cs}}_{\text{in}}^{+}$/${\text{Cs}}_{\text{out}}^{+}$ medium with 5 µmol/L of BAY41‐2272. (c) Voltage dependence of *I*_L_ in control (circles), after cell stretch by 6 µm (triangles), after 3 (squares), 6 (inverted triangles), and 9 (rhombuses) min of perfusion of BAY41‐2272 (10 µmol/L). (d) Voltage dependence of *I*_L_ in control (circles), after cell stretch by 6 µm (triangles), after 6 (squares) min of perfusion of BAY41‐2272 (10 µmol/L), and after 3 (inverted triangles) and 6 (rhombuses) min of perfusion after additional application of SNAP (200 µmol/L)

Figure 10a demonstrates appearance of *I*_SAC(−45)_ with a value of −0.150 nA (−0.195 ± 0.009 nA in control, *n* = 36), when cell was stretched by 6 µm, in $K_{\text{in}}^{+}$/$K_{\text{out}}^{+}$ medium. Figure 10b shows *I*_SAC(−45)_ generated by a 6 µm stretch in ${\text{Cs}}_{\text{in}}^{+}$/${\text{Cs}}_{\text{out}}^{+}$ medium, which causes a current equal to −0.065 nA (−0.082 ± 0.011 nA in control, *n* = 5). In both cases, the introduction of BAY 41‐2272 (5 µmol/L) on the background of *I*_SAC(−45)_, did not lead to any change in the recorded current. The absence of changes in the *I*_SAC(−45)_ could be explained by the low concentration of the active substance or the short time of detection of open *SACs* on the background of BAY 41‐2272.

The increased concentration of BAY 41‐2272 to 10 µmol/L, lead to elimination of the stretch‐induced currents. Figure 10c and Table 12 shows that currents induced by a 6 µm cell stretch (triangles compared to circles) are greatly reduced after 3 min perfusion with BAY 41‐2272 (10 µmol/L squares compared to triangles). After 6 min, *I*_K1_ at −45 mV was close to the initial values, and ^6^ *I*_L,ns,BAY_ at −80 mV did not differ from the control values, and at −90 mV it was significantly lower. The differential current values ^6/C^ *I*_SAC,BAY_ after 6 min at −80 and −90 mV shows a similar return to the values lower than the initial ones (Table 12). Additional introduction of SNAP (200 µmol/L) on the background of BAY41‐2272 (10 µmol/L) and 6 µm cell stretch slightly reduces ^3^ *I*_L,ns,BAY+SNAP_ in comparrison to ^6^ *I*_L,ns,BAY_, after 3 min at −80 and −90 mV. The BAY + SNAP cocktail, results in insignificant changes of the ^6^ *I*_L,ns,BAY+SNAP_ currents after 6 min of perfusion at −80 and −90 mV, in comparrison to ^3^ *I*_L,ns,BAY+SNAP_ (Table 12; Figure 10d inverted triangles and rhombuses compared to squares).

**TABLE 12:** The amplitude of the current through stretch‐activated nonselective cation channels *I*_L,ns_, the differential current through stretch‐activated channels *I*_SAC_, *I*_L,ns_, and *I*_SAC_ after BAY41‐2272 application (10 µmol/L) on the background of cell stretching (*I*_L,ns,BAY_ and *I*_SAC,BAY_, respectively) and after additional SNAP application (200 µmol/L) against the background of continued perfusion of BAY41‐2272 (*I*_L,ns,BAY +SNAP_ and *I*_SAC,BAY+SNAP_, respectively) described from *I*/*V* curves of the late current (*I*_L_) at −45, −80, −90 and +40 mV. Holding potential (*V*_h_) = −45 mV. *V*_0_ − the intercept of the resulting *I*/*V*‐curve with the voltage axis defined the zero current potential (*E*_0_) that corresponded to the resting membrane potential of a nonclamped cell (between −70 and −80 mV). $K_{\text{in}}^{+}$/$K_{\text{out}}^{+}$ solutions. Mean ± SD, *n* = number of experiments (cells), m = number of rats. The differential currents *I*_SAC_, *I*_SAC,BAY_, and *I*_SAC,BAY+SNAP_ that occurs when the *I*_L,ns_ values are shifted to a more negative direction relative to the reference values is indicated by a minus (−), and the differential current when the *I*_L,ns_ values are shifted to a more positive direction is denoted by a plus (+). A *p* > 0.05 was considered to indicate a statistically nonsignificant difference (*p* = NS). All other instances with *p* < 0.01 are not indicated. ^#^ *p* = NS versus C10, ^†^ *p* = NS versus C17, ^††^ *p* = NS versus C11 and versus C17, *p = NS versus C11

Compounds	*V*, (mV)	*n*	*m*	Control		Stretch 6 µm			Stretch 6 µm + BAY41‐2272 10 μmol/L, perfusion −6 min		
				*V*_0_ (mV)	*I*_L_ (nA)	*V*_0_ (mV)	*I*_L,ns_ (nA)	*I*_SAC_ (nA)	*V*_0_ (mV)	^6^ *I*_L,ns,BAY_ (nA)	^6/C^ *I*_SAC, BAY_ (nA)
Columns	1	2	3	4	5	6	7	8	9	10	11
Clamp steps from *V*_h_ to	−45	5	4	−78 ± 2	+0.377 ± 0.03	−52 ± 2	+0.178 ± 0.06	(−)0.199 ± 0.1	−75 ± 2	+0.272 ± 0.06	(−)0.105 ± 0.05
	−80	5	4		−0.377 ± 0.02^#^		−0.553 ± 0.08	(−)0.176 ± 0.09		−0.376 ± 0.08	(+)0.136 ± 0.04
	−90	5	4		−0.860 ± 0.07		−1.011 ± 0.08	(−)0.150 ± 0.07		−0.755 ± 0.09	(+)0.178 ± 0.05
	+40	5	4		+0.491 ± 0.08		+0.575 ± 0.02	(+)0.132 ± 0.09		+0.394 ± 0.07	(−)0.097 ± 0.02

Continuation of perfusion of stretched cell with BAY41‐2272 10 μmol/L + SNAP 200 μmol/L, 3 min			Continuation of perfusion of stretched cell with BAY41‐2272 10 μmol/L + SNAP 200 μmol/L, 6 min		
*V*_0_ (mV)	^3^ *I*_L,ns,BAY+SNAP_ (nA)	^3/C^ *I*_SAC,BAY+SNAP_ (nA)	*V*_0_ (mV)	^6^ *I*_L,ns,BAY+SNAP_ (nA)	^6/C^ *I*_SAC, BAY+SNAP_ (nA)
12	13	14	15	16	17
−80 ± 2	+0.295 ± 0.05^#^	(−)0.082 ± 0.03^††^	−83 ± 3	+0.328 ± 0.06	(−)0.061 ± 0.02
	−0.306 ± 0.04	(+)0.070 ± 0.04^†^		−0.271 ± 0.04	(+)0.106 ± 0.03
	−0.662 ± 0.07^#^	(+)0.198 ± 0.06*		−0.606 ± 0.06	(+)0.254 ± 0.06
	+0.370 ± 0.06^#^	(−)0.121 ± 0.03^†^		+0.408 ± 0.02	(−)0.137 ± 0.04

Thus, BAY41‐2272 eliminates stretch‐induced inward currents such that at −45 and −80 mV, becoming lower than the original, while additional injection of 200 µmol/L SNAP caused *I*_K1_ increase followed by *I*_L,ns_ decrease.

Based on the results above, BAY41‐2272 in the NO‐dependent cGMP‐PKG pathway induces phosphorylation of the *SACs* opened by cell stretch, resulting in the complete elimination of stretch‐induced *I*_SAC_. The subsequent additional introduction of SNAP did not change the situation.

### 3.10 ODQ involvement in modulation of the membrane currents ***I***_L,ns,_ and ***I***_K1_ recorded in $K_{\text{in}}^{+}$/$K_{\text{out}}^{+}$ medium

Taking that NO donors caused sGC‐β subunit activation and triggers NO‐dependent NO‐cGMP‐PKG pathways, whereby BAY 41‐2272 induces activation of NO‐independent sGC, then logically their mixture will modulate the *I*_L,ns,_ and *I*_SAC_ via (sGCβ_1_ and sGCα_1_)‐cGMP‐PKG pathway. We examined the effects of blocking of the sGC with a specific ODQ blocker or 1H‐[1,2,4]oxadiazolo[4,3‐a]quinoxaline‐1‐one. Of the two heterodimeric sGC isoforms, sGCα_1_β_1_ appears to be the predominant form, expressed in ventricular cardiomyocytes (Cawley et al., 2011), and ODQ at a concentration of 5–10 µmol/L caused inhibition of the activaties of all sGC isoforms (Sips et al., 2011).

**FIGURE 11:**
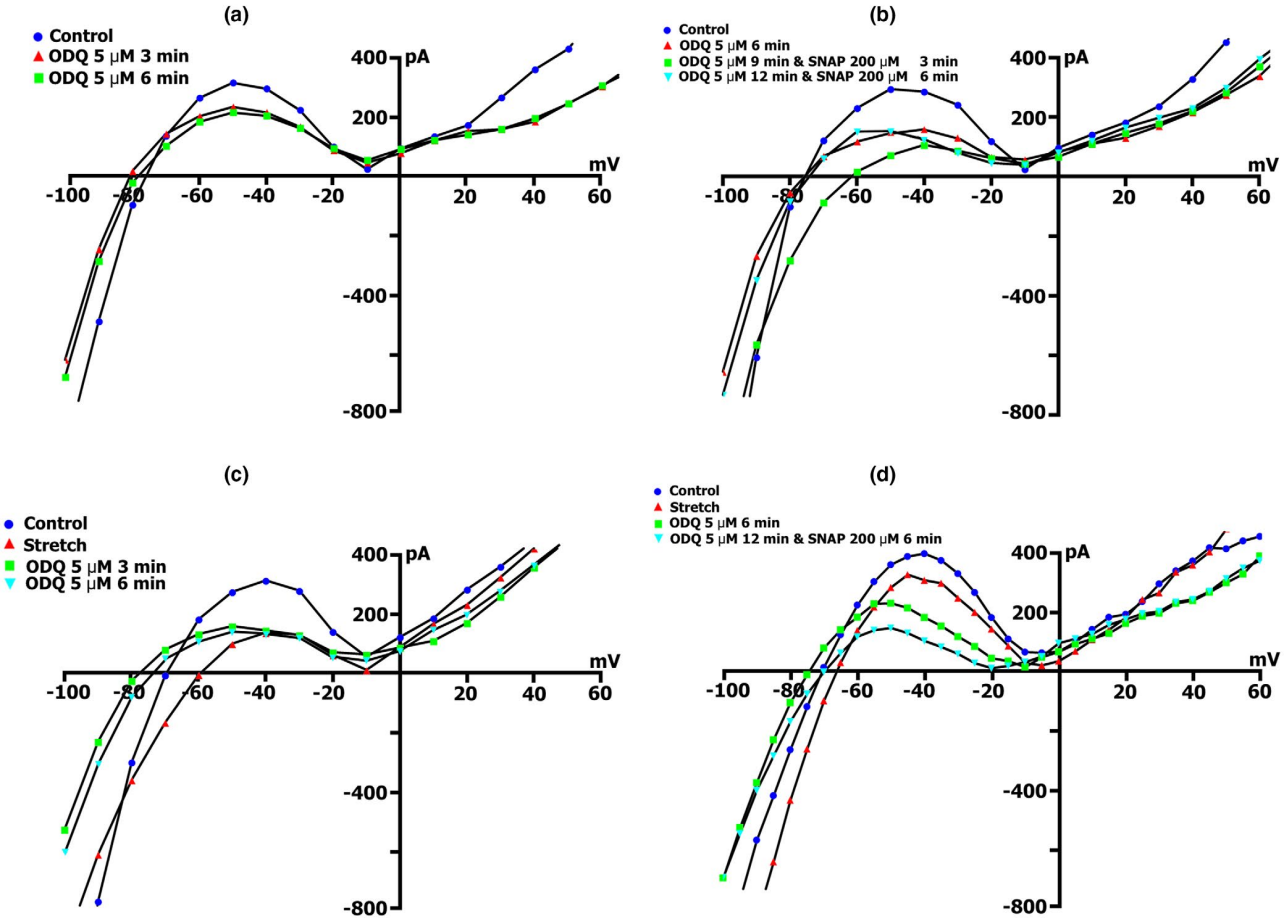
Effect of ODQ (5 μmol/L) and its combination with SNAP (200 μmol/L) on the *I*/*V* curve of late current (*I*_L_) and stretch‐activated cation nonselective current (*I*_L,ns_). (a) Changes in *I*_L_ in an intact cell against the background of constant ODQ perfusion in the control (circles), after 3 min (triangles), and after 6 min (squares). (b) changes in *I*_L_ under the action of ODQ followed by the addition of SNAP to the solution. Circles ‐ control, triangles −6 min of cell perfusion with ODQ, squares ‐ 3 min of perfusion after addition to the SNAP solution, inverted triangles ‐ 6 min with SNAP. (c), *I*_SAC_ changes in the stretched control cell (circles), after stretching by 6 µm (triangles), after 3 min (squares), and after 6 min (inverted triangles) of constant ODQ perfusion on the background of stretching. (d) Changes in *I*_SAC_ in the stretched control cell (circles), after stretching by 6 µm (triangles), after 6 min of continuous ODQ perfusion (squares), and after 6 min of perfusion after adding to the SNAP solution on the background of continuing stretch (inverted triangles)

In our experiments, we did not obtain any difference between the currents at all potentials during perfusion with 5 and 10 µmol/L ODQ (*p* = ns, *n* = 8 and *n* = 4, respectively, not shown). Figure 11a demonstrates changes in *I*/*V* curves of the late *I*_L_ currents in control (circles) and during ODQ (5 µmol/L), perfusion after 3 and 6 min (triangles and squares compared to circles, respectively), while Table 13 shows the values of these currents and the values of ODQ‐induced differential current (*ΔI*_ODQ_), described from the *I*/*V*‐curves, (*I*_L_) at −45, −80, −90 and +40 mV after 3 and 6 min perfusion. ODQ does not change the *N*‐shaped *I*/*V* curve but caused *I*_K1_ and *I*_L,ODQ_ reduction and *V*_0_ shift toward negative potentials. The differential currents ^3/C^ *ΔI*_ODQ,_ and ^6/C^ *Δ*_ODQ_, at the levels of −80 and −90 mV, did not differ much from each other. Furthermore, after a pronounced decrease at +40 mV during the first 3 min, ^6/C^ *ΔI*_ODQ_ remain stable in comparison to ^3/C^ *ΔI*_ODQ_ (Table 13).

**TABLE 13:** The amplitude of ODQ‐induced current through nonselective cation channels *I*_L, ODQ_, differential current *ΔI*_ODQ_, *I*_L,_ and differential current after additional SNAP application (*I*_L,ODQ+SNAP_ and *ΔI*_ODQ+SNAP_, respectively), described from *I*/*V* curves of late current (*I*_L_) at −45, −80, −90 and +40 mV at 5 µmol/L of ODQ after 3 and 6 min of perfusion and subsequent addition of SNAP against the background of continued ODQ perfusion. Holding potential (*V*_h_) = −45 mV. *V*_0_ – the intercept of the resulting *I*/*V* curve with the voltage axis defined the zero current potential (*E*_0_) that corresponded to the resting membrane potential of a nonclamped cell (between −70 and −80 mV). $K_{\text{in}}^{+}/K_{\text{out}}^{+}$ solutions. Mean ± SD, *n* = number of experiments (cells), *m* = number of rats. *I*_L_ (nA) ‐ measured value *I*/*V* curves of current. The differential current *ΔI*_ODQ_ and *ΔI*_ODQ+SNAP_ that occurs when the values of I_L_ are shifted in a more negative direction relative to the reference values is indicated by a minus (−), and the differential current when the values of *I*_L_ are shifted in a more positive direction is indicated by a plus (+). A *p* > 0.05 was considered to indicate a statistically nonsignificant difference (*p* = NS). All other instances with *p* < 0.01 are not indicated. ^#^ *p* = NS versus C10, *p = NS versus C10 and versus C16, **p = NS versus C10, ^†^ *p* = NS versus C8 and C14, ^††^ *p* = NS versus C8, ^⊗^ *p* = NS versus C11

Compounds	*V*, (mV)	*n*	*m*	Control		ODQ 5 μmol/L, 3 min perfusion			ODQ 5 μmol/L, 6 min perfusion		
				*V*_0_ (mV)	*I*_L_ (nA)	*V*_0_ (mV)	^3^ *I*_L,ODQ_ (nA)	^3/C^ *ΔI*_ODQ_ (nA)	*V*_0_ (mV)	^6^ *I*_L,ODQ_ (nA)	^6/C^ *ΔI*_ODQ_ (nA)
Columns	1	2	3	4	5	6	7	8	9	10	11
Clamp steps from *V*_h_ to	−45	8	7	−78 ± 2	+0.436 ± 0.05	−82 ± 2	+0.302 ± 0.05^#^	(−)0.134 ± 0.01	−80 ± 1	+0.271 ± 0.04	(−)0.154 ± 0.02^†^
	−80	8	7		−0.146 ± 0.03		−0.088 ± 0.02^#^	(+)0.123 ± 0.06		−0.061 ± 0.02	(+)0.097 ± 0.05^††^
	−90	8	7		−0.573 ± 0.06		−0.237 ± 0.05^#^	(+)0.281 ± 0.05		−0.252 ± 0.04	(+)0.259 ± 0.05^††^
	+40	8	7		+0.449 ± 0.05		+0.281 ± 0.03^#^	(−)0.146 ± 0.02		+0.264 ± 0.03	(−)0.145 ± 0.02^††^

Continuation of ODQ 5 μmol/l; SNAP, 200 μmol/L; 3 min perfusion			Continuation of ODQ 5 μmol/L; SNAP, 200 μmol/L; 6 min perfusion		
*V*_0_ (mV)	^3^ *I*_L,ODQ+SNAP_ (nA)	^3/C^ *ΔI*_ODQ+SNAP_ (nA)	*V*_0_ (mV)	^6^ *I*_L,ODQ+SNAP_ (nA)	^6/C^ *ΔI*_ODQ+SNAP_ (nA)
12	13	14	15	16	17
−61 ± 1	+0.273 ± 0.06*	(−)0.194 ± 0.06	−80 ± 2	+0.268 ± 0.05**	(−)0.163 ± 0.04^⊗^
	−0.293 ± 0.04	(−)0.280 ± 0.02		−0.092 ± 0.02**	(+)0.052 ± 0.02^⊗^
	−0.548 ± 0.05	(−)0.062 ± 0.04		−0.279 ± 0.05**	(+)0.221 ± 0.06^⊗^
	+0.288 ± 0.06*	(−)0.151 ± 0.08		+0.276 ± 0.05**	(−)0.140 ± 0.02^⊗^

In the following experiments, during a further 6 min cell perfusion with ODQ (Figure 11b, triangles compared to circles), SNAP (200 µmol/L) was additionally applied into perfusion solution (Figure 11b squares compared to triangles). Despite the continuous blocking of the sGC by ODQ, application of SNAP after 3 min caused a significant increase in the ^3^ *I*_L,ODQ+SNAP_ at −80 mV, while ^3/C^ *ΔI*_ODQ+SNAP_ was equal to (−) 0.280 ± 0.02 nA, that is, slightly larger in comparison to cells stretched by 6 μm, and caused *V*_0_ shift to −61 ± 1 mV. At the same time, this cocktail practically did not change the current at −45 and +40 mV. These changes were transient and as early as 6 min the *I*/*V* curve of the *I*_L_ was similar to that recorded after 6 min of pure ODQ (Figure 11b, inverted triangles compared to squares, Table 13), and shifted toward hyperpolarization, (*V*_0_ is shifted in the opposite direction from the initial value).

Thus, the use of both 5 and 10 µmol/L ODQ in an intact cell after 3 min induced reduction in the incoming cation nonselective current *I*_L,ODQ_ at −80 and −90 mV and caused *V*_0_ shift toward hyperpolarization. Both *I*_K1_ at −45 mV and outward current at +40 mV were decreased. SNAP, introduced into the solution 6 min after perfusion with ODQ, caused sharp *I*_L_ increase at −80 and partly at −90 mV during the first 3 min, hyperpolarizing *V*_0_, but slightly changing it at −45 and +40 mV. However, already after 6 min, the curve returns to the value of pure *I*_L,ODQ_.

### 3.11 ODQ abolishes ***I***_SAC_

Figure 11c (triangles compared to circles) demonstrates appearance of *I*_SAC_ during 6 µm cell stretch. Further application of ODQ (5 µmol/L) for 3 min (squares) eliminates not only *I*_SAC_, but also significantly reduces the initial current *I*_L,ns_. In this case, substantial shifts of the curve toward hyperpolarization were noticed. The values of *V*_0_, *I*_L,ns,ODQ_ and *I*_SAC_ at different time points are shown in Table 14.

**TABLE 14:** The amplitude of the current through stretch‐activated nonselective cation channels *I*_L,ns_, the differential current through stretch‐activated channels *I*_SAC_, *I*_L,ns_, and *I*_SAC_ after ODQ application (5 µmol/L) on the background of cell stretching (*I*_L,ns,ODQ_ and *I*_SAC, ODQ_, respectively) and after additional SNAP application (200 µmol/L) against the background of continued perfusion of ODQ (*I*_L,ns,ODQ+SNAP_ and *I*_SAC,ODQ+SNAP_, respectively) described from *I*/*V* curves of the late current (*I*_L_) at −45, −80, −90 and +40 mV. Holding potential (*V*_h_) = −45 mV. *V*_0_ − the intercept of the resulting *I*/*V*‐curve with the voltage axis defined the zero current potential (*E*_0_) that corresponded to the resting membrane potential of a nonclamped cell (between −70 and −80 mV). $K_{\text{in}}^{+}$/$K_{\text{out}}^{+}$ solutions. Mean ± SD, *n* = number of experiments (cells), *m* = number of rats. The differential currents *I*_SAC_, *I*_SAC,ODQ_, and *I*_SAC,ODQ+SNAP_ that occur when the *I*_L,ns_ values are shifted to a more negative direction relative to the reference values are indicated by a minus (−), and the differential current when the *I*_L,ns_ values are shifted to a more positive direction is denoted by a plus (+). A *p* > 0.05 was considered to indicate a statistically nonsignificant difference (*p* = NS). All other instances with *p* < 0.01 are not indicated. ^#^ *p* = NS versus C17

Compounds	*V*, (mV)	*n*	*m*	Control		Stretch 6 µm			Stretch 6 µm + ODQ 5 μmol/L, perfusion – 6 min		
				*V*_0_ (mV)	*I*_L_ (nA)	*V*_0_ (mV)	*I*_L,ns_ (nA)	*I*_SAC_ (nA)	*V*_0_ (mV)	^6^ *I*_L,ns,ODQ_ (nA)	^6/C^ *I*_SAC,ODQ_ (nA)
Columns	1	2	3	4	5	6	7	8	9	10	11
Clamp steps from *V*_h_ to	−45	5	4	−75 ± 2	+0.317 ± 0.06	−65 ± 2	+0.196 ± 0.05	(−)0.121 ± 0.04	−80 ± 1	+0.167 ± 0.02	(−)0.150 ± 0.03
	−80	5	4		−0.341 ± 0.05		−0.480 ± 0.04	(−)0.139 ± 0.04		−0.124 ± 0.04	(+)0.217 ± 0.04
	−90	5	4		−0.717 ± 0.1		−0.869 ± 0.10	(−)0.152 ± 0.05		−0.405 ± 0.10	(+)0.323 ± 0.05
	+40	5	4		+0.495 ± 0.08		+0.477 ± 0.08	(+)0.018 ± 0.04		+0.320 ± 0.03	(−)0.185 ± 0.03

Continuation of perfusion of stretched cell with ODQ 5 μmol/L + SNAP, 200 μmol/L, 3 min			Continuation of perfusion of stretched cell with ODQ 5 μmol/L + SNAP, 200 μmol/L, 6 min		
*V*_0_ (mV)	^3^ *I*_L,ns,ODQ+SNAP_ (nA)	^3/C^ *I*_SAC,ODQ+SNAP_ (nA)	*V*_0_ (mV)	^6^ *I*_L,ns,ODQ+SNAP_ (nA)	^6/C^ *I*_SAC,ODQ+SNAP_ (nA)
12	13	14	15	16	17
−75 ± 2	0.129 ± 0.02	(−)0.188 ± 0.04^#^	−74 ± 3	+0.047 ± 0.01	(−)0.210 ± 0.03
	−0.247 ± 0.05	(+)0.094 ± 0.02		−0.347 ± 0.08	(+)0.006 ± 0.03
	−0.284 ± 0.04	(+)0.252 ± 0.03		−0.262 ± 0.03	(+)0.115 ± 0.02
	+0.246 ± 0.03	(−)0.179 ± 0.02^#^		+0.339 ± 0.02	(−)0.168 ± 0.03

Figure 11d and Table 14 demonstrates that additional application of SNAP (200 µmol/L) on the background of 6 µm cell stretch after 6 min perfusion with ODQ (5 µmol/L) (triangles compared to circles in control), causes *I*_L,ns,ODQ+SNAP_ reduction at −40 mV, but *I*_L,ns,ODQ+SNAP_ increasing at −80 and −90 mV, and did not reach the control values (inverted triangles).

Thus, ODQ eliminates the inward stretch‐induced currents *I*_L,ns_, while the currents at the levels of −45 and −80 mV become significantly lower than initial ones, whereby an additional injection of 200 µmol/L SNAP increases them, but not the control values.

### 3.12 Involvement of KT‐5823 in modulation of the membrane currents ***I***_L,ns,_ and ***I***_K1_ recorded in $K_{\text{in}}^{+}$/$K_{\text{out}}^{+}$ medium

In this series of experiments, intact cardiomyocytes were perfused with KT5823 (1 µmol/L), which is known to be an inhibitor of cGMP‐dependent protein kinase in the NO‐sGC‐cGMP‐PKG pathway.

**FIGURE 12:**
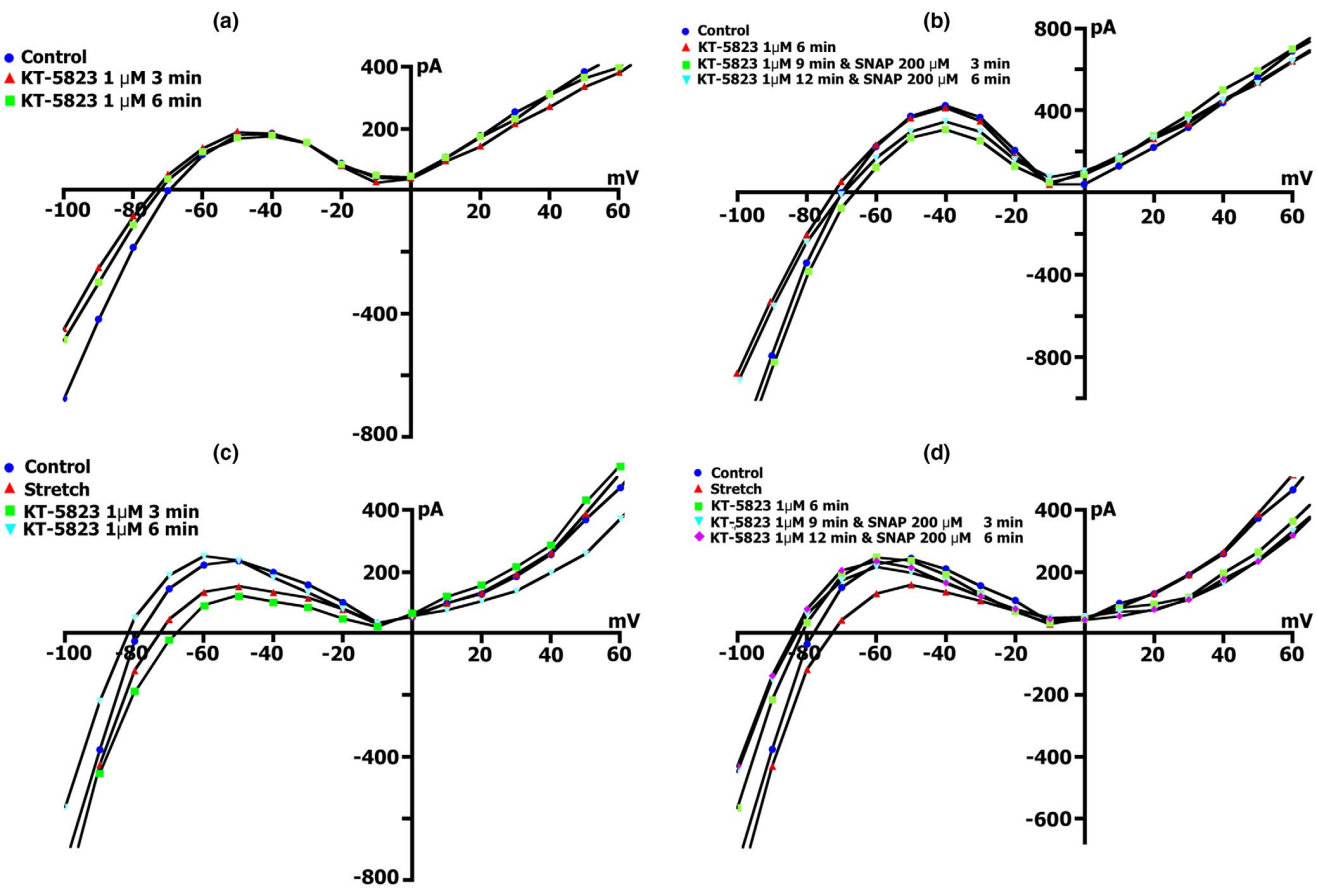
Effect of KT5823 (1 μmol/L) and its combination with SNAP (200 μmol/L) on late current (*I*_L_) *I*/*V* curve and stretch‐activated cation nonselective current (*I*_L,ns_). (a) Changes in *I*_L_ in an intact cell against the background of constant perfusion of KT5823 in the control (circles), after 3 min (triangles), and after 6 min (squares). (b) changes in *I*_L_ under the action of KT5823 with the subsequent addition of SNAP to the solution. Circles ‐ control, triangles ‐ 6 min perfusion of cells with KT5823, squares ‐ 3 min perfusion after addition to SNAP solution, inverted triangles ‐ 6 min with SNAP. (c) *I*_SAC_ changes in the stretched control cell (circles), after stretching by 6 µm (triangles), after 3 min (squares), and after 6 min (inverted triangles) of constant perfusion of KT5823 on the background of stretching. (d) *I*_SAC_ changes in the stretched control cell (circles), after stretching by 6 µm (triangles), after 6 min of constant perfusion of KT5823 (squares) and after 3 min (inverted triangles) and 6 min (rhombuses) of perfusion after addition to the solution SNAP against the backdrop of ongoing stretching

Figure 12a shows *I*/*V* curve of the *I*_L_ in control and $K_{\text{in}}^{+}$/$K_{\text{out}}^{+}$ medium (circles) and its changes under the action of KT5823 after 3 min (triangles), and after 6 min (squares), while the mean values of *I*_L,KT_, *I*_K1,_ and *ΔI*_KT_ are summarized in Table 15. From the presented data, it follows that perfusion of cells with KT5823, practically did not change the *I*_K1_ after 3 min, (which is the current at −45 mV), but caused ^3^ *I*_L,KT_ reduction at −80 and −90 mV. After 6 min, the measured currents values did not change. There were no further changes in the *I*_L_ current curve parameters (not shown). The output current at the level of +40 mV did not change. Thus, under the action of KT5823, only the incoming nonselective cation current decreased.

**TABLE 15:** The amplitude of the KT‐5823‐induced current through nonselective cation channels *I*_L,KT_, differential current *ΔI*_KT_, *I*_L_ and differential current after additional SNAP application (*I*_L,KT+SNAP_ and *ΔI*_KT+SNAP_, respectively), described from *I*/*V* curves of the late current (*I*_L_) at −45, −80, −90, and +40 mV at 1 µmol/L of KT‐5823 after 3 and 6 min of perfusion and subsequent addition of SNAP against the background of continued KT‐5823 perfusion. Holding potential (*V*_h_) = −45 mV. *V*_0_ – the intercept of the resulting *I*/*V* curve with the voltage axis defined the zero current potential (*E*_0_) that corresponded to the resting membrane potential of a nonclamped cell (between −70 and −80 mV). $K_{\text{in}}^{+}$/$K_{\text{out}}^{+}$ solutions. Mean ± SD, *n* = number of experiments (cells), *m* = number of rats. *I*_L_ (nA) − measured value *I*/*V* curves of current. The differential current *ΔI*_KT_ and *ΔI*_KT+SNAP_ that occurs when the values of *I*_L_ are shifted in a more negative direction relative to the reference values are indicated by a minus (−), and the differential current when the values of *I*_L_ are shifted in a more positive direction is indicated by a plus (+). A *p* > 0.05 was considered to indicate a statistically nonsignificant difference (*p* = NS). All other instances with *p* < 0.01 are not indicated. ^#^ *p* = NS versus C10, *p = NS versus C5 and versus C10, ^†^ *p* = NS versus C8, ^††^ *p* = NS versus C14, ^##^ *p* = NS versus C17, ^**^ *p* = NS versus C17

Compounds	*V*, (mV)	*n*	*m*	Control		KT−5823 1 μmol/L, 3 min perfusion			KT−5823 1 μmol/L, 6 min perfusion		
				*V*_0_ (mV)	*I*_L_ (nA)	*V*_0_ (mV)	^3^ *I*_L,KT_ (nA)	^3/C^ *ΔI*_KT_ (nA)	*V*_0_ (mV)	^6^ *I*_L,KT_ (nA)	^6/C^ *ΔI*_KT_ (nA)
Columns	1	2	3	4	5	6	7	8	9	10	11
Clamp steps from V_hp_ to	−45	5	4	−75 ± 1	+0.267 ± 0.03	−75 ± 1	+0.254 ± 0.07*	(−)0.016 ± 0.007	−82 ± 1	+0.245 ± 0.05	(−)0.021 ± 0.06^†^
	−80	5	4		−0.265 ± 0.04		−0.178 ± 0.04^#^	(+)0.121 ± 0.01		−0.193 ± 0.03	(+)0.019 ± 0.01^††^
	−90	5	4		−0.666 ± 0.06		−0.449 ± 0.10^#^	(+)0.228 ± 0.05		−0.460 ± 0.05	(+)0.106 ± 0.02
	+40	5	4		+0.349 ± 0.02		+0.370 ± 0.05*	(+)0.042 ± 0.01		+0.369 ± 0.03	(+)0.014 ± 0.01

Continuation of KT‐5823 1 μmol/L; SNAP, 200 μmol/L; 3 min perfusion			Continuation of KT‐5823 1 μmol/L; SNAP, 200 μmol/L; 6 min perfusion		
*V*_0_ (mV)	^3^ *I*_L,KT+SNAP_ (nA)	^3/C^ *ΔI*_KT+SNAP_ (nA)	*V*_0_ (mV)	^6^ *I*_L,KT+SNAP_ (nA)	^6/C^ *ΔI*_KT+SNAP_ (nA)
12	13	14	15	16	17
−65 ± 1	0.212 ± 0.04	(−)0.069 ± 0.02^**^	−73 ± 1	0.254 ± 0.04	(−)0.054 ± 0.008
	−0.268 ± 0.04	(+)0.029 ± 0.002		−0.199 ± 0.03	(+)0.071 ± 0.02
	−0.667 ± 0.06	(+)0.045 ± 0.01		−0.479 ± 0.06	(+)0.209 ± 0.04
	0.399 ± 0.05^##^	(−)0.066 ± 0.006		0.414 ± 0.02	(−)0.038 ± 0.03

In further experiments, SNAP (200 µmol/L) was added after 6 min of KT5823 in $K_{\text{in}}^{+}$/$K_{\text{out}}^{+}$ medium. Figure 12b shows the *I*/*V* curve of the *I*_L_ current in control (circles), after 6 min of KT5823 perfusion (triangles), and after 3 (squares) and 6 (inverted triangles) min after additional administration of SNAP. Interestingly, SNAP after 3 min caused a decrease in *I*_K1_, but an increase in the inward cation nonselective current at both −80 and −90 mV. Furthermore, this increase in ^3^ *I*_L,KT+SNAP_ at these potentials reaches the control values, which means that SNAP, by one or another mechanism, eliminates the KT5823 induced *I*_L,ns_ inhibition. However, after 6 min perfusion with SNAP, the *^6^I*_L,KT+SNAP_ *I*/*V* curve returns to the ^6^ *I*_L,KT_ curve (Table 15). The output currents at +40 mV did not change at all.

Thus, under the action of KT5823, only the inward nonselective cation current decreased, and further addition of SNAP caused temporary activation (or removal of the inhibitory effect of KT5823) of *I*_L,ns_ followed by its elimination.

### 3.13 KT‐5823 caused stretch‐induced current increase followed by its inhibition

As shown in Figure 12c, cell stretch causes a stretch‐induced *I*_K1_ decrease and an increase in the *I*_L,ns_ (triangles vs. circles in control), the values of which and the values of *I*_SAC_ are given in Table 16. As happens with stretch, *V*_0_ shifts toward depolarization. On the background of the cell elongation, introduction of 1 µmol/L KT5823 after 3 min caused a temporary but even greater increase in *I*_SAC_ at the level of −80 and −90 mV (squares). The *I*_K1_ also shifted to less positive side. To a greater extent, *V*_0_ shifted to the region of depolarization. However, after 6 min perfusion (inverted triangles), *I*_SAC_ was markedly inhibited, while the current ^3^ *I*_L,ns,KT_, despite continued stretch, significantly lowered in comparison to the control conditions. Fully returned to the control values and *I*_K1_. At the same time, *V*_0_ shifted toward hyperpolarization and its value became greater than in the control conditions.

**TABLE 16:** The amplitude of the current through stretch‐activated nonselective cation channels *I*_Lns_, the differential current through stretch‐activated channels *I*_SAC_, *I*_L,ns_, and *I*_SAC_ after KT‐5823 application (1 µmol/L) on the background of cell stretching (*I*_L,ns,KT_ and *I*_SAC,KT_, respectively) and after additional SNAP application (200 µmol/L) against the background of continued perfusion of KT‐5823 (*I*_L,ns,KT+SNAP_ and *I*_SAC,KT+SNAP_, respectively) described from *I*/*V* curves of the late current (*I*_L_) at −45, −80, −90, and +40 mV. Holding potential (*V*_h_) = −45 mV. *V*_0_ − the intercept of the resulting *I*/*V*‐curve with the voltage axis defined the zero current potential (*E*_0_) that corresponded to the resting membrane potential of a nonclamped cell (between −70 and −80 mV). $K_{\text{in}}^{+}$/$K_{\text{out}}^{+}$ solutions. Mean ± SD, *n* = number of experiments (cells), *m* = number of rats. The differential currents *I*_SAC_, *I*_SAC,KT_ and *I*_SAC,KT+SNAP_ that occurs when the *I*_L,ns_ values are shifted to a more negative direction relative to the reference values are indicated by a minus (−), and the differential current when the *I*_L,ns_ values are shifted to a more positive direction is denoted by a plus (+). A *p* > 0.05 was considered to indicate a statistically nonsignificant difference (*p* = NS). All other instances with *p* < 0.01 are not indicated. ^#^ *p* = NS versus C16, *p = NS versus C10 and versus C16, ^†^ *p* = NS versus C11 and C17, ^††^ *p* = NS versus C17.

Compounds	*V*, (mV)	*N*	*m*	Control		Stretch 6 µm			Stretch 6 µm + KT−5823 1 μmol/L, perfusion ‐ 6 min		
				*V*_0_ (mV)	*I*_L_ (nA)	*V*_0_ (mV)	*I*_L,ns_ (nA)	*I*_SAC_ (nA)	*V*_0_ (mV)	^6^ *I*_L,ns,KT_ (nA)	^6/C^ *I*_SAC,KT_ (nA)
Columns	1	2	3	4	5	6	7	8	9	10	11
Clamp steps from *V*_h_ to	−45	5+	4	−80 ± 2	+0.238 ± 0.02	−67 ± 2	+0.120 ± 0.02	(−)0.114 ± 0.02	−85 ± 2	+0.230 ± 0.02	(+)0.021 ± 0.02
	−80	5+	4		−0.251 ± 0.03		−0.353 ± 0.03	(−)0.127 ± 0.03		−0.181 ± 0.03	(+)0.055 ± 0.04
	−90	5+	4		−0.548 ± 0.08		−0.647 ± 0.05	(−)0.210 ± 0.04		−0.351 ± 0.05	(+)0.210 ± 0.05
	+40	5+	4		+0.260 ± 0.01		+0.285 ± 0.01	(+)0.025 ± 0.02		+0.220 ± 0.02	(−)0.031 ± 0.02

Continuation of perfusion of stretched cell with KT−5823 1 μmol/L + SNAP 200 μmol/L, 3 min			Continuation of perfusion of stretched cell with KT−5823 1 μmol/L + SNAP 200 μmol/L, 6 min		
*V*_0_ (mV)	^3^ *I*_L,ns,KT+SNAP_ (nA)	^3/C^ *I*_SAC,KT+SNAP_ (nA)	*V*_0_ (mV)	^6^ *I*_L,ns,KT+SNAP_ (nA)	^6/C^ *I*_SAC,KT+SNAP_ (nA)
12	13	14	15	16	17
	+0.210 ± 0.02*	(−)0.022 ± 0.03^†^	−83 ± 3	+0.205 ± 0.02	(−)0.026 ± 0.02
	−0.165 ± 0.01*	(+)0.076 ± 0.02^†^		−0.170 ± 0.01	(+)0.079 ± 0.02
	−0.281 ± 0.02^#^	(+)0.276 ± 0.03^††^		−0.276 ± 0.02	(+)0.272 ± 0.03
	+0.200 ± 0.03*	(−)0.054 ± 0.02^††^		+0.208 ± 0.03	(−)0.052 ± 0.03

In further experiments (Figure 12d and Table 16), on the background of cell stretch (triangles vs. circles in control), after 6 min of KT5823 action (squares), a pronounced inhibition of *I*_SAC_, *I*_K1,_ and *V*_0_ shift toward hyperpolarization were noticed. Furthermore, SNAP (200 µmol/L) introduced into the $K_{\text{in}}^{+}$/$K_{\text{out}}^{+}$ medium after 3 (inverted triangles) and 6 (rhombuses) min inhibited all currents to an even greater extent than it did at the 6th min of KT5823 (rhombuses compared to squares).

Thus, application of KT5823 caused temporaril stretch‐induced current increase followed by subsequent inhibition while additionally introduced SNAP caused current inhibition to an even greater extent.

### 3.14 Involvement of 8Br‐cGMP in modulation of the membrane currents ***I***_L,ns,_ and ***I***_K1_ recorded in $K_{\text{in}}^{+}$/$K_{\text{out}}^{+}$ medium

In this series, cardiomyocytes were perfused with an analog of cGMP, 8‐Bromo‐cGMP (8Br‐cGMP), at a concentration of 200 µmol/L, commonly used for investigation of isolated cardiomyocyte's currents (Koitabashi et al., 2010).

**FIGURE 13:**
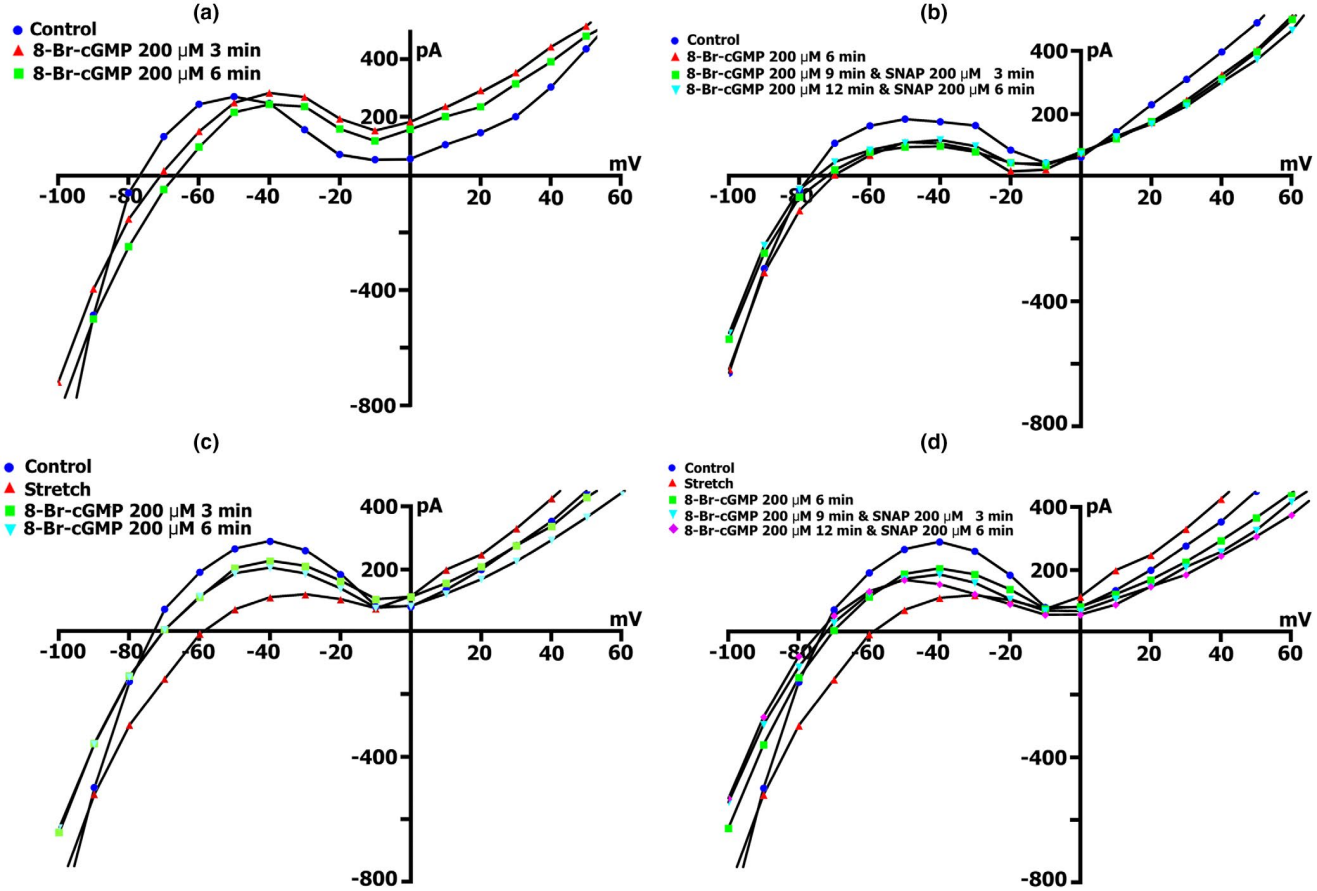
Effect of 8Br‐cGMP (200 μmol/L) and its combination with SNAP (200 μmol/L) on late current (*I*_L_) *I*/*V* curve and stretch‐activated cation nonselective current (*I*_L,ns_). (a) Changes in *I*_L_ in an intact cell against the background of its constant perfusion with 8Br‐cGMP in the control (circles), after 3 min (triangles), and after 6 min (squares). (b) changes in *I*_L_ under the action of 8Br‐cGMP with the subsequent addition of SNAP to the solution. Circles ‐ control, triangles ‐ 6 min cell perfusion with 8Br‐cGMP, squares ‐ 3 min perfusion after addition to SNAP solution, inverted triangles ‐ 6 min with SNAP. (c) *I*_SAC_ changes in the stretched control cell (circles), after stretching by 6 µm (triangles), after 3 min (squares), and after 6 min (inverted triangles) of continuous 8Br‐cGMP perfusion on the background of stretching. (d) *I*_SAC_ changes in the stretched control cell (circles), after stretching by 6 µm (triangles), after 6 min of constant perfusion with 8Br‐cGMP (squares), and after 3 min (inverted triangles) and 6 min (rhombuses) of perfusion after addition into the SNAP solution against the background of ongoing stretching

Figure 13a shows the *I*/*V* curve of the *I*_L_ current in control and $K_{\text{in}}^{+}$/$K_{\text{out}}^{+}$ medium (circles) and its changes under the action of 8Br‐cGMP after 3 min (triangles) and after 6 min (squares), while the average values of *I*_L,8Br‐cGMP_, *I*_K1,_ and *ΔI*_8Br‐cGMP_ are summarized in Table 17. From the presented data, it follows that perfusion of cell with 8Br‐cGMP solution after 3 min causes *I*_K1_ reduction to the level of −45 mV, and increased ^3^ *I*_L,8Br‐cGMP_ to the levels of −80 and −90 mV. After 6 min, the values of ^3^ *I*_L,8Br‐cGMP_ increased even more. At the same time, during the registration period, a shift of *V*_0_ toward depolarization was noticed. There were no further time‐dependent changes in the *I*_L_. The output current at the level of +40 mV was insignificantly changed.

**TABLE 17:** Amplitude of the 8Br‐cGMP‐induced current through nonselective cation channels *I*_L,8Br‐cGMP_, differential current *ΔI*_8Br‐cGMP_, *I*_L_ and differential current after additional SNAP application (*I*_L,8Br‐cGMP +SNAP_ and *ΔI*_8Br‐cGMP +SNAP_, respectively), described from *I*/*V* curves of the late current (*I*_L_) at −45, −80, −90, and +40 mV at 200 µmol/L of 8Br‐cGMP after 3 and 6 min of perfusion and subsequent addition of SNAP against the background of continued 8Br‐cGMP perfusion. Holding potential (*V*_h_) = −45 mV. *V*_0_ ‐ the intercept of the resulting *I*/*V* curve with the voltage axis defined the zero current potential (*E*_0_) that corresponded to the resting membrane potential of a nonclamped cell. $K_{\text{in}}^{+}$/$K_{\text{out}}^{+}$ solutions. Mean ± SD, *n* = number of experiments (cells), *m* = number of rats. *I*_L_ (nA) ‐ measured value *I*/*V* curves of current. The differential current *ΔI*_8Br‐cGMP_ and *ΔI*_8Br‐cGMP+SNAP_ that occurs when the values of *I*_L_ are shifted in a more negative direction relative to the reference values are indicated by a minus (−), and the differential current when the *I*_L_ values are shifted in a more positive direction is indicated by a plus (+). A *p* > 0.05 was considered to indicate a statistically nonsignificant difference (*p* = NS). All other instances with *p* < 0.01 are not indicated. ^#^ *p* = NS versus C7 and C13, *p = NS versus C7, ^##^ *p* = NS versus C13, ^†^ *p* = NS versus C8, ^††^ *p* = NS versus C8 and versus C14, **p = NS versus C14

Compounds	*V*, (mV)	*n*	*m*	Control		8Br‐cGMP 200 μmol/L, 3 min perfusion			8Br‐cGMP 200 μmol/L, 6 min perfusion		
				*V*_0_ (mV)	*I*_L_ (nA)	*V*_0_ (mV)	^3^ *I*_L,8Br‐cGMP_ (nA)	^3/C^ *ΔI*_8Br‐cGMP_ (nA)	*V*_0_ (mV)	^6^ *I*_L,8Br‐cGMP_ (nA)	^6/C^ *ΔI*_8Br‐cGMP_ (nA)
Columns	1	2	3	4	5	6	7	8	9	10	11
Clamp steps from *V*_h_ to	−45	6	4	−78 ± 1	+0.290 ± 0.05	70 ± 1	+0.232 ± 0.06	(−)0.061 ± 0.02	69 ± 1	+0.212 ± 0.06^#^	(−)0.088 ± 0.02 ^††^
	−80	6	4		−0.172 ± 0.06		−0.260 ± 0.07	(−)0.093 ± 0.03		−0.300 ± 0.06	(−)0.135 ± 0.05
	−90	6	4		−0.545 ± 0.07		−0.560 ± 0.08	(−)0.061 ± 0.02		−0.565 ± 0.08*	(−)0.066 ± 0.04 ^††^
	+40	6	4		+0.463 ± 0.06		+0.477 ± 0.06	(+)0.055 ± 0.03		+0.478 ± 0.06^#^	(+)0.056 ± 0.04 ^††^

Continuation of 8Br‐cGMP 200 μmol/L; SNAP, 200 μmol/L; 3 min perfusion			Continuation of 8Br‐cGMP 200 μmol/L; SNAP, 200 μmol/L; 6 min perfusion		
*V*_0_ (mV)	^3^ *I*_L,8Br‐cGMP +SNAP_ (nA)	^3/C^ *ΔI*_8Br‐cGMP +SNAP_ (nA)	*V*_0_ (mV)	^6^ *I*_L,8Br‐cGMP +SNAP_ (nA)	^6/C^ *ΔI*_8Br‐cGMP+SNAP_ (nA)
12	13	14	15	16	17
78 ± 1	+0.203 ± 0.04	(−)0.086 ± 0.03	78 ± 1	+0.205 ± 0.05^##^	(−)0.081 ± 0.01**
	−0.181 ± 0.06	(−)0.022 ± 0.02		−0.170 ± 0.05^##^	(+)0.016 ± 0.03**
	−0.530 ± 0.08	(+)0.049 ± 0.05		−0.525 ± 0.08^##^	(+)0.043 ± 0.02**
	+0.460 ± 0.04	(−)0.027 ± 0.04		+0.455 ± 0.04^##^	(−)0.038 ± 0.02**

In further experiments, 200 µmol/L SNAP was added after 6 min of 8Br‐cGMP. Figure 13b shows the *I*/*V* curve of the *I*_L_ current in control (circles), after 6 min of perfusion with 8Br‐cGMP (triangles) and after 3 (squares) and 6 (inverted triangles) min after additional administration of SNAP. On the background of 8Br‐cGMP, the introduction of SNAP after 3 and 6 min did not lead to significant changes in *I*_K1_, but caused inhibition of the inward cation nonselective current induced by 8Br‐cGMP. Furthermore, this SNAP‐induced inhibition of the 8Br‐cGMP‐induced current (^6^ *I*_L,8Br‐cGMP+SNAP_) decreased its values compared to the control current at the level of −80 and −90 mV. The introduction of SNAP reversed 8Br‐cGMP‐induced depolarization (Table 17). The output currents at the level of +40 mV did not change.

Thus, under the action of 8Br‐cGMP, an increase in the inward nonselective cation current and depolarization of *V*_0_ occurred for 6 min, after which no changes were observed, and further introduction of in‐medium SNAP eliminated this 8Br‐cGMP‐induced current.

### 3.15 8Br‐cGMP causes stretch‐induced current inhibition

As shown in Figure 13c cell stretch by 6 μm causes a stretch‐induced shift in the *I*_K1_ to less positive values and an *I*_L,ns_ increase (triangles vs. circles in control), which values, together with those of *I*_SAC_, are given in Table 18. The characteristic shift of *V*_0_ toward depolarization is shown. The introduction of 200 µmol/L 8Br‐cGMP on the background of cell elongation, caused restoration of the current to values close to the initial *I*_K1_ accompanied with significant inhibition of the ^3/C^ *I*_SAC,8Br‐cGMP_ at the levels of −80 and −90 mV, already after 3rd min (squares vs. triangles). Furthermore, *V*_0_ also returned to its original value. These changes persisted after 6 min of registration (inverted triangles).

**TABLE 18:** Amplitude of current through stretch‐activated nonselective cation channels *I*_L,ns_, differential current through stretch‐activated channels *I*_SAC_, *I*_L,ns_, and *I*_SAC_ after 8Br‐cGMP application (10 µmol/L) on the background of cell stretching (*I*_L,ns,8Br‐cGMP_ and *I*_SAC,8Br‐cGMP_, respectively) and after additional application of SNAP (200 µmol/L) against the background of continued perfusion of 8Br‐cGMP (*I*_L,ns,8Br‐cGMP+SNAP_ and *I*_SAC, 8Br‐cGMP +SNAP_, respectively) described from *I*/*V* curves of the late current (*I*_L_) at −45, −80, −90, and +40 mV. Holding potential (*V*_h_) = −45 mV. *V*_0_ − the intercept of the resulting *I*/*V*‐curve with the voltage axis defined the zero current potential (*E*_0_) that corresponded to the resting membrane potential of a nonclamped cell (between −70 and −80 mV). $K_{\text{in}}^{+}$/$K_{\text{out}}^{+}$ solutions. Mean ± SD, *n* = number of experiments (cells), *m* = number of rats. The differential currents *I*_SAC_, *I*_SAC,8Br‐cGMP_, and *I*_SAC, 8Br‐cGMP +SNAP_ that occurs when the *I*_L,ns_ values are shifted to a more negative direction relative to the reference values are indicated by a minus (−), and the differential current when the *I*_L,ns_ values are shifted to a more positive direction is denoted by a plus (+). A *p* > 0.05 was considered to indicate a statistically nonsignificant difference (*p* = NS). All other instances with *p* < 0.01 are not indicated. ^#^ *p* = NS versus C10 and versus C16, ^##^ *p* = NS versus C16 ^†^ *p* = NS versus C11 and versus C17, ^††^ *p* = NS versus C17

Compounds	*V*, (mV)	*n*	*m*	Control		Stretch 6 µm			Stretch 6 µm + 8Br‐cGMP 200 µmol/L, perfusion ‐ 6 min		
				*V*_0_ (mV)	*I*_L_ (nA)	*V*_0_ (mV)	*I*_L,ns_ (nA)	*I*_SAC_ (nA)	*V*_0_ (mV)	^6^ *I*_L,ns,8Br‐cGMP_ (nA)	^6/C^ *I*_SAC,8Br‐cGMP_ (nA)
Columns	1	2	3	4	5	6	7	8	9	10	11
Clamp steps from *V*_h_ to	−45	5	4	−77 ± 2	+0.218 ± 0.03	−55 ± 2	+0.102 ± 0.03	(−)0.149 ± 0.02	−75 ± 2	+0.139 ± 0.03	(−)0.079 ± 0.01
	−80	5	4		−0.084 ± 0.03		−0.173 ± 0.06	(−)0.122 ± 0.02		−0.113 ± 0.02	(−)0.041 ± 0.01
	−90	5	4		−0.379 ± 0.06		−0.389 ± 0.07	(−)0.033 ± 0.01		−0.320 ± 0.02	(+)0.066 ± 0.03
	+40	5	4		+0.389 ± 0.01		+0.421 ± 0.01	(+)0.036 ± 0.02		+0.342 ± 0.03	(−)0.047 ± 0.02

Continuation of perfusion of stretched cell with 8Br‐cGMP 200 µmol/L + SNAP 200 μmol/L, 3 min			Continuation of perfusion of stretched cell with 8Br‐cGMP 200 μmol/L + SNAP, 200 μmol/L, 6 min		
*V*_0_ (mV)	^3^ *I*_L,ns,8Br‐cGMP+SNAP_ (nA)	^3/C^ *I*_SAC,8Br‐cGMP+SNAP_ (nA)	*V*_0_ (mV)	^6^ *I*_L,ns,8Br‐cGMP+SNAP_ (nA)	^6/C^ *I*_SAC,8Br‐cGMP+SNAP_ (nA)
12	13	14	15	16	17
−75 ± 2	+0.135 ± 0.04^#^	(−)0.094 ± 0.01 ^†^	−77 ± 2	+0.137 ± 0.03	(−)0.092 ± 0.02
	−0.092 ± 0.02^#^	(−)0.037 ± 0.01 ^†^		−0.062 ± 0.02	(+)0.045 ± 0.02
	−0.273 ± 0.02	(+)0.129 ± 0.04^††^		−0.247 ± 0.02	(+)0.155 ± 0.04
	+0.287 ± 0.03^##^	(−)0.092 ± 0.01^††^		+0.275 ± 0.03	(−)0.104 ± 0.01

In further experiments (Figure 13d and Table 18), on the background of cell stretch (triangles vs. circles, in control), 6 min after 8Br‐cGMP introduction (squares), the changes described above were recorded, after which 200 µmol/L SNAP was additionally injected. This lead to even greater inhibition of the ^3^ *I*_L,ns,8Br‐cGMP+SNAP_ and, accordingly, to a decrease in ^3/C^ *I*_SAC,8Br‐cGMP+SNAP_ at −80 and −90 mV (inverted triangles). There were no significant changes after 6 min (rhombus).

Thus, 8Br‐cGMP caused inhibition of the stretch‐induced current (*I*_SAC_), accompanied with partial elimination of the changes of *I*_K1_, while additional administration of SNAP on the background of continued cell stretch caused *I*_L,ns_ inhibition to an even greater extent so that its values became lower than the initial ones.

### 3.16 Involvement of ascorbic acid in modulation of the membrane currents ***I***_L,ns,_ and ***I***_K1_ recorded in $K_{\text{in}}^{+}$/$K_{\text{out}}^{+}$ medium

This series of experiments tested an alternative mechanism for modulation of the inward nonselective cation current, apart from the receptor‐operated mode, in which, NO directly modifies free sulfhydryl groups, induces S‐nitrosylation, and opens the gate of the channel (Liu et al., 2020). An inhibitor of S‐nitrosylation ‐ ascorbic acid (Ohtani et al., 2012) at concentrations 1 and 10 μmol/L was used to demonstrate nitric oxide (NO)‐induced S‐nitrosylation. However, no difference in effect was observed at these concentrations.

**FIGURE 14:**
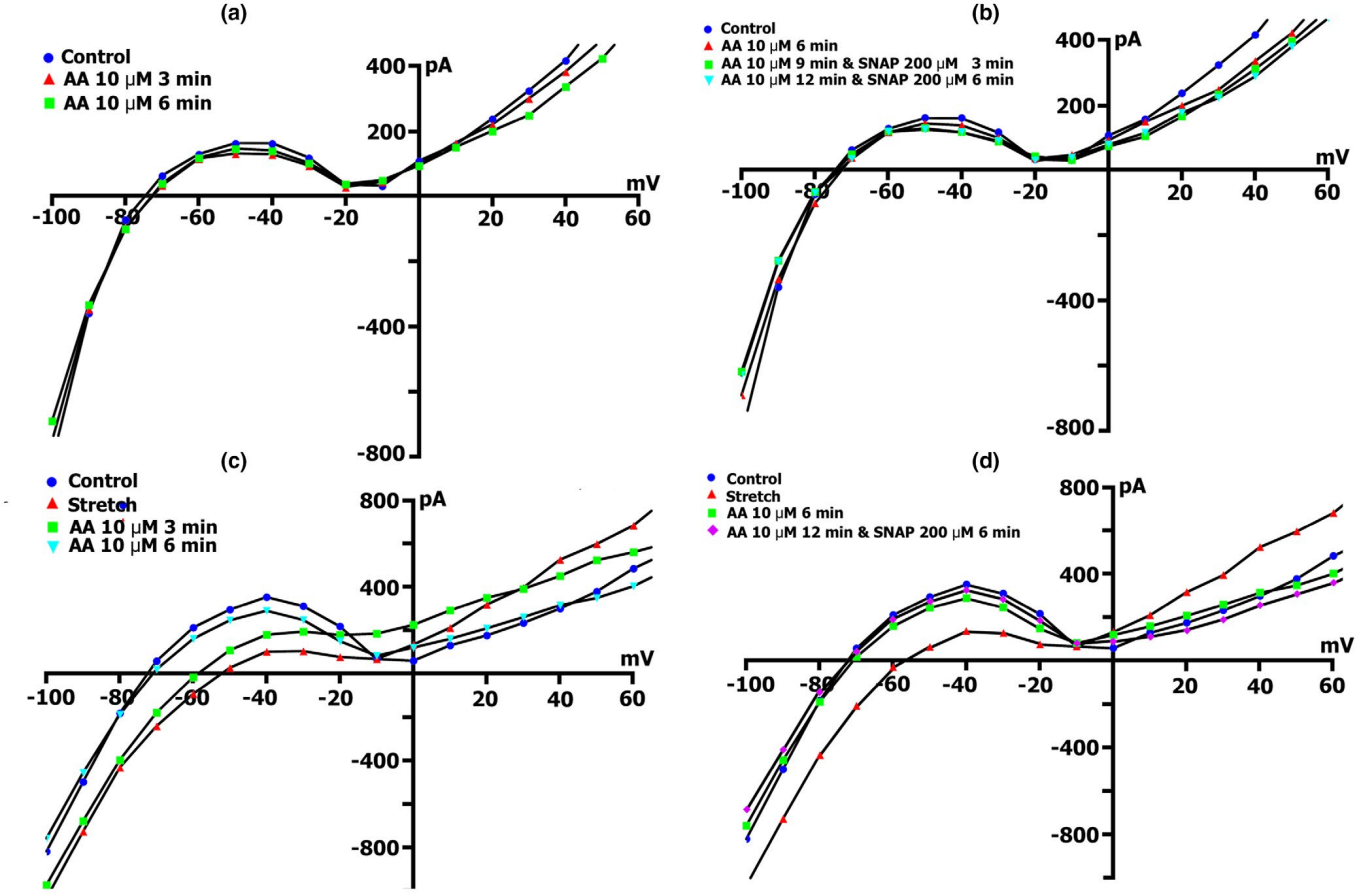
Effect of ascorbic acid (10 μmol/L) and its combination with SNAP (200 μmol/L) on *I*_L_. (a) changes in *I*_L_ in an intact cell on the background of constant perfusion of ascorbic acid in the control (circles), after 3 min (triangles), and after 6 min (squares). (b) changes in *I*_L_ under the action of ascorbic acid with subsequent addition to the SNAP solution. Circles ‐ control, triangles ‐ 6 min of cell perfusion with ascorbic acid, squares ‐ 3 min of perfusion after addition to SNAP solution, inverted triangles ‐ 6 min with SNAP. (c) Changes in *I*_SAC_ in the stretched control cell (circles), after stretching by 6 µm (triangles), after 3 min (squares), and after 6 min (inverted triangles) of constant perfusion of ascorbic acid on the background of stretching. (d) Changes in *I*_SAC_ in the stretched control cell (circles), after stretching by 6 µm (triangles), after 6 min of constant perfusion of ascorbic acid (squares), and after 6 min (rhombuses) of perfusion after adding to the SNAP solution on the background of continuous stretch

Figure 14a shows the *I*/*V* curve of the *I*_L_ current in control and $K_{\text{in}}^{+}$/$K_{\text{out}}^{+}$ medium (circles) and its change under the action of ascorbic acid after 3 (triangles) and 6 min (squares). The average values of *I*_L,KT_, *I*_K1,_ and *ΔI*_KT_ are shown in Table 19. From the data presented, it follows that the perfusion of cells with a solution of ascorbic acid after 3 and 6 min practically did not change either the N‐shaped curve or the magnitude of the currents *I*_K1_, *I*_L,AA,_ or *ΔI*_AA_, nor *V*_0_, although we recorded a trend toward a negligible increase in these currents (Table 19). This is understandable, since it is known that even without mechanical action on cardiomyocytes, because of the cells adhesion to the glass surface of the bottom of the perfusion chamber, a small background amount of *SACs* is open, and ascorbic acid can cause inhibition only of their S‐nitrosylation.

**TABLE 19:** The amplitude of the ascorbic acid (AA) induced current through non−selective cation channels *I*_L,AA_, differential current *ΔI*_AA_, *I*_L,_ and differential current after additional application of SNAP application (*I*_L,AA+SNAP,_ and *ΔI*_AA+SNAP_, respectively), described from *I*/*V* curves of the late current (*I*_L_) at −45, −80, −90, and +40 mV at 10 µmol/L of Ascorbic acid after 3 and 6 min of perfusion and subsequent addition of SNAP against the background of continued Ascorbic acid perfusion. Holding potential (*V*_h_) = −45 mV. *V*_0_ − the intercept of the resulting *I*/*V* curve with the voltage axis defined the zero current potential (*E*_0_) that corresponded to the resting membrane potential of a nonclamped cell (between −70 and −80 mV). $K_{\text{in}}^{+}/K_{\text{out}}^{+}$ solutions. Mean ± SD, *n* = number of experiments (cells), *m* = number of rats. *I*_L_ (nA) − measured value *I*/*V* curves of current. The differential current *ΔI*_AA_ and *ΔI*_AA+SNAP_ that occurs when the values of *I*_L_ are shifted in a more negative direction relative to the reference values are indicated by a minus (−), and the differential current when the values of *I*_L_ are shifted in a more positive direction is indicated by a plus (+). A *p* > 0.05 was considered to indicate a statistically nonsignificant difference (*p* = NS). All other instances with *p* < 0.01 are not indicated. ^#^ *p* = NS versus C10, *p = NS versus C5 and versus C10, **p = NS versus C10 and versus C16, ^##^ *p* = NS versus C16, ^†^ *p* = NS versus C8, versus C14 and versus C17, ^††^ *p* = NS versus C8, ^⊗^ *p* = NS versus C17

Compounds	ς, (mV)	*n*	*m*	Control		AA 10 μmol/L, 3 min perfusion			AA 10 μmol/L, 6 min perfusion		
				*V*_0_ (mV)	*I*_L_ (nA)	*V*_0_ (mV)	^3^ *I*_L, AA_ (nA)	^3/C^ *ΔI*_AA_ (nA)	*V*_0_ (mV)	^6^ *I*_L, AA_ (nA)	^6/C^ *ΔI*_AA_ (nA)
Columns	1	2	3	4	5	6	7	8	9	10	11
Clamp steps from *V*_h_ to	−45	7	4	−76 ± 1	+0.265 ± 0.05	−75 ± 1	+0.229 ± 0.05^#^	(−)0.036 ± 0.005	−77 ± 1	+0.210 ± 0.05	(−)0.054 ± 0.01 ^†^
	−80	7	4		−0.206 ± 0.04		−0.207 ± 0.05*	(−)0.018 ± 0.007		−0.208 ± 0.05	(−)0.012 ± 0.004 ^††^
	−90	7	4		−0.494 ± 0.08		−0.483 ± 0.08*	(+)0.025 ± 0.01		−0.452 ± 0.08	(+)0.042 ± 0.007 ^††^
	+40	7	4		+0.373 ± 0.06		+0.337 ± 0.06^#^	(−)0.039 ± 0.01		+0.325 ± 0.05	(−)0.065 ± 0.01

Continuation of AA 10 μmol/L; SNAP, 200 μmol/L; 3 min perfusion			Continuation of AA 10 μmol/L; SNAP, 200 μmol/L; 6 min perfusion		
*V*_0_ (mV)	^3^ *I*_L,AA+SNAP_ (nA)	^3/C^ *ΔI*_AA+SNAP_ (nA)	*V*_0_ (mV)	^6^ *I*_L,AA+SNAP_ (nA)	^6/C^ *ΔI*_AA+SNAP_ (nA)
12	13	14	15	16	17
−77 ± 1	+0.203 ± 0.04**	(−)0.051 ± 0.01	−77 ± 1	+0.205 ± 0.05	(−)0.046 ± 0.003
	−0.153 ± 0.06	(+)0.045 ± 0.02^⊗^		−0.135 ± 0.06	(+)0.063 ± 0.05
	−0.415 ± 0.09^##^	(+)0.133 ± 0.05^⊗^		−0.406 ± 0.06	(+)0.142 ± 0.06
	+0.320 ± 0.03**	(−)0.105 ± 0.02^⊗^		+0.314 ± 0.04	(−)0.111 ± 0.04

In the next series, 200 µmol/L SNAP was added 6 min after the action of the ascorbic acid. In Figure 14b the *I*/*V* curve of the *I*_L_ current is shown in control (circles), after 6 min of ascorbic acid perfusion (triangles), and again after 3 (squares) and 6 (inverted triangles) min after additional administration of SNAP. The introduction of the NO donor on the background of ascorbic acid must cause a decrease in the *I*_L,AA+SNAP,_ and *ΔI*_AA+SNAP_ values at the levels of −80 and −90 mV, but practically did not affect the *I*_K1_ and output currents at the level of +40 mV (Table 19).

Thus, under the action of ascorbic acid, there was no significant change in the inward nonselective cation current, and further addition of SNAP caused its inhibition.

### 3.17 Ascorbic acid causes stretch‐induced current increase followed by subsequent inhibition

As is shown in Figure 14c, cell stretch by 6 µm causes a stretch‐induced decrease in the *I*_K1_ and an increase in the *I*_L,ns_ (triangles vs. circles in control), which values together with the values of *I*_SAC_ are given in Table 20. Naturally, *V*_0_ shifts toward depolarization. On the background of cell stretch, application of 10 µmol/L ascorbic acid after 3 min caused a decrease of the stretch‐induced (*I*_L,ns,AA_) and differential (*I*_SAC,AA_) current both at −80 and −90 mV (squares) and after 6 min the current values of the ^6^ *I*_L,ns,AA_ and ^6/C^ *I*_SAC,AA_ reached the initial ones (inverted triangles). Also, the *I*_K1_ and *V*_0_ returned to their initial level.

**TABLE 20:** The amplitude of the current through stretch‐activated nonselective cation channels *I*_L,ns_, the differential current through stretch‐activated channels *I*_SAC_, *I*_L,ns_, and *I*_SAC_ after application of Ascorbic Acid (AA) (10 μmol/L) on the background of cell stretching (*I*_L,ns,AA_ and *I*_SAC, AA_, respectively) and after additional application of SNAP (200 μmol/L) against the background of continued perfusion of Ascorbic Acid (*I*_L,ns,AA+SNAP_ and *I*_SAC,AA+SNAP_, respectively) described from *I*/*V* curves of the late current (*I*_L_) at −45, −80, −90 and +40 mV. Holding potential (*V*_h_) = −45 mV. *V*_0_ − the intercept of the resulting *I*/*V*‐curve with the voltage axis defined the zero current potential (*E*_0_) that corresponded to the resting membrane potential of a nonclamped cell (between −70 and −80 mV). $K_{\text{in}}^{+}/K_{\text{out}}^{+}$ solutions. Mean ± SD, *n* = number of experiments (cells), *m* = number of rats. The differential currents *I*_SAC_, *I*_SAC,AA_, and *I*_SAC,AA+SNAP_ that occurs when the *I*_L,ns_ values are shifted to a more negative direction relative to the reference values are indicated by a minus (−), and the differential current when the *I*_L,ns_ values are shifted to a more positive direction is denoted by a plus (+). A *p* > 0.05 was considered to indicate a statistically nonsignificant difference (*p* = NS). All other instances with *p* < 0.01 are not indicated. ^#^ *p* = NS versus C10 and versus C16, ^†^ *p* = NS versus C11 and versus C17, ^††^ *p* = NS versus C17

Compounds	*V*, (mV)	*n*	*m*	Control		Stretch 6 µm			Stretch 6 µm + AA 10 μmol/L, perfusion −6 min		
				*V*_0_ (mV)	*I*_L_ (nA)	*V*_0_ (mV)	*I*_L,ns_ (nA)	*I*_SAC_ (nA)	*V*_0_ (mV)	^6^ *I*_L,ns,AA_ (nA)	^6/C^ *I*_SAC,AA_ (nA)
Columns	1	2	3	4	5	6	7	8	9	10	11
Clamp steps from *V*_h_ to	−45	5	4	−77 ± 2	+0.326 ± 0.04	−55 ± 2	+0.061 ± 0.02	(−)0.255 ± 0.02	−75 ± 2	+0.270 ± 0.03	(−)0.052 ± 0.01
	−80	5	4		−0.198 ± 0.03		−0.458 ± 0.04	(−)0.258 ± 0.03		−0.200 ± 0.03	(−)0.003 ± 0.01
	−90	5	4		−0.502 ± 0.04		−0.743 ± 0.06	(−)0.247 ± 0.04		−0.471 ± 0.04	(+)0.036 ± 0.02
	+40	5	4		+0.307 ± 0.05		+0.523 ± 0.03	(+)0.219 ± 0.03		+0.319 ± 0.05	(+)0.017 ± 0.01

Continuation of perfusion of stretched cell with AA 10 μmol/L + SNAP 200 μmol/L, 3 min			Continuation of perfusion of stretched cell with AA 10 μmol/L + SNAP, 200 μmol/L, 6 min		
*V*_0_ (mV)	^3^ *I*_L,ns,AA+SNAP_ (nA)	^3/C^ *I*_SAC,AA+SNAP_ (nA)	*V*_0_ (mV)	^6^ *I*_L,ns,AA+SNAP_ (nA)	^6/C^ *I*_SAC,AA+SNAP_ (nA)
12	13	14	15	16	17
−75 ± 2	+0.295 ± 0.04^#^	(−)0.029 ± 0.006 ^††^	−77 ± 2	+0.306 ± 0.03	(−)0.018 ± 0.01
	−0.196 ± 0.03^#^	(+)0.001 ± 0.002 ^†^		−0.190 ± 0.03	(+)0.007 ± 0.002
	−0.465 ± 0.04^#^	(+)0.033 ± 0.02 ^†^		−0.460 ± 0.04	(+)0.040 ± 0.02
	+0.310 ± 0.03^#^	(−)0.002 ± 0.003 ^††^		+0.308 ± 0.03	(−)0.002 ± 0.002

Further experiments (Figure 14d and Table 20), on the background of cell stretch (triangles vs. circles in control), after 6 min of the action of ascorbic acid (squares) and registered elimination of the stretch‐induced currents, SNAP was additionally administered, and in the 6th min from its administration, only slight currents magnitude reduction in comparison to the control were noticed (rhombuses).

Thus, our results demonstrate that the inhibitor of S‐nitrosylation, ascorbic acid, eliminates *I*_SAC_ caused by cardiomyocyte's stretch, whereby SNAP added to the medium for 6 min only slightly reduces *I*_L_ in comparison to the control values.

### 3.18 ***I***_L_ currents remain unaffected in the presence of L‐arginin

**FIGURE 15:**
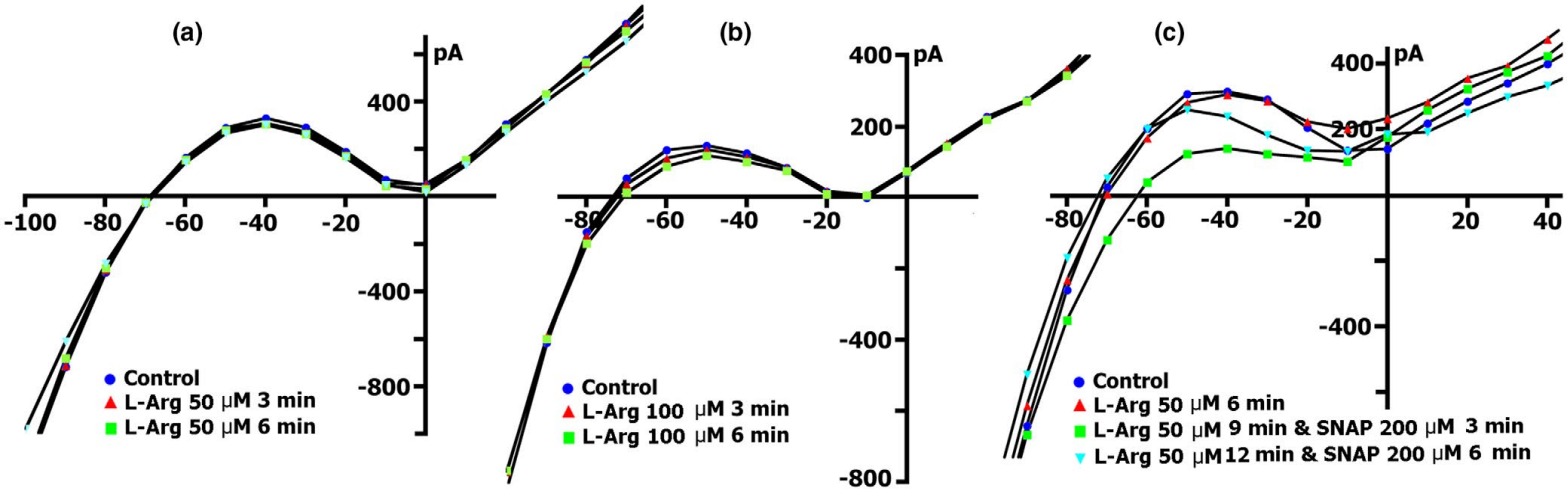
Effect of L‐arginine and its combination with SNAP on the *I*/*V* curve of *I*_L_. (a) Changes in *I*_L_ in an intact cell on the background of its constant perfusion of L‐Arginine at a concentration of 50 μmol/L in the control (circles), after 3 min (triangles), and after 6 min (squares). (b) Changes in *I*_L_ on the background of perfusion with L‐arginine at a concentration of 100 μmol/L in the control (circles), after 3 min (triangles), and after 6 min (squares). (c) changes in *I*_L_ under the action of L‐arginine followed by the addition of 200 μmol/L SNAP to the solution. Circles ‐ control, triangles ‐ 6 min cell perfusion with L‐arginine, squares ‐ 3 min perfusion after addition to SNAP solution, inverted triangles ‐ 6 min with SNAP

Since the absence or significant lack of L‐Arginine in the cell can change the pathways of regulation of *SACs*, we introduced L‐Arginine into the perfusion solution. The addition of L‐Arginine at concentrations of 50 (Figure 15a) and 100 µmol/L (Figure 15b) for 6 min did not change the *I*/*V* curves of the late *I*_L_ currents in intact cells. At the same time, the reaction to additional introduction of 200 µmol/L SNAP into the medium did not change (Figure 15c). During the first 3 min, SNAP increased *I*_L,ns_, and then decreased it to values close to the control ones. Also, the response of the cells to stretching did not change.

## 4 DISCUSSION

### 4.1 Mechano‐electrical feedback determined by *SACs* is regulated by NO

To describe dynamics of mechanical and electrical interaction in the context of the cardiac function, the term "mechano‐electrical coupling" or "mechano‐electrical feedback" (MEF) has been coined and conceptualized as part of cardiac electro‐mechanical autoregulation by Ursula Ravens (née Theophile) in 1967 (Kaufmann & Theophile, 1967) and Max Lab in 1968 (Lab, 1968). The ability of the heart to respond appropriately to changes in the mechanical environment is important for proper cardiac function. The clinical awareness of the mechanical modulation of heart rate and heart rhythm can be traced back to the medical literature, with examples of mechanical induction and mechanical termination of the heart rhythm disturbances (Izu et al., 2020; Orini et al., 2017). In this direction, Craelius, Chen, and El‐Sherif in 1988 for the first time reported for the existence of *SACs* (Craelius et al., 1988); and later the current through these channels (Kamkin et al., 2000, 2003; Zeng et al., 2000), laid the foundation for the mechanisms of the MEF. The *SACs* can be subdivided by their ion selectivity into cation nonselective channels (SACNS) and potassium selective channels (*SACK*) (Takahashi et al., 2013). The molecular identity of the *G*_ns_ has not been categorically determined yet but could be a transient receptor potential (*TRP*) channel or *Piezo 1* (Peyronnet et al., 2016; Reed et al., 2014). A local stretch of cardiomyocytes was shown to induce a current through the nonselective cation channel *TRPC6* (*I*_L,_), which is confirmed by the application of antibodies against *TRPC6* (Dyachenko, Husse, et al., 2009). The membrane currents *I*_L,ns,_ and *I*_K1_ induced by applied modes of mechanical stimulation have been shown to contribute to membrane depolarization and participate in the generation of extra‐systoles (Dyachenko et al., 2008; Kamkin et al., 2000, 2003). It is especially important to note that *SACNS* have a depolarizing effect, while *SACK* has a re/or hyperpolarizing effect on resting cardiomyocytes. Stretch‐activated ion channels are perfectly adapted to integrate changes in the mechanical environment into an altered electrical response. Interestingly, the functional properties of some ion channels can be modified by NO (Boycott et al., 2020; Kazanski et al., 2011; Liao et al., 2006). Furthermore, NOS and NO production is involved in the regulation of every stage of mechanoelectrical feedback: from the initial induction of mechanical signals via integrin/cytoskeleton complexes, through the control of the action potential by modulating the activity of *SACs* and other ion channels, to the mediation of Ca^2+^ processing underlying cardiac contraction. Understanding the dysfunctionality of these processes in pathological conditions accompanied by the complexity of the NO regulation is vital for the optimization of the effectiveness of the therapy based on NO replacement.

### 4.2 Basal intracellular NO is required for the *SACs* function

As shown above, *I*_SAC_ activation requires the presence of intracellular NO, which is formed as a consequence of the NOS activity. The specific NO scavenger PTIO (Dyachenko, Rueckschloss, et al., 2009; Kazanski et al., 2010) (see for review (Kazanski et al., 2011) as well as the NO‐synthase inhibitors N^G^‐Methyl‐L‐arginine acetate salt (L‐NMMA) (Dyachenko, Rueckschloss, et al., 2009) or N^G^‐nitro‐L‐arginine methyl ester (L‐NAME) (Kazanski et al., 2010) (see for review (Kazanski et al., 2011) caused complete inhibition of the *I*_SAC_. Furthermore, to identify the NO‐synthase isoform that generates NO, necessary for activation of the *SACs*, *I*_SAC_ in cardiomyocytes from NOS^−/−^ mice were analyzed (Kazanski et al., 2010). Whereas in wild‐type NOS1^−/−^ and NOS2^−/−^ cardiomyocytes responded to stretch with normal *I*_SAC_, in the cardiomyocytes of NOS3^−/−^ mice, *I*_SAC_ was absent (Kazanski et al., 2010) (see for review (Kazanski et al., 2011). This indicates that NOS3 is the dominant source of NO involved in the *I*_SAC_ activation, which is in agreement with the studies showing that NOS3 is activated by stretch in mouse cardiomyocytes (Petroff et al., 2001).

### 4.3 Intracellular NO level increases during cell stretching

The presented study is not related to the experimental studies associated with the issue of changes in the amount of NO during cell stretching. In this section, we were focused only on the possibility for modulation of the *SAC’s* functions under the action of NO, which concentration may increase with cardiomyocyte stretching, for example, due to Ca^2+^and Na^+^ influx and membrane depolarization that may contribute to NOS activation (Suárez et al., 1999). There are different ways of NOS activation (Dimmeler et al., 1999; Ishida et al., 1997). Here we showed that NO, in case its concentration increases during stretching, caused *SAC’s* modulation and we try to understand the mechanisms of this modulation. To be able to manipulate the systemic NO, we employ the NO donor ‐ SNAP. Briefly, the stretch‐induced *SACs* activation resulted in an NO increase, which in turn, leads to modulation of the *SACs*.

Stretching not only opens the *SACs* but also activates the NOS. NO production increases in response to various mechanical forces, a phenomenon that is of particular importance for cardiovascular function. For example, studies using NO‐sensitive dyes have shown that stretching of ventricular cardiomyocytes induces NO release (Boycott et al., 2020; Shim et al., 2017; Suárez et al., 1999). In some works, 20% sustained stretch was shown to induce an increase in the [NO]_in_ within 5 min. This stretch‐induced elevation in [NO]_in_ is specific to cardiomyocytes, though there is no detectable change in the [NO]_in_, measured in the cardiac fibroblasts (Liao et al., 2006). Quantification of the [NO]_in_ shows rapid and significant stretch‐induced elevation in the [NO]_in_ (135% at 5th min, 121% at 10th min vs. 100% at 0 min) in neonatal rat ventricular myocytes. After the initial transient elevation, the [NO]_in_ tended to recover, but was maintained at a higher level than in the control cells (Liao et al., 2006). According to (Petroff et al., 2001), the cardiac myocyte stretch increases the fluorescence, induced by 4,5‐diaminofluorescein diacetate (DAF‐2) on average about 11%, which is twice as high as the 6% induced in the stretched cells treated by L‐NAME. These data show that NOS activity and endogenous NO production are determined by stretch (Petroff et al., 2001). Of interest are the works in which the authors have shown that increases in coronary flow stimulated NO release in a flow‐dependent fashion, at the same time, infusion of GdCl_3_ decreased NO release at a basal flow and inhibited flow‐induced NO release. An increase in NO release involves activation of NO synthase by increasing calcium/calmodulin (Suárez et al., 1999). The authors hypothesized that the opening of the *SACs* causes Ca^2+^ and Na^+^ influx and membrane depolarization, which may contribute to the activation of NO synthase with the concomitant increase in NO release (Suárez et al., 1999). Later, the influx of Ca^2+^ and Na^+^ through *SACs* and membrane depolarization during the stretching of isolated ventricular cardiomyocytes have been rigorously proven (Kamkin et al., 2000, 2003). However, in similar experiments, other authors have concluded that a brief, repetitive, or sustained increase in coronary perfusion increases cardiac contractility by activating *SACs*, while endothelial NO release is not involved (Lamberts et al., 2002). In other studies, carried out on single cardiomyocytes stretched by carbon fibers, the authors did not find any evidence for NO signaling in the slow inotropic response to stretch (Calaghan & White, 2004), although one existing evidence suggests that NO release upon stretching causes a slow increase in the Ca^2+^ spark frequency in rat's ventricular myocytes stretched within an agarose gel (Petroff et al., 2001). In particular, mechanical stimulus, such as shear stress, is well known to activate NOS via PtdIns‐3‐OH kinase (Dimmeler et al., 1999). However, the extensive studies of the mechanotransduction mechanisms in endothelial (Dimmeler et al., 1999) and smooth muscle cells (Ji et al., 2002), where flow is believed to cause caveolin/NOS signaling activation, showed short‐term elevation in NO, IP_3_, and Ca^2+^, and long‐term induced transcription in the eNOS genes (Belmonte & Morad, 2008; Ishida et al., 1997). One study has shown that the *TRPV1* receptor and *SACs*, respond similarly to mechanical stimuli generated by shear stress, by increasing NO release in the isolated heart (Torres‐Narváez et al., 2012). Other authors have suggested that shear stress may also activate the serine/threonine‐protein kinase Akt/PBB that mediates the activation of eNOS, leading to increased NO production, in a Ca^2+^‐independent manner (Dimmeler et al., 1999; Ishida et al., 1997). Since Ca^2+^ release from the sarcoplasmic reticulum (SR) in cardiac myocytes is regulated through the NOS/NO signaling pathway, independent of Ca^2+^ influx, it has been proposed that *~*18% longitudinal myocyte stretch induces sufficient NO activation to significantly increase the appearance of spontaneous Ca^2+^ sparks and to enhance Ca^2+^ transients and contractility (Barouch et al., 2002; Petroff et al., 2001). Cardiac myocytes subjected to pulses in solution’pressure‐flow’ induce a transient increase in the cytosolic Ca^2+^ through a Ca^2+^‐induced‐Ca^2+^‐release (*CICR*)‐independent mechanic signaling pathway. The results showed that activation of this Ca^2+^ store did not require Ca^2+^ influx through Ca^2+^ channels, *SACs*, or *Sodium*‐*Calcium* exchanger (*NCX)*, nor require significant involvement of NO/NOS or IP3‐R‐gated signaling (Belmonte & Morad, 2008). Recent works in this area are not related to cell elongation. For example, in studies on isolated murine cardiomyocytes loaded with a highly specific copper dye for NO, the authors observed a single transient signal of NO production after any particular event of electrical stimulation (Mosqueira et al., 2021). Application of specific NOS isoform blockers or NO scavengers causes significant NO transient inhibition. The endogenous NOS‐dependent NO is produced transiently after the Ca^2+^‐transient upon electrical stimulation.

Today, the question of whether the amount of intracellular NO increases with the direct stretching of cardiomyocytes remains open. In the face of a significant amount of conflicting data presented by different authors, it is difficult to find an unambiguous explanation. Perhaps the fact is that the authors studied different endpoints of the physiological effect on the applied influences, in which different mechanisms are activated. However, it is possible to study different parts of the NO‐dependent and NO‐independent modulation pathways of *SACs*, and we discuss this in the following sections.

### 4.4 NO effects on *I*_SAC_ under conditions without cell stretching

In an intact cell, using the protocol for *I*_L_ current recording, we register typical *I*/*V* curves for ventricular cardiomyocytes. It is well‐known that, along with many factors, the activity of the ion channels is also determined by the mechanisms associated with the production and use of the NO. Thus, NOS carry out the basic production of NO, which binds to the site in the heme nitric oxide/oxygen binding domain (HNOX) in the β subunit, which causes activation of the catalytic domain of the C‐terminus of this subunit and leads to the production of cGMP and therefore, to the NO‐dependent mechanisms of regulation of the ion channel's activity.

In our experiments, in the absence of cardiomyocyte stretching, on the background of the basal level of [NO]_in_, the exogenous NO reversibly increases *I*_L,_ in conditions without cell stretch.

Previously, we obtained data about the increase in *I*_L,_ under the action of 200 µmol/L SNAP in the first minutes after administration (Kazanski et al., 2010). Another group (Dyachenko, Rueckschloss, et al., 2009) reported that there was no significant increase in *I*_L,_ (*G*_ns_) under influence of the same concentration of SNAP. There is no contradiction in this set of data because in this article, among other things, we reported about the biphasic effect of SNAP at concentrations close to 200 µmol/L (which, according to various sources, leads to release of <2 µmol/L NO Feelisch, 1991; Ioannidis et al., 1996), in time dynamics, and not at randomly chosen points in time, as the authors did earlier (Dyachenko, Rueckschloss, et al., 2009; Kazanski et al., 2010).

First, it should be noted that current activation in the range of 80 to −100 mV was observed in the first minutes of cardiomyocyte superfusion with NO donor SNAP, both in $K_{\text{out}}^{+}$/$K_{\text{in}}^{+}$ and in ${\text{Cs}}_{\text{out}}^{+}$/${\text{Cs}}_{\text{in}}^{+}$ composition. Taking into account the present and pre‐previous data (Kamkin et al., 2000, 2003), *I*_SNAP_, which was activated by NO, is stretch‐activated *I*_L_, since Gd^3+^ at a concentration of 5 µmol/l in $K_{\text{out}}^{+}$/$K_{\text{in}}^{+}$ and ${\text{Cs}}_{\text{out}}^{+}$/${\text{Cs}}_{\text{in}}^{+}$ compositions not only eliminates this current but also caused inhibition of the background *I*_L_ in the range from −80 to −100 mV, shifting *V*_0_ toward hyperpolarization, which is characteristic of the stretch‐activated current. In contrary, preliminary administration of Gd^3+^ markedly inhibits background *I*_L_ in the range from −80 to −100 mV, shifting *V*_0_ toward hyperpolarization, and preventing the development of *I*_SNAP_. Taken together, this allows us to conclude that exogenous NO activates *SAC*s, causing appearance of the *I*_SAC,ns_ without cell stretching. Opening of the mechanosensitive channels under the action of exogenous NO without stretching was shown, for example, for the ryanodine receptor Ca^2+^‐release channels. The exogenously added NO increases the frequency of Ca^2+^‐sparks without cell stretching (Petroff et al., 2001).

In both $K_{\text{out}}^{+}$/$K_{\text{in}}^{+}$ and ${\text{Cs}}_{\text{out}}^{+}$/${\text{Cs}}_{\text{in}}^{+}$ solutions, the biphasic effect of the exogenous NO was recorded in SNAP concentrations close to 200 µmol/L — first causing an increase in the *I*_SAC_,_ns_ without cell stretching, followed by *I*_SAC,ns_ decrease to the initial values and subsequent inhibition, in some cases accompanied by a change in *V*_0_ toward hyperpolarization. The biphasic or reversible effects of NO are well known. For example, a similar reversible effect of exogenous NO was also found in the registration of Ca^2^‐release from ryanodine receptor Ca^2+^‐release channels, which are modulated by cell stretch. The exogenously added NO reversibly increases the frequency of the Ca^2+^‐sparks (Petroff et al., 2001). The biphasic effect was absent with a significant increase in the concentration of exogenous NO to 400 µmol/L SNAP. In this case, inhibition of *I*_SAC,ns_ was observed from the first minutes. In our experiments, this reversible effect after long‐term use of SNAP at a low concentration or by the use of SNAP at a high concentration was reduced to a level of the inward cation nonselective currents described by the *I*_L_ curve. The *I*_L_ values of the currents return to their original values or were even lower than the original ones.

The reason for the inhibition of *I*_L_ in the region of negative potential is unclear, although there is much data about the biphasic effects of exogenous NO, including those presented above (Petroff et al., 2001). Below, we attempt to discuss the cause of the biphasic response (see section 4.10). NO has recently been shown to modulate sodium/hydrogen exchanger‐1 (*NHE1*) in a concentration‐dependent manner through a biphasic effect: *NHE1* is activated at low [NO], but inhibited at high [NO]. These responses involved cGMP‐dependent signaling, rather than S‐nitrosylation (Richards et al., 2020). Furthermore, activation of the *NHE1*‐dependent Na^+^ influx by low [NO] also increased the frequency of spontaneous Ca^2+^ waves, while high [NO] suppressed these aberrant forms of Ca^2+^ signaling. In this case, cGMP was determined to activate *NHE1*, while cAMP was inhibitory, which explains the biphasic mode of regulation by NO (Richards et al., 2020). High levels of NO have also been previously shown to induce a large increase in cGMP and a negative inotropic effect mediated by a PKG‐dependent reduction in myofilament's responsiveness to Ca^2+^. Low levels of NO increases cAMP, at least in part by cGMP‐independent activation of adenylyl cyclase and induce a positive contractile response (Vila‐Petroff et al., 1999). Similar effects have been shown for several cardiomyocyte ion channels. Some of these effects are mediated by cGMP, through the activity of three main proteins as cGMP‐dependent protein kinase (PKG), cGMP‐stimulated phosphodiesterase (PDE2), and cGMP‐inhibited PDE (PDE3). Other effects appear independent of cGMP, such as NO modulation of the ryanodine receptor ‐ *Ca^2^* ^+^ channel. It should be noted that in the case of the cardiac *L*‐*type Ca^2^* ^+^ channel current (*I*_Ca,L_), both cGMP‐dependent and cGMP‐independent effects have been reported, with important tissue and species specificity (Fischmeister et al., 2005). The potential role of the S‐nitrosylation in the biphasic responses is discussed below. The biphasic effect in terms of *I*_L_ inhibition could be considered in terms of some toxic off‐target effects of SNAP (Vejlstrup et al., 1998). However, we are not inclined to consider this possibility in our experiments due to the low concentrations of SNAP and the short time of action of SNAP for the manifestation of the NO off‐target action.

### 4.5 Stretch opens while exogenous NO inhibits *SACs*

Interestingly, application of stretch caused *SACs* opening and increasing of the *I*_SAC,ns_, but subsequent administration of exogenous NO caused *I*_SAC,ns_ inhibition as early as 5 min. In contrary, exogenous NO caused *SACs* activation and appearance of *I*_SAC_,_ns_ under conditions without stretch, whereby subsequent stretch leads to inhibition of *I*_SAC,ns_. If the cell is stretched after the application of SNAP on the background of the onset of inhibition of *I*_SAC,ns_, it leads to temporary activation of the *I*_SAC,ns_, which returns to its original value after 10 min. These effects occur in both $K_{\text{out}}^{+}$/$K_{\text{in}}^{+}$ and ${\text{Cs}}_{\text{out}}^{+}$/${\text{Cs}}_{\text{in}}^{+}$ compositions. Combined, in these experiments, the dependence of the *SAC*s response from the total concentration of NO was traced, and the data obtained correlate well with the data related to the effect of different concentrations of SNAP on *I_SAC_*_,_ *_ns_* under conditions without stretch. To understand the mechanisms of modulation of *SACs* by NO, in addition to the direct use of SNAP for activation of the NO‐dependent sGC pathway, the experiments were carried out to study the effects on intact and stretched cells. For such a purpose the activator of the NO‐independent sGC pathway—BAY41‐2272, the specific blocker of sGC ‐ ODQ, the inhibitor of a cGMP‐dependent protein kinase from the NO‐sGC‐cGMP‐PKG pathway—KT5823, the analog of cGMP—8Br‐cGMP, and the inhibitor of S‐nitrosylation—ascorbic acid were employed. In all cases, SNAP was added to the background of these compounds to cause activation of the NO‐dependent pathway for sGC and/or S‐nitrosylation. Obtained results are discussed below.

### 4.6 In unstretched cells, BAY41‐227 causes *I*_L,ns_ reduction and abolition of the *I*_SAC_ induced by cell stretch

In our experiments, in the unstretched cells, 10 µmol/L BAY41‐2272 reduces the magnitude of the currents at levels −45, −80, and −90 mV, and does not affect the outward current at +40 mV. The inward cation nonselective current, recorded in the negative range of currents at potentials more negative than *V*_0_, makes the main contribution to the *I*_SAC_. It was the only current component that was reduced. SNAP administered on the background of BAY41‐2272, caused a biphasic effect, i.e, an increase in the *I*_L,ns_, followed by its inhibition. In contrary to NO, which binds to a site in the β subunit, causing activation of its C‐terminal catalytic domain and leading to the production of cGMP, which further results in the operation of the NO‐dependent NO‐sGC‐cGMP‐PKG pathway for regulation of the channel activity, the mechanism of action of BAY 41‐2272 is opposite. Actually, by binding to the NO‐independent regulatory site of the α1 sGC subunit, BAY 41‐2272 causes activation of the catalytic domain of the C‐terminus of this subunit, leading to additional cGMP production (Stasch et al., 2001) and, ultimately, to activation of PKG and possible phosphorylation of the *SACs* along the pathway sGC‐cGMP‐PKG. Based on the classical sGC‐cGMP‐PKG pathway, the NO‐independent pathway of cGMP activation lead to phosphorylation of the *SACs*, followed by inhibition of the *I*_L,ns_, while the introduction of SNAP caused temporary *I*_L,ns_ activation. In this case, there are two scenarios for the development of events, and today there is no unambiguous answer which path will be used. First, BAY 41‐2272 is already associated with a site in the α1 subunit. If NO can also bind to a site in the β subunit and this binding results in an additional increase in cGMP, then the transient effect of SNAP could be explained by the sGC‐cGMP‐PKG pathway. Such a course of events seems possible in case we applied BAY 58‐2667. In the case of a combination between BAY 58‐2667 and DEA/NO over the entire range of used concentrations, an additive effect on the sGC activity was observed. These observations have been repeated with higher and lower concentrations of DEA/NO and remained additive (Stasch et al., 2002). However, there is no evidence to support a similar situation for BAY 41‐2272. Second, if on the background of BAY 41‐2272, NO cannot additionally bind to its ‘own’ site in the β subunit, then a temporary increase in the *I*_L,ns_ could be “taken” as a consequence of the channel S‐nitrosylation.

However, most importantly, BAY41‐2272 abolished the inward stretch‐induced current components of *I*_SAC_, while additional administration of SNAP caused an *I_K_*_1_ increase to control values, with further reduction in *I*_L,ns_. Additionally, we hypothesized that the effect of BAY41‐2272 on the NO‐cGMP‐PKG pathway probably induces phosphorylation of the *SACs*, opened by cell stretch, which can lead to the complete abolition of the stretch‐induced *I*_SAC_. The subsequent additional administration of SNAP did not change *I*_L,ns_, which is also understandable from the previously obtained data showing that the administration of SNAP on the background of stretch causes *I*_SAC_ elimination.

### 4.7 ODQ causes current modulation in both unstretched and stretched cells

In our experiments, ODQ caused *I*_L,ns_ decrease in the unstretched cells and shifts *V*_0_ toward hyperpolarization. SNAP introduced into the solution in the presence of ODQ, caused *I_L_*_,_ *_ns_* increase by hyperpolarizing *V*_0_. After that, SNAP caused a second *I*_L,ns_ decrease, shifting the curve to a more negative range. This biphasic reaction is characteristic of pure SNAP. ODQ caused the elimination of the stretch‐induced *I*_SAC_, whereby additional administration of SNAP returned them, but not to the control values.

Note that ODQ binds to the sGC in a specific way. The sGCα subunit, which binds to the common sGCβ_1_ subunit, exists in two different isoforms, sGCα_1_ and sGCα_2_ (Sips et al., 2011). Isoform sGCα_1_β_1_ is predominantly expressed in the ventricular cardiomyocytes (Cawley et al., 2011), but it is important to note that ODQ can inhibit all isoforms of sGC (Sips et al., 2011). In an intact cell, inhibition of sGC is followed by deregulation in the channel's activity via the NO‐cGMP‐PKG pathway, which operates through basal NO production by NOS. Although there is a growing body of data obtained by the employment of ODQ as a specific inhibitor of the sGC and NO‐cGMP‐PKG pathway, the results obtained by the use of this compound should be treated with caution, since it is known that in addition to its action on the sGC, ODQ has the ability to affects organic nitrates and acts on the cytochrome P‐450 enzyme system. More importantly, despite its ability to block sGC, ODQ also can cause a block of the NOS by its metabolic conversion (Feelisch et al., 1999). Hence, for our task, the parallel blocking of NOS through the metabolic transformation of ODQ is even more interesting. In this case, perfusion of ODQ turns off not only NO‐cGMP‐PKG signaling but probably the activity of NOS i.e the formation of NO. Under these conditions, the pronounced activation of *I*_L,ns_ by SNAP on the background of pharmacological blockage of the sGC induced by ODQ, quite convincingly demonstrates the predominant role of the *SACs*‐nitrosylation. It is also important to note that, on the background of ODQ, SNAP retains its biphasic activity.

Stretching of the cell causes opening of the *SACs* and appearance of the stretch‐induced currents *I*_L,ns_, the value of which *ΔI*_SAC_ at a given level of stretch remains constant, that is, without adaptation. The use of ODQ causes *ΔI*_SAC_ elimination which is difficult to be explained only by sGC‐cGMP‐PKG pathway blocking, although several authors have shown that the operation of *SACs* is determined by the level of NO in the cell. Thus, the use of NO scavenger PTIO, the NO synthase inhibitor L‐ NAME (Dyachenko, Rueckschloss, et al., 2009; Kazanski et al., 2010 and Kazanski et al., 2011), and the NOS3^−/−^ knockout mice (Kazanski et al., 2010; Makarenko et al., 2012), confirm that in the absence of NO, stretch by itself cannot lead to *ΔI*_SAC_. On the other hand, an increase in the concentration of NO by introducing SNAP or DEA‐NO into the medium on the background of stretch caused *ΔI*_SAC_ elimination (Kazanski et al., 2010). However, the evidence that the metabolic transformation of ODQ turns it into an inhibitor of NOS (Feelisch et al., 1999), leads to the conclusion that ODQ causes *ΔI*_SAC_ elimination not only due to blockage of the sGC‐cGMP‐PKG pathway but due to the blockage of the NOS, which is the reason of the reduced amount of NO in the cell. The last was confirmed by the response of the stretched cell to SNAP introduced on the background of ODQ, which resulted with increasing in the inward nonselective cation current. Thus, the assumption about the S‐nitrosylation of the *SACs* seems quite convincing.

### 4.8 KT‐5823 causes *I*_L,ns_ inhibition in nonstretched cells but transiently increases *I*_SAC_

KT5823, known to be an inhibitor of the cGMP‐dependent protein kinase, in the sGC‐cGMP‐PKG pathway, should decrease phosphorylation in the unstretched cells and presumably cause an increase in the current; however, we recorded a decrease in the *I*_L,ns_. This contradiction is probably based on other mechanisms associated with the differences not only in the gating mechanisms but also in the principles of its regulation, for example, in voltage‐gated or stretch‐activated channels. It has been shown that many ion channels exhibit mechanosensitivity and have corresponding gating mechanisms. These include *TRPC1*, *TRPC5*, and *TRPC6* (Gomis et al., 2008; Kerstein et al., 2013; Sharif‐Naeini et al., 2008; Sharif‐Naeini et al., 2008). At the same time, through the example of the mechanosensitive channel *TRPC6*, the possibility of increasing and decreasing its conductivity during phosphorylation was shown. Phosphorylation was shown to potentiate the channel conductance. Modification may occur in basal status or after *TRPC6* activation (Hisatsune et al., 2004; Shi et al., 2013). On the other hand, phosphorylation has been shown to negatively regulate *TRPC6* through the NO‐cGMP‐PKG pathway (Takahashi et al., 2008). As shown (Dyachenko, Husse, et al., 2009; Dyachenko, Rueckschloss, et al., 2009), *TRPC6* channels are the most likely candidates for the formation of *I*_L,ns_, and *I*_SAC_ in the ventricular cardiomyocytes. In these studies, authors applied isolated cardiomyocyte stretching techniques that we described in our earlier papers (Kamkin et al., 2000, 2003) and applied in this study.

In our experiments, the addition of SNAP on the background of KT5823, first increases and then decreases *I*_L,ns_, which is consistent with the biphasic effect of NO, i.e increase the conductivity of *SACs* due to the possible S‐nitrosylation in the first phase. However, the implementation of the second phase through NO‐sGC‐cGMP‐PKG signaling is impossible due to the PKG blockade. The PKG inhibitor does not interfere with the activation of the sGC and increase in the cGMP due to the presence of SNAP in the environment. Interestingly, KT5823 is not involved only in the *I*_SAC_ suppression, but in opposite, temporarily even increases it, that is, does exactly what was expected. After this increase, *I*_SAC_ inhibition was observed, and SNAP administration caused even stronger *I*_L,ns_ reduction. This is understandable since SNAP could not manifest the first phase during the cell stretch. Precisely, on the background of stretch, SNAP completely blocks *I*_SAC_.

### 4.9 8Br‐cGMP causes *I*_L,ns_ inhibition in unstretched cells but decreases *I*_SAC_

In the unstretched cells, the analog of cGMP, called 8Br‐cGMP, caused inward cation nonselective current *I*_L,ns_ increase at −80 mV, shifting *V*_0_ toward depolarization. As shown in Section 4.8, the most likely candidates that provide formation of *I*_L,ns_, and *I*_SAC_ in our experiments are *TRPC6* (Dyachenko, Husse, et al., 2009; Dyachenko, Rueckschloss, et al., 2009), which can increase or decrease its conductivity during phosphorylation (see Section 4.8). This feature may be because KT‐5823 cause *I*_L,ns_ decrease in the unstretched cells (see Section 4.8), while 8Br‐cGMP increased this current. SNAP caused 8Br‐cGMP‐induced current elimination, in a similar way to how SNAP eliminates *I*_SAC_.

The introduction of 8Br‐cGMP into the medium caused inhibition in the stretch‐induced current *ΔI*_SAC_, as it should be due to the activation of PKG. Interestingly, the current inhibition only affected *I*_SAC_, which appeared in the background of cell elongation. It seems there is an analogy with the action of KT‐5823 (see Section 4.8), whose introduction into the medium caused PKG inhibition, which lead to an increase in the *I*_SAC_. Hence, both KT‐5823 and 8Br‐cGMP had a characteristic effect only on the cell stretch‐induced *I*_SAC_, acting oppositely on the *I*_L,ns_, if it is not activated by cell stretch. Upon perfusion of 8Br‐cGMP on the background of cell elongation, additional administration of SNAP caused *I*_L,ns_ inhibition to an even greater extent, and its values became lower than the initial ones. We considered this reaction of the cell as a typical reaction in its elongated state.

### 4.10 Ascorbic acid as an S‐nitrosylation inhibitor of *SACs*, causes *I*_SAC_ elimination

In the last 10 years, it has become clear that NO appears to be the main modulator of the mechanotransduction pathways in the heart through direct nitrosylation or activation of PKG. Taken together, our results indicated that endogenous NO production by cardiac myocytes, when they are stretched, caused direct modulation of *SACs*. We propose that the resultant production of NO exerts its action independently of cGMP, most probably through S‐nitrosylation.

This is largely confirmed by our experiments, in which, in unstretched cells, the inhibitor of S‐nitrosylation, ascorbic acid, practically did not change the inward cation nonselective current *I*_L,ns_, and further addition of SNAP caused its inhibition from the first minute. In this case, we can assume that the lack of SNAP effect is primarily due to the blockade of S‐nitrosylation. Furthermore, the biphasic effect of SNAP (see Section 4.4) can be determined by S‐nitrosylation of *SACs*, resulting in a transient SNAP‐induced current that is blocked by Gd^3+^ and is analogous to *I*_SAC_. In any case, it is based on *I*_L,ns_, which inhibition occurs 3–5 min after activation, and is associated with the NO‐sGC‐cGMP‐PKG pathway induction and phosphorylation of *SACs*, leading to a decrease in their conductivity. However, the main thing is that the inhibitor of S‐nitrosylation causes elimination of the stretch‐induced *I*_SAC_, while SNAP only slightly reduces *I*_L,ns_ in comparison to the control. The complete elimination of *I*_SAC_ by ascorbic acid can be explained primarily by blocking the *SACs’* nitrosylation. In this case, SNAP cannot express its activating effect and induces only time‐delayed inhibition of the *I*_L,ns_.

NOS‐derived NO exerts its effects in a variety of ways. For example, NO can post‐translationally modify target proteins primarily through the addition of a nitroso group to the sulfhydryl side chain of cysteine, the process called S‐nitrosylation (Kovacs & Lindermayr, 2013). Such a modification results in an alteration in the function of the target protein (Gonzalez et al., 2009). The effects of direct NO modification of target proteins are limited by the relatively short diffusion distance of the molecule. Therefore, NO has to be synthesized close to its targets via coordinated signaling (i.e., by S‐nitrosylation), because of its extremely short half‐life (microseconds). Otherwise, NO is rapidly scavenged by myoglobin (Flögel et al., 2001), or oxidized to NO_2_^−^. Cellular carrier molecules such as S‐nitrosoglutathione mediates long‐range NO signaling by acting as a carrier and donor, transferring NO to more distal targets (Such‐Miquel et al., 2018). The functional properties of some ion channels can be modified by NO, which can occur by adding a nitroso group to a thiol (Gonzalez et al., 2009), for example, to a cysteine residue. Such nitrosylation can have the effect of enhancing the channel activity or inhibiting it, depending on the target channel. In particular, several channels are mechanically sensitive to and modulated by NO, such as the voltage‐dependent K^+^ channel‐1.5 (*K*_V_1.5) (Nunez et al., 2006), the voltage‐dependent Ca^2+^channel‐1.2 (*Ca*_v_1.2) (Campbell et al., 1996), and the voltage‐dependent *Na* ^+^ channel‐1.5 (*Na*_v_1.5) (Ueda et al., 2008). Certain ion channels, for example, *K*_V_1.5, can be modulated by NO through both direct nitrosylation and the PKG pathway (Nunez et al., 2006) (see for review (Kazanski et al., 2011).

### 4.11 Exogenous L‐Arginine does not affect *I*_L,ns,_ and *I*_SAC_

The L‐arginine is the substrate for NOS to produce NO. Unlike some other tissues, cardiomyocytes are not able to synthesize L‐arginine (like the liver or kidneys) (Hattori et al., 1995) or to recycle L‐arginine from citrulline (like the endothelium) (Nagasaki et al., 1996). At the level of cardiac myocytes, L‐arginine is incorporated into the circulation through the function of systemic ‐ y^+^ cationic amino acid transporters (Devés & Boyd, 1998; Ramachandran & Peluffo, 2017). Depletion of L‐arginine leads to NOS uncoupling with O_2_ rather than L‐arginine as a terminal electron acceptor, resulting in the formation of superoxide. Reactive oxygen species (ROS) or superoxide ($O_{2}^{‐}$), combined with NO, can lead to the production of reactive nitrogen species (RNS), or even peroxynitrite (ONOO^−^) (Ramachandran & Peluffo, 2017). Peroxynitrite has been shown to induce MSC‐like changes in membrane currents in isolated ventricular cardiomyocytes (Dyachenko, Husse, et al., 2009). On the one hand, the formation of peroxynitrites may cause phospholipases activation and generation of amphipaths that modulate channel function by changing the curvature of the surrounding lipid bilayer. On the other hand, peroxynitrite could directly affect *TRPC6* channels (Dyachenko, Husse, et al., 2009). Ramachandran and Peluffo, (2017), showed that below a threshold value of ~100 µmol/L, decreasing concentrations of L‐arginine causes a progressive increase in the ONOO^−^/O^2−^‐induced fluorescence. These results provide an estimate of the levels of circulating L‐arginine below which, the ROS/RNS‐mediated harmful effects arise in cardiac muscle (Ramachandran & Peluffo, 2017). The L‐arginine leads to an increase in the inward currents in cardiomyocytes. However, at physiological concentrations (~100 µmol/L), this current is difficult to be distinguished from the background noise and can hardly affect all electrophysiological parameters (Peluffo, 2007).

Interestingly, background NO production is also observed in the absence of L‐arginine application, probably due to the presence of an internal pool of L‐arginine in the cardiomyocytes (Peluffo, 2007). Indeed, the Michaelis constant for NOS (*k*_m_ for rat nNOS according to L‐arginine is 1.5 µmol/L [Bredt & Snyder, 1990] or 3.3 µmol/L (Schmidt et al., 1992), and according to various sources, for eNOS in humans it is 1 µmol/L (Garvey et al., 1994). The last values confirm the view that micromolar intracellular concentrations of L‐arginine are sufficient for NO production.

Since the absence or significant lack of L‐Arginine can change the pathways of regulation of *SACs*, we injected L‐Arginine into the perfusion solution at a concentration of 50 to 100 µmol/L. The addition of L‐arginine practically did not change the *I*/*V* curves of the late *I*_L_ currents in intact cells. Also, the response of the cells to stretch did not change. In addition, under control conditions, we always recorded the standard *I*/*V* curve of the late current *I*_L_. This curve would change the *N* shape, or the shape of the portion of the curve reflecting inward cation nonselective currents, if there were no or insufficient levels of external *L*‐arginine that trigger ROS/RNS production in cardiac myocytes.

### 4.12 Other ways of *I*_L,ns_ activation, and *SACs* modulation

It should be noted that PKG can be activated not only by cGMP but also by various oxidizing agents, as reactive oxygen species (ROS) (Burgoyne et al., 2007). In case of such activation, PKG is transformed into disulfide form and the substrate of its action changes. Particularly, it ceases to phosphorylate and consequently reduces the activity of RhoA (a protein in the family of small GTPases) (Prysyazhna et al., 2016). cGMP increases the proportion of phosphorylated RhoA. Physiological stretching rapidly activates a reduced form of nicotinamide adenine dinucleotide phosphate (NADPH) oxidase 2 (NOX2), to produce reactive oxygen species (ROS) (Prosser et al., 2011). NOX2 and NOX4 are the most abundantly expressed isoforms of NOX in adult cardiomyocytes (Byrne et al., 2003). NOX2 and NOX4 form superoxide, which can directly affect *I*_Cl,swell_, and interact with NO from NOS3, forming peroxynitrite, which can affect *TRPC6* and *K*_ir_2.3 through PLC and PLA2 (Dyachenko, Husse, et al., 2009). In general, this is important for two reasons: first, a mechanical stretch of cardiomyocytes has been shown to lead to activation of NOX2, leading to an increase in ROS and as a consequence, changes in the redox state of the cell (Prosser et al., 2011). This may influence the proportion of PKG activated by oxidation since the disulfide state of PKG has been shown to exist even under normal cellular conditions (Prysyazhna et al., 2016). Second, sGC ‐ activators and inhibitors naturally affect the concentration of cGMP and, as a result, can affect the balance of normal and disulfide forms of PKG, especially when the redox state of the cell is altered. These paths we hope to explore later.

## 5 CONCLUSION

In summary, mechanical stimulation of rat ventricular myocytes by local stretch modulates membrane currents *I*_L,ns,_ and *I*_K1_ and activates *TRPC6* channels (mediating *I*_L,ns_) or deactivates *K*_ir_2.3 channels (mediating *I*_K1_) (Dyachenko, Husse, et al., 2009; Dyachenko, Rueckschloss, et al., 2009). This resulted in a stretch‐activated current *I*_SAC_, which is modulated via a complex signaling cascade. The membrane stretch‐activated currents induced by our mode of mechanical stimulation have been shown to contribute to membrane depolarization and generate extrasystoles (Kamkin et al., 2000, 2003). Therefore, it was important to evaluate the possibility of NO, including NO‐dependent and NO‐independent pathways of the sGC and S‐nitrosylation activation in the regulation of *SACs*. The S‐nitrosylation, is the most important component since cardiac ion channels that serve the excitation‐contraction coupling are potentially regulated by S‐nitrosylation. S‐nitrosylation signaling is disrupted in pathological states in which the redox state of the cell is dysregulated, including ischemia, heart failure, and atrial fibrillation (Gonzalez et al., 2009).

Based on the data obtained and the analysis of the literature, we can conclude that the physiological concentration of NO in the cell is a prerequisite for the operation of *SACs*, since binding of NO (Dyachenko, Rueckschloss, et al., 2009; Kazanski et al., 2010b; Makarenko et al., 2012), blocking of NOS (Dyachenko, Rueckschloss, et al., 2009; Kazanski et al., 2010b; Makarenko et al., 2012) or the use of NOS3^−/−^ mice, eliminates the effects induced by cell stretch or prevented them from developing (Kazanski et al., 2010b; Makarenko et al., 2012). An increase in the NO concentration as a result of the exogenous addition of donors on the background of stretch caused *I*_SAC_ elimination.

Exogenous NO may include not only NO‐dependent pathway for *SACs* modulation but also S‐nitrosylation of *SACs*. In an intact cell, NO leads to a two‐phase effect: a short activation phase of the Gd^3+^ sensitive cation nonselective current *I*_L,ns,_ and a longer phase of inhibition of this current. The short activation phase is probably associated with the S‐nitrosylation of *SACs*. Furthermore, a longer phase of inhibition of *I*_L,ns,_ can be determined by NO‐dependent regulation of the channel activity, in which phosphorylation of *SACs* reduces their conductivity. The inhibitor of S‐nitrosylation, ascorbic acid, abolishes the short phase of *I*_L,ns_ activation induced by the NO donor, but retains the second phase of the *I*_L,ns_ inhibition via the NO‐dependent pathway. Moreover, ascorbic acid completely abolishes *I*_SAC_ caused by cell elongation, and under these conditions, exogenous NO does not lead to the appearance of the first phase. It is important to note that the NO donors without cell elongation induce *I*_L,ns_, which is equivalent to *I*_SAC_, and on the background of cell elongation, the exogenous NO abolishes *I*_SAC_. Activation of the NO‐independent sGC‐cGMP‐PKG pathway by BAY41‐2272 did not induce the initial S‐nitrosylation of the *SACs*, because there was no exogenous NO, and there was no primary activation of the *I*_L,ns_, but as a result of the PKG activation, the subsequent phosphorylation of *SACs* caused *I*_L,ns_ decrease. The exogenous NO on the background of BAY41‐2272 induced the first phase of increase in *I*_L,ns_ followed by its inhibition, which corresponds to a two‐phase reaction of pure SNAP. BAY41‐2272 abolishes *I*_SAC_, probably as a consequence of PKG activation and subsequent phosphorylation of *SACs*. However, SNAP on the background of stretching cannot cause further *I*_SAC_ increase.

The blocker of the sGC, ODQ turns off the sGC‐cGMP‐PKG pathway, leading to inhibition of *I*_L,ns_. However, SNAP introduced into the medium can induce S‐nitrosylation of the *SACs*, followed by induction of the first phase: an increase in *I*_L,ns_ followed by its inhibition. ODQ, by blocking the sGC, reduces the activity of PKG and consequent phosphorylation; *I*_L,ns_ decreases, probably due to the conversion of ODQ into an inhibitor of NOS as a result of metabolic transformation. ODQ also eliminates *I*_SAC_. However, SNAP added on the background of stretch, caused *I*_SAC_ increase in addition to ODQ, which can occur only under NO deficient conditions as a result of NOS inhibition by altered ODQ.

The PKG inhibitor KT5823 reduces PKG activity and reduces *SACs* phosphorylation, leading to a transient increase in *I*_SAC_, whereby the introduction of SNAP reduces *I*_L,ns_ to an even greater extent since the cell was initially stretched. 8Br‐cGMP reduces *I*_SAC_, as it should, by activating PKG and subsequent phosphorylation. Similarly, KT‐5823, through inhibition of PKG, caused an *I*_SAC_ increase. Therefore, KT‐5823 and 8Br‐cGMP have a characteristic effect on *I*_SAC_ induced by cell stretch.

Finally, the results of our study demonstrate a significant contribution of the S‐nitrosylation to the regulation of the *SACs*. Studying the role of this mechanism in cardiomyocytes is essential since S‐nitrosylation signaling is disrupted in pathological states in which the cell's redox state is dysregulated, including ischemia, heart failure, and atrial fibrillation (Gonzalez et al., 2009). At the same time, the sGC‐cGMP‐PKG pathway also plays a very important role in the activity of the *SACs*, but apparently with some delay in time. We also do not rule out other ways of regulating the operation of the *SACs* under certain conditions. For example, the effect of reactive oxygen species (ROS) should be additionally studied.

## 6 STUDY LIMITATION

One of the basic limitations of this study was the inability to consider the fact that physiologic stretch rapidly activates a reduced form of NOX2 to produce ROS in a process dependent on microtubules (X‐ROS signaling) (Prosser et al., 2011). ROS production occurs in the sarcolemmal and t‐tubule membranes where NOX2 is located and sensitizes nearby ryanodine receptors (RyRs) in the sarcoplasmic reticulum (SR). Precisely we hope that the fact that derives from the proximity between NOX2, and RyRs, together with the stretch‐dependent "tuning" of the RyRs will provide a mechanistic explanation for the X‐ROS related mechanotransduction. The relationship between these signaling pathways and stretch‐induced sGC‐involved mechanisms could be particularly interesting and deserve special attention. Based on all the above, it seems that this can involve a serious set of experiments which will be addressed in another study.

## CONFLICT OF INTEREST

None declared.

## AUTHOR CONTRIBUTION

A.K., O.K., and V.K. contributed to the conception and design of this study; V.K., A.B., A.S., T.F., D.A., and V.M. developed and performed the experiments; A.K., O.K., and M.M. prepared and interpreted the tables and figure plots; the first draft of the manuscript was written by A.K.; all authors read and approved the final version and agree to be accountable for all aspects of this work.

## Data Availability

Raw data were generated at [Department of Physiology, Pirogov, Russian National Research Medical University]. Derived data supporting the findings of this study are available from the corresponding author on request.
